# Mobile-Web App to Self-Manage Low Back Pain: Randomized Controlled Trial

**DOI:** 10.2196/jmir.3130

**Published:** 2015-01-02

**Authors:** A Blair Irvine, Holly Russell, Michael Manocchia, David E Mino, Terri Cox Glassen, Rebecca Morgan, Jeff M Gau, Amelia J Birney, Dennis V Ary

**Affiliations:** ^1^ORCASEugene, ORUnited States; ^2^Cigna HealthcareAnalyticsBloomfield, CTUnited States; ^3^University of North FloridaSociology, Anthropology and Social WorkJacksonville, FLUnited States; ^4^Cigna HealthcareOrthopaedic Surgery and Spinal DisordersBlue Bell, PAUnited States; ^5^Cigna HealthcareClinical Program DirectorGlendale, CAUnited States; ^6^Oregon Research InstituteEugene, ORUnited States

**Keywords:** low back pain, Internet, mobile, app, computers, prevention, self-treatment

## Abstract

**Background:**

Nonspecific low back pain (NLBP) is the diagnosis for individuals with back pain that has no underlying medical cause (eg, tumor, infection, fracture, herniated disc, spinal stenosis). The American College of Physicians (ACP) and American Pain Society (APS) recommend multidisciplinary treatments for NLBP that lasts more than 4 weeks. This approach, however, is impractical for many physicians to implement, and relatively few providers offer NLBP treatment that meets the joint ACP-APS guidelines.

**Objective:**

This study evaluated the efficacy of a mobile-Web intervention called “FitBack” to help users implement self-tailored strategies to manage and prevent NLBP occurrences.

**Methods:**

A total of 597 adults were recruited, screened, consented, and assessed online at baseline, at 2 months (T2), and at 4 months (T3). After baseline assessments, participants were randomized into three groups: FitBack intervention, alternative care group that received 8 emails urging participants to link to six Internet resources for NLBP, and control group. The FitBack group also received weekly email reminder prompts for 8 weeks plus emails to do assessments. The control group was only contacted to do assessments.

**Results:**

Users of the FitBack program showed greater improvement compared to the control group in every comparison of the critical physical, behavioral, and worksite outcome measures at 4-month follow-up. In addition, users of the FitBack program performed better than the alternative care group on current back pain, behavioral, and worksite outcomes at 4-month follow-up. For example, subjects in the control group were 1.7 times more likely to report current back pain than subjects in the FitBack group; subjects in the alternative care group were 1.6 times more likely to report current back pain at 4-month follow-up. Further, the users of the FitBack program showed greater improvement compared to both the control and alternative care groups at 4-month follow-up on patient activation, constructs of the Theory of Planned Behavior, and attitudes toward pain.

**Conclusions:**

This research demonstrated that a theoretically based stand-alone mobile-Web intervention that tailors content to users’ preferences and interests can be an effective tool in self-management of low back pain. When viewed from the RE-AIM perspective (ie, reach, efficacy/effectiveness, adoption, implementation fidelity, and maintenance), this study supports the notion that there is considerable value in this type of intervention as a potentially cost-effective tool that can reach large numbers of people. The results are promising considering that the FitBack intervention was neither supported by professional caregivers nor integrated within a health promotion campaign, which might have provided additional support for participants. Still, more research is needed on how self-guided mobile-Web interventions will be used over time and to understand factors associated with continuing user engagement.

**Trial Registration:**

Clinicaltrials.gov NCT01950091; http://clinicaltrials.gov/ct2/show/NCT01950091 (Archived by WebCite at http://www.webcitation.org/6TwZucX77).

## Introduction

Nonspecific low back pain (NLBP), defined here as temporary back pain with no medical signs of a serious underlying condition (eg, cancer, infection, fracture, spinal stenosis) [1], is a pervasive and expensive public health problem in the United States [2-4], experienced by four out of five adults at some point in their lives [5,6]. Back pain is the leading cause of work-related disability and one of the most frequent reasons patients visit a doctor [7,8]. Costs incurred by US back pain sufferers are staggering, estimated at US $90.7 billion [9] and growing [2,10]. People with back pain spend 60% more on health care than those without back pain [10,11]. Most people with low back pain do not visit a physician [12,13] because episodes of NLBP resolve spontaneously [14], but of those who do see a doctor, 30% experience pain and disability a year later [15] and few return to normal activities [14].

Businesses lose 100 million work days per year [11], with back pain accounting for 5.5% of *all* productivity loss in the United States—about US $2,200 per employee per year [16,17]. Even employees with minor back pain lose 4.6 hours of productivity a week due to decreased performance on the job [18,19]. Beyond its economic toll, NLBP causes significant physical and psychological suffering [20,21].

Although no consensus has been reached about the best treatment for NLBP, multidisciplinary approaches reduce employee sick leave [22-24] and are cost-effective [25,26], and early NLBP management is the best approach to preventing chronic back pain [14]. Individuals who experience an episode of acute NLBP can become caught in a cycle of chronic pain and disability if they avoid appropriate activity in fear of exacerbating their pain [27,28]. Recommended NLBP treatments often involve specialized clinics, which are costly and not widely available [6,26,29,30], and insurance companies often don’t cover multidisciplinary treatments [15,31]. The Joint Clinical Practice Guidelines from the American College of Physicians and American Pain Society [1,32] recommend inclusion of psychosocial assessments and multidisciplinary treatments that last more than 4 weeks for back pain, but such care plans are not normally conducted [30] because most physicians lack sufficient time and training to implement recommended procedures [6,26].

An alternative approach is to develop an NLBP intervention that could be widely available online without requiring medical supervision. Although many websites offer education or treatment, none offer self-management interventions for NLBP that have been empirically tested for efficacy. We developed “FitBack”, an online program with responsive design architecture (accessible to computers and mobile devices) [33] that provides a self-management intervention that promotes use and self-monitoring of cognitive and behavioral strategies to improve self-care and back pain prevention behaviors with tailored information and support using gain-framed messaging [34,35].

In the research reported here, we tested FitBack in a randomized design (Clinicaltrials.gov NCT01950091) with a population of adults at increased risk for chronic back pain due to a recent episode of NLBP. We hypothesized that the intervention would improve self-reported outcomes of pain, functionality/quality of life/well-being, engagement in behaviors to help or prevent back pain, work productivity, and that it would be correlated with theoretically relevant psychosocial mediators of behavior change (patient activation, knowledge, attitudes, self-efficacy, behavioral intentions), and that user acceptance would be positive.

## Methods

### Intervention Program

FitBack is a multiple-visit online program that provides adults with NLBP education and behavioral strategies to manage current pain and prevent future pain episodes. The app’s responsive design approach [33] allows users to access the program from multiple devices and screen sizes (mobile phone, tablet, computer). The interactive framework was developed in consultation with a panel of pain professionals with expertise in orthopedic surgery, physical therapy, and pain psychology. These experts also helped develop content, approved scripts, and participated in app reviews during the development of FitBack. Care was taken to recommend only activities that the participants could do safely with minimal equipment while unsupervised.

The intervention uses a self-tailored cognitive-behavioral approach, based on (1) expert panel and American Pain Society (APS) recommendations [1], (2) formative research in this and previous online physical activity studies with sedentary individuals (NCT01579240) [36,37], (3) the theoretical benefits of behavioral control espoused in social cognitive theory (SCT) [38,39], and (4) the Theory of Planned Behavior (TPB) [40,41]. The FitBack user experience is designed to allow users control over the cognitive and behavioral strategies they use to impact their NLBP and to develop and support users’ self-efficacy related to pain management and prevention. Interventions based on TPB have recently been shown to produce large effects on behavior in online interventions [42].

Using a pain and activity self-monitoring tool and gain-framed text and video messages, FitBack helps users develop a self-tailored approach to manage any current NLBP and activate behaviors for prevention of future NLBP. Text articles and videos are segmented to address issues and self-care activities specific to job type: people who sit most of the day (sitters), stand most of the day (standers), drive most of the day (drivers), and do a substantial amount of lifting each day (lifters).

**Figure 1:**
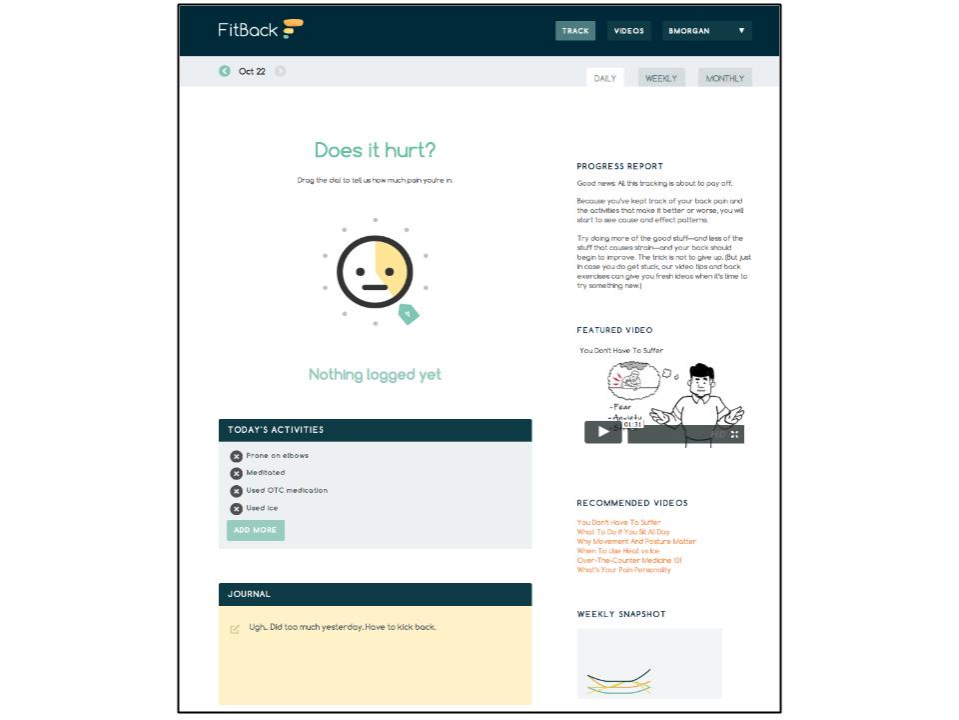
FitBack daily tracking page. Users indicate intensity of current pain (top left), can add current pain prevention activities (middle left), and use the journal tool (bottom left). Users can access featured and recommended videos (right) and charts of activities and pain (snapshot and link bottom right).

The FitBack intervention is designed to encourage users to adopt appropriate pain prevention behaviors, tracking them against self-reported pain level during brief repeat interactions. Users receive weekly emails with gain-framed pain self-care messages and prompts to return to the FitBack program to track pain and self-care activities. At each return visit, users are encouraged to report their current level of back pain using a 10-point “pain dial” (Figure 1) adapted from the Wong Baker pain scale [43]. Users also track their daily pain management activities using an “activity picker” populated with pain self-care activities in four categories (rest and relief, mindfulness, general fitness, and back pain-specific stretching and strength exercises) developed with the panel of pain experts and physicians. The activity picker also allows users to add their own custom activities. A journaling feature prompts users to record notes and experiences related to their pain management efforts. FitBack provides users with simple 7-day and 30-day graphs to identify trends in pain level as associated with each category of self-management activity.

Users have unlimited access to 30 brief (1-4 minute) videos on general aspects of pain and pain management, cognitive and behavioral strategies to manage and prevent pain (eg, controlling fear of pain, mindfulness and relaxation, use of heat and ice, over-the-counter medications, benefits of staying active), and instructional videos on specific strength and stretching exercises tailored by job type (sitter, stander, driver, lifter). Videos used gain-framed messaging delivered by an animated whiteboard-style coach (Figure 1) and behaviorally focused live-action instructional videos on ergonomics and exercises. Messages in the weekly emails, links within the activity picker, and recommendations within the FitBack program repeatedly link users to the video content.

### Research Design

**Figure 2:**
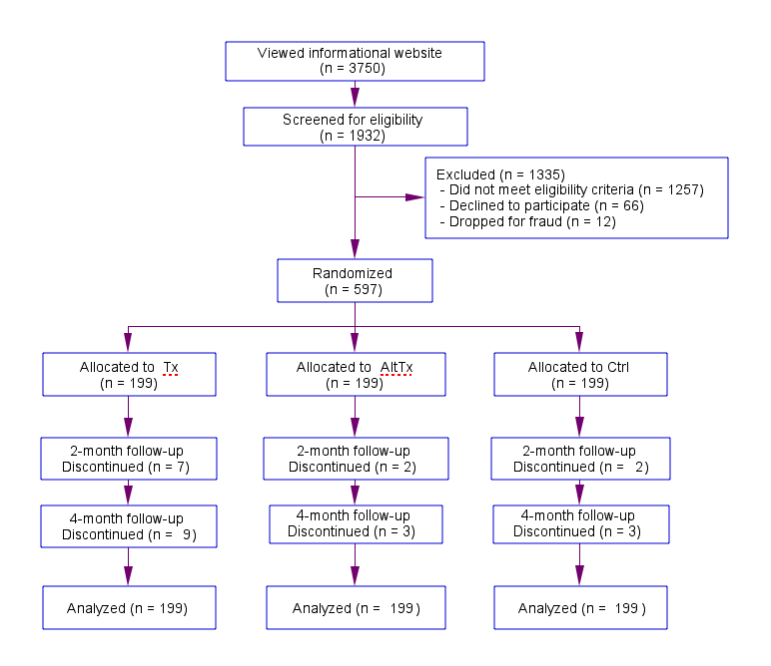
Research design flowchart for FitBack program evaluation.

The study was a 3-arm randomized controlled trial on the Internet with three assessments: pre-test (T1), post-intervention at 8 weeks after pre-test (T2), and post-intervention at 16 weeks after pre-test (T3; see Figure 2). After screening into the study, agreeing to the online informed consent, and submitting the T1 assessment, the total sample of 597 participants was randomized into (1) treatment group (n=199), which used the FitBack intervention, (2) alternative care group (n=199), which received 8 emails with links to 6 websites with information about low back pain, or (3) usual care control group (n=199), which only received emails as requests to complete the assessments.

### Recruitment

#### Overview

After approval by an Institutional Review Board (IRB) for protection of human subjects, the study was conducted entirely on the Internet, with recruitment and assessments hosted by Survey Console, a provider of online survey tools. The study was conducted in partnership with a large health insurer who promoted the project to client companies.

Four companies (trucking, manufacturing, technology, and a corporate headquarters) with a total of approximately 12,000 employees agreed to promote the research project via their preferred in-house communication channels. Some companies relied on flyers and hard copy media, whereas others used the company website, electronic media, and email.

Recruitment efforts were launched simultaneously in all four companies, but after 30 days, fewer than half the desired number of participants had signed up, and visits by potential participants to the informational website (see below) had declined dramatically. Consequently, while recruitment by the four companies continued, we initiated supplemental online recruitment of participants not affiliated with the four companies via online resources (eg, trucker websites, craigslist and other online classified ads, Facebook). We also sent emails to 1200 participants from previous unrelated research projects who had indicated an interest in possible involvement in future projects.

#### Eligibility

Participants recruited through collaborating companies were required to (1) be 18 to 65 years of age living in the United States (because it was a National Institutes of Health Small Business Innovation Research grant), (2) be employed at least half time (which is typical for employees to receive health benefits), retired, or a family member of an employee at one of the four collaborating companies; one participant per family, (3) have experienced low back pain within the past 3 months, (4) not be experiencing back pain so intense it interfered with everyday life, (5) have no history of medical care for back pain or prescription medications for back pain, (6) not be participating in a monitored exercise program for back pain, (7) have a working email address, (8) respond to an online video demonstrating that they had access a computer that could play video on the Internet, and (9) be cleared of medical risks by an online screening survey (see below). When recruitment was expanded to open Internet enrollment, a new parallel online screening process was developed with the same requirements, except that all potential participants were required to report that they were employed at least half time with any employer.

#### Participant Screening

Interested individuals linked to an information website that described the research project and eligibility requirements. If still interested, they linked from there to a 5-15 minute online screening questionnaire to determine eligibility. The online screening questionnaire collected information on demographics, employment status, workplace, and possible medical risk factors.

The screening survey included 40 required questions about back pain history, including current and recent pain (ie, current back pain intensity of 6 or higher; no back pain in past 6 months; continuous back pain for more than 3 months; taking medication for back pain; on a monitored exercise plan for back pain) and health conditions that might contribute to back pain (ie, cancer, infection, fracture risk, cauda equina syndrome, rheumatoid arthritis, numbness in arms or legs, major muscle weakness). These questions were adapted from an instrument developed by the APS [1,32] in consultation with the panel of pain experts and physicians to identify potential study participants whose medical condition might be compromised by participating in the research. Individuals who did not meet the back pain history (n=468), medical (n=706), or other eligibility (n=83) criteria were not accepted to participate (Figure 2).

### Procedures

#### Overview

After submitting the screening survey, eligible individuals were emailed a link to an informed consent. Participants read and agreed to the consent, after which they provided contact information, including email, mailing address, and telephone number. After their data were checked for fraud (see below), participants were emailed a link to the T1 assessment. Personal privacy was protected with a unique user ID and password for each participant.

After submitting the T1 assessment, each participant was emailed his or her experimental group assignment (treatment, alternative care, or control group). Intervention and alternative care group members subsequently received 8 weekly reminder emails to either log on to FitBack (treatment) or access the 6 website links included in the email (alternative care). The automated emails were delivered via Mail Chimp, an online email campaign service provider.

The protocol for prompting intervention participants who failed to submit assessments within 4 days of the first email included up to 4 email reminders at 3-day intervals, followed by a telephone call about 10 days after the fourth email reminder. The call attempted to verify that technical difficulties were not responsible for the lack of participant communication. This protocol was developed based on our experiences in other online studies and was approved by our IRB. We believe that it allowed for conscientious follow-up of participants without undue harassment.

#### Group Assignment

Intervention group members received log-in information and a link to the FitBack intervention website, and were enrolled to receive the 8 program emails with content and prompts related to NLBP self-management (described above). Participants who did not make an initial visit to FitBack within 2 weeks (17/199, 8.5%) of the assignment email were telephoned once by the research staff. The call was framed as a check-in to verify the participants were receiving the emails, and the caller encouraged the recipient (during a telephone interaction or via voicemail) to visit the program. Participants were not contacted further by the research staff, and we did not attempt to determine who clicked to open their reminder messages.

The alternative care group received an initial email and 8 reminder emails, each of which included links to 6 websites about NLBP [44-48]. The websites provided a choice of popular, educational, and medically oriented online resources. We did not attempt to follow up with participants who did not open their emails.

After the initial group-assignment email, control group members were contacted by email only with links to the T2 and T3 assessments.

#### Assessments

Two months after submitting their T1 assessment, participants were emailed a link to the T2 survey. After T2, use of FitBack by treatment group participants and the list of websites for the alternative care group remained available, but reminder emails were discontinued. Two months after T2 (4 months after T1), participants were emailed a link to the T3 assessment. The protocol to encourage submission of T2 and T3 assessments was similar to that used for T1 assessments. All participants were mailed a check after submitting each assessment: US $40 for T1, US $50 for T2, and US $60 for T3. Participants in the treatment and alternative care groups received no financial incentives to use the websites made available to them.

#### Fraudulent Activity

A problem with Internet studies is that researchers are rarely in direct contact with participants. Our previous online studies have found some applicants each time who attempt to screen-in to a study by providing false information (eg, same name or IP address shows inconsistent age, gender, ethnicity, or country) [36,37,49-51]. Consequently, in this study, participant demographic data was checked for fraudulent information against our database of about 20,000 records of previous Internet study applicants, and 12 individuals were dropped (Figure 2). To prevent fraudulent attempts to qualify by subsequently re-taking the screening survey and changing responses, disqualified applicants were not informed about exactly why they had not qualified.

### Measures

#### Physical Outcomes

##### Back Pain

An individual’s back pain is an indicator of physical quality of life [52]. Participants’ current back pain was assessed with a Yes/No item: “Do you have low back pain now?” In addition, a set of back pain measures asked about level of back pain, frequency of back pain, intensity of back pain, and duration of back pain.

##### Functionality, Quality of Life, and Well-Being

###### Functionality and Quality of Life

A 10-item scale, adapted from the Multidimensional Pain Inventory Interference Scale (MPI) [53] and the Interference Scale of the Brief Pain Inventory [54], assessed functionality and quality of life during the past 2 months. Participants were asked how back pain interfered in different areas of their lives (day-to-day activities, mood, and productivity at work). Response options were on a 10-point scale (1=does not interfere, 10=completely interferes). The scale showed good reliability (alpha=.94).

###### Dartmouth CO-OP (Function, Well-Being, and Quality of Life)

The 9-item Dartmouth CO-OP (Dartmouth Primary Care Cooperative Information Project) scale [55] measures different aspects of patient health status, including function (physical endurance, emotional health, role function, and social function), well-being (overall health, change in health, level of pain), and quality of life (overall quality of life and social resources/support). Response options were on a 5-point scale with a higher score indicative of poorer health status for each scale. A total sum score was computed, and the scale showed adequate reliability (alpha=.78).

#### Behavioral Outcome

##### Prevention-Helping Behaviors

Four items were designed for the study to assess how often in the past 2 months participants engaged in behaviors intended to help or prevent back pain (eg, In the last 2 months, how often did you do exercises specifically to prevent recurrence of your back pain?). Response options were on a 5-point scale and a mean score computed with a higher score indicative of more engagement in helping behaviors. The measure showed acceptable reliability (alpha=.76).

#### Worksite Outcomes

##### Worker Productivity

The 4-item Work Limitations Questionnaire (WLQ) [56] was used to assess the degree to which a participant’s back pain interfered with work (eg, In the past 2 weeks, how much of the time did your physical health or emotional problems make it difficult for you to get going easily at the beginning of the workday?). Response options were on a 5-point scale and a mean score computed with a higher score indicative of greater productivity. The scale showed adequate reliability (alpha=.76).

##### Presenteeism

The 6-item Stanford Presenteeism scale [57] was adapted to assess the extent to which workers’ back pain inhibited them from doing their jobs (eg, Despite having my back pain, I was able to finish hard tasks in my work.). Response options were on a 5-point scale and mean score computed with a higher score indicative of more effective work practices. The scale showed adequate reliability (alpha=.77).

#### Other Constructs

##### Patient Activation Measure

The Patient Activation Measure (PAM) is a reliable probabilistic scale that assesses activation of patients to take responsibility for their own health [58,59]. A 10-item scale was adapted from the PAM short form to reflect care for low back pain. Participants were asked about their perceptions of taking responsibility for care for their low back pain. Response options were on a 4-point scale and a mean score computed with a higher score indicative of better functioning. The scale showed good reliability (alpha=.79).

##### Theory of Planned Behavior Constructs

###### Knowledge

A total of 14 items based on teaching points in the program assessed improvement in knowledge about back pain (eg, Fear and worry do not influence the intensity of low back pain. When your back hurts, doing simple back exercises many times a day is the best remedy). Item response options were “true” and “false”. The number of correct items was summed and divided by total number of items to reflect the proportion of items answered correctly.

###### Behavioral Intentions

The TPB suggests that behavioral intentions can predict adoption of new behaviors [40,41]. To assess participant intentions to perform the activities recommended in the program, a 14-item scale was created (eg, The next time you experience back pain, how likely is it that you will take action to use the right amount of activity to help you get better faster?). Response options were on a 7-point scale and a mean score computed with a higher score indicative of more intention to perform the activities. The scale showed good reliability (alpha=.90).

###### Self-Efficacy

The importance of behavioral self-efficacy to engage in recommended behaviors is supported by both social cognitive theory [38,39] and the TPB [40,41]. To assess this construct, a 13-item scale was developed. Participants were asked how confident they were in their ability to use the behaviors recommended in FitBack (eg, How confident are you in your ability to use back exercises to reduce your low back pain?). Response options were on a 7-point scale (1=not at all confident, 7=extremely confident) and a mean score computed with a higher score indicative of greater levels of self-efficacy to use the practices taught in the program. The scale showed good reliability (alpha=.93).

##### Attitudes Toward Pain

Attitudes toward pain complicate perceptions of pain and quality of life [52,60] and are linked by the TPB to self-efficacy and intentions to attempt behavioral remedies [40,41]. A 10-item adaptation of the short version of the Survey of Pain Attitudes (SOPA) [61,62] focused on two of the seven pain domains of the SOPA. The items formed two subscales: a 6-item control scale to assess the extent to which a person believes he or she can control pain, and a 4-item emotion scale to assess the extent to which a person believes his or her emotions affect the experience of pain. Response options were on a 5-point scale and a mean score computed for each scale with a higher score indicative of more positive attitudes. Both the control and emotion scales showed good reliability (alphas=.81 and .95, respectively).

##### Catastrophizing of Pain

Fear of pain might indicate vulnerability or a tendency to catastrophize about a painful problem. A 4-item scale that explains 54% of the variance of the Tampa Scale for Kinesiophobia [63] was adapted to assess the degree to which a participant catastrophizes pain with a focus on back pain (eg, My back pain puts my body at risk for the rest of my life). Items were assessed on a 4-point scale and a mean score computed with a higher score indicative of greater levels of catastrophizing. The scale showed adequate reliability (alpha=.77).

#### Process Outcomes

##### User Satisfaction

Four items were administered at T2 and T3 to both treatment and alternative care group participants for comparison purposes. They included satisfaction with information on back health provided, likelihood of recommending the resources to a friend, value for self-treatment of low back pain, and value for preventing back pain occurrence. Response options were on a 7-point scale with a higher score indicative of greater satisfaction with the program.

##### Website Usability

The System Usability Scale (SUS) is a 10-item survey [64] for assessing the usability of a product, including websites, cell phones, interactive voice response systems, and TV applications [65]. We used it to ascertain participants’ attitudes toward the functionality of the FitBack program. It consists of five positively worded items (eg, I think that I would like to use FitBack frequently) and five negatively worded items (eg, I found FitBack unnecessarily complex) on a 5-point agree-disagree rating scale. When scoring the SUS, the items are rescaled so that when they are summed they range from 0 to 100. An overall sum score was computed with a higher score indicative of more positive attitudes toward the program. The SUS can be scored as a percentile rank and compared with 500 other studies in a process comparable to grading on a curve, with a score of 68 considered average [64,65].

##### Perceptions of Employers

Five items were used to assess how employees would view an employer who made the FitBack program available. The stem was “If my employer made FitBack available to all company employees”, and responses included, “I would feel like my company cares about me”; “I would feel a greater commitment to my company”. Response options were on a 6-point scale with a higher score indicative of a more favorable impression of the employer.

##### Understanding and Implementation Survey

A 7-item survey was designed to ascertain to what degree the participant understood and implemented the teaching points of the FitBack program (eg, Did you understand program recommendations about using heat or ice to help deal with back pain when it occurs?). Categorical response options were “yes”, “yes, somewhat”, “no”, “not much”, and “not at all”.

### Statistical Methods

#### Preliminary Analysis

Chi-square tests and one-way analysis of variance models were used to compare the three groups to determine whether the groups differed at baseline on the demographic characteristics, the outcome measures, and the mediating measures. None of these analyses was found to be statistically significant at *P*<.05, suggesting randomization produced initially equivalent groups.

All 597 participants completed the T1 assessment, 586 (2% attrition across all participants; n=11; 7 treatment, 2 alternative care, 2 control) completed T2, and 582 (3% attrition across all participants; n=15; 9 treatment, 3 alternative care, 3 control) completed T3 (Figure 2). Participants who completed all three assessments (580/597, 97.1%) were compared to those who did not (17/597, 2.8%) on study condition, demographic characteristics, baseline outcomes, and baseline back pain measures. No statistically significant differences were found with the exception of the baseline intention score. Participants who did not complete all three assessments had significantly (*t*_594_=2.22, *P=*.049) lower intention scores than those who did complete all assessments (3.45 vs 4.07, respectively). However, mean differences were associated with a small effect size (Cohen’s *d*=.27) [66].

#### Missing Data

Rates of missing data ranged from 0-5% at T1, 2-5% at T2, and 3-8% at T3. Despite the low rates of missing data, one fully imputed data set was generated for this intent-to-treat analysis as it produces less bias then other missing data techniques, such as list-wise deletion and last observation carried forward [67]. Missing data were imputed using IVEWare [68], which uses all available data to impute missing data via a sequential regression approach. The observed and imputed data were compared to ensure they showed similar distributions [69].

#### Analytic Models

The critical analysis focused on the physical outcome measure of current pain, which ascertained whether or not a study participant was currently experiencing back pain (“yes” or “no” response option). Logistic regression models were used to determine whether study condition predicted current back pain status at T2 and T3, separately, with the T1 response as a covariate.

All other analyses of outcome measures and mediating constructs utilized multivariate analysis of covariance (MANCOVA) models to test for group differences on study outcomes at T2 and T3, separately, with the T1 score as a covariate and study condition as a three-level predictor (1=treatment, 2=alternative care, 3=control). If the overall test was significant, then follow-up planned contrasts (treatment vs alternative care and treatment vs control) were examined. Eta-square is provided as a measure of effect size with the convention .01 small, .06 moderate, and .14 large [66]. Finally, independent *t* tests were used to compare the FitBack participants and alternative care participants on four program satisfaction items administered as part of the T2 and T3 surveys.

## Results

### Participants

Participants were 597 workers recruited from our worksite partner (n=244) and the general work population (n=353). Worker job types and other demographic information are shown in Table 1. About half of the participants (302/597, 50.6%) indicated they currently had low back pain. Chi-square statistics and associated *P* values show that experimental groups did not differ on demographic characteristics. All analyses reported in the analysis section below were also completed with worker recruitment type (ie, worksite partner vs general work population) as a factor in each analysis. These analyses found no significant effect for the interaction of worker recruitment type and condition. That is, there were no differential condition effects across worker recruitment type. Thus, this factor and interaction were dropped from all the analysis models.

Participant-reported pain characteristics for each of the three study conditions are described in Table 2. Chi-square tests were computed to compare groups on all items; only one item (duration of pain) was significantly different (*P*=.04) with the control group and treatment group reporting somewhat higher percentages in two different response categories.

### Analyses

#### Physical Outcomes

##### Current Back Pain

Rates of current back pain were 48%, 54%, and 50% for the treatment, alternative care, and control participants, respectively, at T1 (χ^2^_597_=1.78, *P=*.41); 42%, 46%, and 49% at T2 (χ^2^_597_=2.00, *P=*.37); and 29%, 41%, and 41% at T3 (χ^2^_597_=7.61, *P=*.02). Two contrasts were created: treatment (=0) vs control (=1) and treatment (=0) vs alternative care (=1). Logistic regression models were run with the contrasts as the outcomes, with T2 and T3 current back pain scores as predictors, while controlling for T1 current back pain score. Current adjusted back pain status at T2 was not a statistically significant predictor of either contrast. At T3, however, current adjusted back pain status was a significant predictor for both the treatment vs control (OR 1.72, 95% CI 1.11-2.68, *P=*.02) and treatment vs alternative care (OR 1.60, 95% CI 1.03-2.50, *P=*.035) contrasts. Subjects in the alternative care group were 1.6 times more likely to report current back pain than subjects in the FitBack treatment group and subjects in the control group were 1.7 times more likely to report current back pain than subjects in the FitBack treatment group.

##### Back Pain Measures

Table 3 provides means and standard deviations for all other outcome measures and the other constructs at each time point across all three study conditions, and Table 4 provides the results of the MANCOVA models testing for group differences at the T2 and T3 assessments, including effect size measurements. The overall *F* for the back pain measures was significant at T3, but not at T2. The treatment vs control follow-up comparison was statistically significant at T3, but not at T2.

##### Functionality, Quality of Life, and Well-Being

For this physical outcome measure, the overall tests were significant at both T2 and T3. In addition, the treatment vs control follow-up comparison was statistically significant at both T2 and T3.

**Table 1:** Study demographic characteristics.

Characteristic		Treatment n=199		Alternative care n=199		Control n=199		χ^2^ (df)	*P*
		n	%	n	%	n	%		
Female		116	58.3	117	58.8	125	62.8	1.02 (2,597)	.600
Hispanic/Latino		20	10.1	24	12.1	23	11.6	0.44 (2,597)	.804
**Race**								10.31 (10,597)	.413
	American Indian/Alaskan Native	2	1.0	1	0.5	1	0.5		
	Asian	14	7.0	10	5.0	11	5.5		
	Black or African American	16	8.0	17	8.5	10	5.0		
	White or Caucasian	152	76.4	158	79.4	163	81.9		
	Multiracial	12	6.0	6	3.0	5	2.5		
	Other/unknown	3	1.5	7	3.5	9	4.5		
**Marital status**								11.82 (8,597)	.159
	Married or living with a partner	126	63.3	139	69.8	144	72.4		
	Divorced	20	10.1	15	7.5	23	11.6		
	Widowed	1	0.5	2	1.0	0	0.0		
	Separated	7	3.5	3	1.5	6	3.0		
	Single	45	22.6	40	20.1	26	13.1		
**Highest level of education**								8.77 (8,597)	.362
	Less than high school graduate	2	1.0	0	0.0	2	1.0		
	High school graduate/GED	24	12.1	16	8.0	13	6.5		
	Some college	57	28.6	64	32.0	75	37.7		
	College degree	75	37.7	78	39.2	76	36.7		
	Graduate school	41	20.6	41	20.6	36	18.1		
**Annual household income (USD)**								11.70 (10,597)	.306
	Less than $20,000	10	5.0	9	4.5	13	6.5		
	$20,000 - $39,999	38	19.1	28	14.1	36	18.1		
	$40,000 - $59,999	45	22.6	38	19.1	52	26.1		
	$60,000 - $79,999	37	18.6	34	17.1	24	12.1		
	$80,000 - $99,999	26	13.1	29	14.6	29	14.6		
	More than $100,000	43	21.6	61	30.7	45	22.6		
**Employment status**								12.75 (12,597)	.388
	Full time	144	72.4	143	71.9	143	71.9		
	Part time	28	14.1	31	15.6	30	15.1		
	Retired	3	1.5	1	0.5	0	0.0		
	Volunteer	3	1.5	0	0.0	2	1.0		
	Homemaker	15	7.5	17	8.5	17	8.5		
	Unemployed	1	0.5	6	3.0	4	2.0		
	Non-working student	5	2.5	1	0.5	3	1.5		
**Worker classification**								1.82 (6,597)	.936
	Sitter	124	62.3	124	62.3	124	62.3		
	Stander	50	25.1	51	25.6	49	24.6		
	Lifter	22	11.1	23	11.6	22	11.1		
	Driver	3	1.5	1	0.5	4	2.0		

**Table 2:** History of back pain.

History		Treatment n=199		Alternative care n=199		Control n=199		χ^2^ (df)	*P*
		n	%	n	%	n	%		
**Do you have back pain now?**								1.78 (2,597)	.410
	Yes	95	47.7	108	54.3	99	49.7		
	No	104	52.3	91	45.7	100	50.3		
**How bad is your current back pain?**								10.05 (10,302)	.435
	Mild and comes and goes	45	47.9	43	39.8	40	40.0		
	Mild and does not vary much	11	11.7	14	13.0	13	13.0		
	Moderate and comes and goes	33	35.1	40	37.0	36	36.0		
	Moderate and does not vary much	4	4.3	5	4.6	10	10.0		
	Severe and comes and goes	1	1.1	5	4.6	1	1.0		
	Severe and does not vary much	0	0.0	1	0.9	0	0.0		
**In the last two months have you experienced back pain?**								3.57 (8,594)	.964
	Rarely	11	5.5	13	6.5	14	7.0		
	Once in a while	59	29.6	48	24.1	47	23.6		
	Sometimes	73	36.7	76	38.2	80	40.2		
	Often	54	27.1	58	29.1	55	27.6		
	Always	1	0.5	3	1.5	2	1.0		
	No pain, does not apply to me	1	0.5	1	0.5	1	0.5		
**When you experienced back pain in the last 2 months, how intense was the pain?**								14.78 (12,594)	.253
	Mild; it came and went	45	22.7	44	22.2	35	17.7		
	Mild and it did not vary much	35	17.7	28	14.1	26	13.1		
	Moderate; it came and went	81	40.9	95	48.0	85	42.9		
	Moderate and it did not vary much	31	15.7	20	10.1	39	19.7		
	Severe; it came and went	4	2.0	8	4.0	10	5.1		
	Severe and it did not vary much	1	0.5	2	1.0	3	1.5		
	Severe as anything I can imagine	1	0.5	1	0.5	0	0.0		
**When you experienced low back pain in the last 2 months, how long did it usually last?**								13.01 (6,590)	.043
	Up to 30 minutes	36	18.4	26	13.3	23	11.6		
	30 minutes to 6 hours	57	29.1	79	40.3	78	39.4		
	6 to 24 hours	63	32.1	47	24.0	45	22.7		
	Days to weeks at a time	40	20.4	41	20.9	52	26.3		
	For a month or more	0	0.0	3	1.5	0	0.0		
**When you experienced back pain, what do you think is the primary cause of it?**								19.08 (16,597)	.264
	Recurrence of a previous pain event	18	9.0	11	5.5	20	10.1		
	Overuse	18	9.0	20	10.1	17	8.5		
	Lifting or straining	38	19.1	36	18.1	36	18.1		
	Twisting	7	3.5	4	2.0	8	4.0		
	Abrupt movement	3	1.5	7	3.5	10	5.0		
	Sitting or standing for too long	77	38.7	71	35.7	56	28.1		
	Unusual physical activity	9	4.0	8	4.0	3	1.5		
	Minor injury or accident	1	0.5	2	1.0	3	1.5		
	Don’t know	29	14.6	40	20.1	46	23.1		

**Table 3:** Descriptive statistics for study outcomes by study group.

		T1^a^		T2^b^		T3^c^	
		mean	SD	mean	SD	mean	SD
							
**How bad is your low back pain?**							
	Treatment	0.96	1.26	0.82	1.22	0.56	1.00
	Alternative care	1.22	1.43	1.03	1.43	0.89	1.30
	Control	1.09	1.34	1.16	1.47	0.98	1.43
**How often have you experienced low back pain?**							
	Treatment	2.86	0.92	2.64	1.04	2.16	1.12
	Alternative care	2.93	0.95	2.63	1.02	2.39	1.05
	Control	2.90	0.94	2.76	0.95	2.52	1.06
**When you experienced low back pain, on average how intense was the pain?**							
	Treatment	2.59	1.15	2.23	1.20	2.11	1.46
	Alternative care	2.63	1.17	2.26	1.24	2.23	1.30
	Control	2.84	1.18	2.52	1.29	2.55	1.41
**When you experienced low back pain, on average how long did it usually last?**							
	Treatment	2.52	1.03	2.28	1.05	2.03	1.01
	Alternative care	2.56	1.02	2.25	1.03	2.16	0.97
	Control	2.62	1.01	2.36	1.03	2.28	1.04
**Functionality and quality of life**							
	Treatment	3.83	1.90	3.27	1.69	3.03	1.88
	Alternative care	3.93	1.97	3.45	1.90	3.31	2.00
	Control	4.03	2.00	3.85	2.22	3.74	2.22
**Dartmouth CO-OP**							
	Treatment	20.41	5.02	19.30	5.18	18.84	5.39
	Alternative care	20.66	4.74	19.87	5.16	19.42	5.26
	Control	21.01	4.96	20.84	5.92	20.65	5.64
**Prevention-helping behaviors**							
	Treatment	2.48	0.84	3.09	0.84	3.18	0.91
	Alternative care	2.47	0.81	2.88	0.82	2.97	0.87
	Control	2.48	0.79	2.64	0.85	2.74	0.89
**Work productivity**							
	Treatment	4.09	0.74	4.14	0.68	4.26	0.72
	Alternative care	4.09	0.65	4.16	0.69	4.23	0.75
	Control	4.08	0.72	4.08	0.78	4.14	0.74
**Presenteeism**							
	Treatment	3.88	0.79	4.05	0.79	4.15	0.74
	Alternative care	3.10	0.47	4.02	0.77	4.01	0.82
	Control	3.75	0.79	3.86	0.81	3.92	0.81
**Patient activation**							
	Treatment	3.14	0.45	3.38	0.50	3.51	0.50
	Alternative care	3.10	0.47	3.34	0.51	3.37	0.53
	Control	3.09	0.46	3.11	0.53	3.14	0.53
**Knowledge**							
	Treatment	0.50	0.16	0.59	0.13	0.61	0.14
	Alternative care	0.47	0.17	0.55	0.16	0.54	0.15
	Control	0.47	0.16	0.50	0.16	0.51	0.16
**Behavioral intentions**							
	Treatment	4.02	1.08	4.59	1.07	4.90	1.15
	Alternative care	4.07	1.14	4.48	1.16	4.65	1.26
	Control	4.08	1.18	4.03	1.12	4.12	1.20
**Self-efficacy**							
	Treatment	3.80	1.13	4.54	1.15	4.98	1.23
	Alternative care	3.76	1.19	4.44	1.26	4.67	1.24
	Control	3.75	0.79	3.87	1.26	3.99	1.31
**Survey of pain attitudes: control scale**							
	Treatment	3.19	0.87	3.69	0.79	3.86	0.79
	Alternative care	3.31	0.89	3.56	0.95	3.70	0.89
	Control	3.23	0.87	3.31	0.95	3.37	0.94
**Survey of pain attitudes: emotions scale**							
	Treatment	2.96	1.28	3.32	1.28	3.38	1.22
	Alternative care	3.01	1.32	3.30	1.27	3.47	1.29
	Control	2.92	1.30	3.08	1.28	3.28	1.35
**Catastrophizing of pain**							
	Treatment	2.25	0.58	2.26	0.57	2.25	0.64
	Alternative care	2.23	0.61	2.22	0.62	2.22	0.67
	Control	2.22	0.59	2.30	0.65	2.33	0.71

^a^T1: pre-test

^b^T2: post-intervention at 8 weeks after pre-test

^c^T3: post-intervention at 16 weeks after pre-test

**Table 4:** Results of multivariate/univariate analysis of covariance models testing for group differences^a^ at T2^b^ and T3^c^.

		Overall test			Specific group comparisons					
					Treatment vs alternative care			Treatment vs control		
		*F*	*P*	η^2d^	*F*	*P*	η^2^	*F*	*P*	η^2^
**Back pain**										
	T2	1.25	.266	.008	N/A^e^	N/A	N/A	N/A	N/A	N/A
	T3	2.54	.010	.017	1.62	.169	.016	4.41	.002	.043
**Functionality, quality of life, and well-being**										
	T2	3.19	.013	.011	0.70	.496	.004	5.88	.003	.029
	T3	3.69	.005	.012	0.97	.377	.005	6.76	.001	.033
**Prevention-helping behaviors**										
	T2	23.60	.026	.07	9.32	.017	.02	33.83	.017	.08
	T3	17.61	.013	.06	6.88	.025	.02	46.81	.009	.11
**Worksite outcomes**										
	T2	1.14	.338	.004	N/A	N/A	N/A	N/A	N/A	N/A
	T3	2.42	.047	.008	3.36	.036	.07	3.65	.027	.018
**Patient activation**										
	T2	18.61	.004	.06	0.16	.687	<.01	28.75	.003	.07
	T3	27.20	.004	.08	7.08	.027	.02	54.83	.002	.12
**Theory of planned behavior**										
	T2	11.90	<.001	.057	2.14	.095	.016	22.74	<.001	.149
	T3	17.21	<.001	.080	7.10	<.001	.052	33.04	<.001	.202
**Attitudes toward pain**										
	T2	6.76	<.001	.022	3.62	.027	.018	13.46	<.001	.064
	T3	11.02	<.001	.036	4.95	.008	.026	22.39	<.001	.102
**Catastrophizing of pain**										
	T2	1.92	.174	<.01	N/A	N/A	N/A	N/A	N/A	N/A
	T3	2.99	.069	.01	N/A	N/A	N/A	N/A	N/A	N/A

^a^Results from prevention-helping behaviors, patient activation, and catastrophizing of pain are from analysis of covariance models; all other results from multivariate analysis of covariance models.

^b^T2: post-intervention at 8 weeks after pre-test

^c^T3: post-intervention at 16 weeks after pre-test

^d^η^2^=eta-square: measure of effect size with convention .01 small, .06 medium, large .14.

^e^N/A: not applicable; test not run because overall test not statistically significant.

#### Behavioral Outcome

For the Prevention-Helping behavioral measure, which assessed the level of engagement in behaviors intended to help or prevent back pain, the overall tests were significant at both T2 and T3. Both the treatment vs control comparison and the treatment vs alternative care comparison were statistically significant at both T2 and T3.

#### Worksite Outcomes

Regarding the Worker Productivity and the Presenteeism measures, the overall tests were significant at T3, but not at T2. Similarly, both the treatment vs control and treatment vs alternative care comparisons were significant at T3, but not T2.

#### Other Constructs

##### Patient Activation

The analyses of the Patient Activation Measure, which assessed the activation of patients to take responsibility for care for their own low back pain, found that the overall tests were significant at both T2 and T3. The treatment vs control follow-up comparisons were statistically significant at both T2 and T3. The treatment vs alternative care comparisons were statistically significant at T3, but not at T2.

##### Theory of Planned Behavior Constructs

The overall tests were significant at both T2 and T3. Both the treatment vs control and the treatment vs alternative care comparisons were statistically significant at T3, but only the treatment vs control comparison was significant at T2.

##### Attitudes Toward Pain

The overall tests were significant at both T2 and T3. Both the treatment vs control and the treatment vs alternative care comparisons were statistically significant at T2 and T3.

##### Catastrophizing of Pain

The overall tests for the Catastrophizing of Pain scale were not significant at either T2 or T3.

#### Process Analyses

##### User Satisfaction

Indices of user acceptance were all positive. Compared to the alternative care participants, FitBack program users had higher satisfaction ratings. The mean total score for FitBack participants was statistically greater at T2 (*t*_380_=4.40, *P<*.001, *d*=.54) and T3 (*t*_382_=3.51, *P<*.001, *d*=.37).

##### Website Usability

The System Usability Scale (SUS) score (mean 78.6, SD 15.7), when compared to normative data, is associated with “good” to “excellent” ratings and corresponds to a “B-” [65]. For comparison, across 3500 surveys within 273 studies on different platforms (Web, mobile phones, TV, etc), the average SUS score was approximately 70. For Web applications, the average SUS score was 68.2 [65].

##### Perception of Employer Survey

The summary analyses suggest that participants believed they would have a positive impression of employers who made the FitBack program available to employees. They felt that the company would care about them (mean 4.7, SD 1.1); they would feel more positive about the company (mean 4.6, SD 1.1); they would have greater commitment to the company (mean 4.1, SD 1.2); they would be more productive (mean 4.1, SD 1.2); and they would feel more job satisfaction (mean 4.0, SD 1.3).

##### Understanding and Implementation Survey

The results indicate that 96-98% of participants thought they understood the program recommendations for use of heat and ice, over-the-counter medications, exercising to deal with back pain, and relaxation techniques. Implementation of recommendations by participants was reported for relaxation activities (67.8%), exercises for prevention (78.2%), and dealing with pain occurrences (86%).

## Discussion

### Physical and Behavioral Outcomes

The major findings of the study were related to critical physical and behavioral outcomes. Users of the FitBack program were (1) significantly less likely to be experiencing current back pain at 4-month follow-up than either control (OR 1.7) or alternative care (OR 1.6) subjects, (2) significantly less likely to be experiencing back pain generally (ie, level, frequency, intensity, and duration of back pain) at 4-month follow-up than control subjects, (3) significantly more likely to have better functionality, quality of life, and well-being at both 2- and 4-month follow-up than control subjects, and (4) significantly more likely to be engaging in behaviors intended to help or prevent back pain at both 2- and 4-month follow-up than either control or alternative care subjects. In sum, the FitBack program’s positive effects on physical and behavioral outcomes were consistent at 4-month follow-up comparisons with control subjects, and with 2 of 4 outcomes at 2-month follow-up. Given that the alternative care group received an intervention designed to prompt the use of 6 website links via 8 email reminders, it is not surprising that for some measures the FitBack effects were not significantly larger than the alternative care intervention. Nonetheless, alternative care users were 1.6 times more likely than the FitBack program users to be experiencing current back pain at 4-month follow-up and were significantly less engaged in prevention-helping behaviors at both 2- and 4-month follow-ups.

### Worksite Outcomes

The above improvements in Physical and Behavior Outcomes translated to significant improvement in worker productivity and presenteeism at 4-month follow-up, but not at 2-month follow-up. It may take a longer time period for physical and behavioral changes to be detectable in worksite outcome measures. These worksite outcomes are central to making the case for the cost-effectiveness of online interventions such as FitBack.

### Other Constructs

#### Patient Activation

This study is notable because it establishes in a randomized controlled trial that an online intervention that is designed to help users develop self-tailored strategies to treat NLBP occurrences and adopt behaviors to decrease future pain occurrences can improve the level of patient activation (ie, patients’ taking responsibility for care for their low back pain) at 4-month follow-up.

#### Theory of Planned Behavior

This study indicated that an online intervention can effectively improve measures of constructs central to the TPB, specifically user knowledge, behavioral intentions, and self-efficacy.

#### Attitudes Toward Pain

##### Overview

Attitudes toward pain are connected by the TPB to self-efficacy and intentions to attempt behavioral remedies [40,41]. This study demonstrated that the FitBack program consistently improved both the extent to which a person believes he or she can control pain, and the extent to which a person believes his or her emotions affect the experience of pain.

##### Catastrophizing of Pain

The FitBack program did not affect the degree to which the user catastrophizes about back pain.

#### Process Outcomes

The measures of user satisfaction were positive. In addition, the FitBack program users had higher satisfaction ratings than alternative care participants. The FitBack program received “good” to “excellent” usability ratings. Last, FitBack users reported that they would have a positive impression of employers that made the FitBack program available to employees.

### Online Recruitment

The research reported here adds to the literature on recruitment success of online research studies. A total of 3570 views of the informational website (Figure 2), led to 1932 respondents who submitted the online screening questionnaire (78.4%), which is substantially more than the 17.3% who submitted the online screening for an exercise study with sedentary older adults [37]. Also of potential interest to other researchers is the incidence of fraud reported here. Of 675 individuals who initially screened in as eligible, 12 (1.8%) were dropped because of fraudulent information, which is much less than the 9% dropped for fraud by Irvine and colleagues [37]. Across all our online studies, we have identified roughly 4% of those who initially qualify to be fraudulent because they supply inaccurate personal information to be accepted as a research subject. We believe that the potential for fraudulent participation in Internet research studies is an important issue, but few researchers report on it.

### Limitations

The results reported here must be viewed cautiously because we believe this to be the first attempt to influence NLBP with an online intervention. We cannot gauge the importance of the email reminders on the results, which potentially could influence the response rate [70], and we only prompted the treatment group if they did not open the first message, which might have biased the response rate. Additionally, we cannot verify that participants provided accurate information on eligibility criteria, the surveys, and the 4-month follow-up period was somewhat limited. Perhaps 1-2-year follow-up studies, possibly combined with medical verification, would provide greater confidence in the intervention effects, as would research to tease out which aspects of FitBack were most effective. Also, we cannot determine whether social desirability bias might have influenced responses to assessment items, as has been reported elsewhere [71].

Research is needed to determine whether the results presented here generalize to other demographic categories. Participants tended to be employed, educated, with at least a middle-class income. Less educated, lower income, and rural populations might be less likely to have Internet in their homes [72], and FitBack would obviously be inapplicable for those who do not use computers or mobile phones.

### Conclusions

The major conclusion of this study is that users of the FitBack program showed greater improvement compared to the control group in every comparison of the critical physical, behavioral, and worksite outcome measures at 4-month follow-up. In addition, the users of the FitBack program performed better than the alternative treatment group on presence of current back pain, behavioral, and worksite outcomes at 4-month follow-up. Further, the users of the FitBack program showed greater improvement compared to both the control group and the alternative treatment group at 4-month follow-up on patient activation, constructs of the Theory of Planned Behavior, and attitudes toward pain.

This research demonstrates that a theoretically based standalone responsive mobile-Web intervention that tailors content to users’ preferences and interests can be an effective tool in self-management of low back pain. The results are promising considering that the FitBack intervention was neither supported by professional caregivers nor integrated within a larger health promotion campaign, which might have provided additional support and encouragement for the participants. When viewed from the RE-AIM perspective (ie, reach, efficacy/effectiveness, adoption, implementation fidelity, and maintenance) [73], one of the primary advantages of this type of self-guided intervention is its ability to increase reach at a low cost. This study supports the notion that there is considerable potential value in FitBack as a cost-effective tool that can reach large numbers of people. Still, more research is needed on how self-guided, mobile-Web interventions will be used over time and to understand factors associated with continuing user engagement.

## Multimedia Appendix 1

CONSORT-EHEALTH checklist V1.6.2 [74].

## Abbreviations

ACP: American College of Physicians
APS: American Pain Society
IRB: Institutional Review Board for protection of human subjects
MANCOVA: multivariate analysis of covariance
NLBP: nonspecific low back pain
PAM: Patient Activation Measure
SOPA: Survey of Pain Attitudes
SUS: System Usability Scale
TPB: Theory of Planned Behavior
WLQ: Work Limitations Questionnaire
