# Supplementary material for: Mobile-Web App to Self-Manage Low Back Pain: Randomized Controlled Trial
Source: J Med Internet Res. 2015 Jan 2;17(1):e1. doi: 10.2196/jmir.3130 (PMC4296097; doi:10.2196/jmir.3130)
Supplement: Supplementary file 1 [file jmir_v17i1e1_app1.pdf]

CONSORT-EHEALTH Checklist V1.6.2 Report

(based on CONSORT-EHEALTH V1.6), available at [http://tinyurl.com/consort-ehealth-v1-6].

3130

Date completed

11/24/2013 18:25:38

by

Blair Irvine

Randomized Controlled Trial of Mobile-Web App To Self-Manage Low Back Pain

TITLE

1a-i) Identify the mode of delivery in the title

Randomized Controlled Trial of Mobile-Web App To Self-Manage Low Back Pain

1a-ii) Non-web-based components or important co-interventions in title

NA

1a-iii) Primary condition or target group in the title

Randomized Controlled Trial of Mobile-Web App To Self-Manage Low Back Pain

ABSTRACT

1b-i) Key features/functionalities/components of the intervention and comparator in the METHODS section of the ABSTRACT

Background: Nonspecific low back pain (NLBP) is the diagnosis for individuals feeling back pain that has no underlying medical cause (e.g., tumor, infection, fracture, herniated disc, spinal stenosis). The American College of Physicians (ACP) and the American Pain Society (APS) recommend multidisciplinary treatments for NLBP that lasts more than four weeks. This approach, however, is impractical for many physicians to implement during office visits, and relatively few providers offer NLBP treatment that follows ACP guidelines.
Objectives: This study evaluated the efficacy of an on-line intervention called FitBack, which used a responsive design architecture on computers and mobile devices to help users develop self-tailored strategies designed to treat NLBP occurrences and adopt behaviors to decrease future pain occurrences.
Methods: A total of 597 adults were recruited, screened, consented, and assessed online at baseline, at two months (T2), and at four months (T3). After baseline assessments, participants were randomized into three groups: FitBack intervention, alternative care group that received a list of Internet resources for NLBP, and control. The FitBack and alternative care groups received weekly email reminder prompts for 8 weeks plus emails to do assessments, while the usual care group was only contacted to do assessments.

1b-ii) Level of human involvement in the METHODS section of the ABSTRACT

Background: Nonspecific low back pain (NLBP) is the diagnosis for individuals feeling back pain that has no underlying medical cause (e.g., tumor, infection, fracture, herniated disc, spinal stenosis). The American College of Physicians (ACP) and the American Pain Society (APS) recommend multidisciplinary treatments for NLBP that lasts more than four weeks. This approach, however, is impractical for many physicians to implement during office visits, and relatively few providers offer NLBP treatment that follows ACP guidelines.
Objectives: This study evaluated the efficacy of an on-line intervention called FitBack, which used a responsive design architecture on computers and mobile devices to help users develop self-tailored strategies designed to treat NLBP occurrences and adopt behaviors to decrease future pain occurrences.
Methods: A total of 597 adults were recruited, screened, consented, and assessed online at baseline, at two months (T2), and at four months (T3). After baseline assessments, participants were randomized into three groups: FitBack intervention, alternative care group that received a list of Internet resources for NLBP, and control. The FitBack and alternative care groups received weekly email reminder prompts for 8 weeks plus emails to do assessments, while the usual care group was only contacted to do assessments.

1b-iii) Open vs. closed, web-based (self-assessment) vs. face-to-face assessments in the METHODS section of the ABSTRACT

Background: Nonspecific low back pain (NLBP) is the diagnosis for individuals feeling back pain that has no underlying medical cause (e.g., tumor, infection, fracture, herniated disc, spinal stenosis). The American College of Physicians (ACP) and the American Pain Society (APS) recommend multidisciplinary treatments for NLBP that lasts more than four weeks. This approach, however, is impractical for many physicians to implement during office visits, and relatively few providers offer NLBP treatment that follows ACP guidelines.
Objectives: This study evaluated the efficacy of an on-line intervention called FitBack, which used a responsive design architecture on computers and mobile devices to help users develop self-tailored strategies designed to treat NLBP occurrences and adopt behaviors to decrease future pain occurrences.
Methods: A total of 597 adults were recruited, screened, consented, and assessed online at baseline, at two months (T2), and at four months (T3). After baseline assessments, participants were randomized into three groups: FitBack intervention, alternative care group that received a list of Internet resources for NLBP, and control. The FitBack and alternative care groups received weekly email reminder prompts for 8 weeks plus emails to do assessments, while the usual care group was only contacted to do assessments.

1b-iv) RESULTS section in abstract must contain use data

Results: In all comparisons, the FitBack group showed favorable mean outcome scores relative to either the alternative care or control groups. Seven of the twelve omnibus tests at T2 and eight of the twelve omnibus tests at T3 were statistically significant and warranted follow-up comparisons. All FitBack vs. usual care control follow-up comparisons were statistically significant at T2 and at T3. For FitBack vs. alternative care group, three of seven T2 comparisons were statistically significant with small effect sizes, but seven of the eight comparisons at T3 were statistically significant with small effects sizes. Study participants reporting current back pain at T3 were approximately 1.6 times more likely to be in the alternative care group and 1.7 times more likely to be in the control group than in the FitBack group

1b-v) CONCLUSIONS/DISCUSSION in abstract for negative trials

NA

INTRODUCTION

2a-i) Problem and the type of system/solution

Introduction
Nonspecific low back pain (NLBP), defined here as temporary back pain with no medical signs of a serious underlying condition (e.g., cancer, infection, fracture spinal stenosis) [27], is a pervasive and expensive public health problem in the United States[45-47], where four out of five adults experience it at some point in their lives [1,2]. Back pain is the leading cause of work-related disability and one of the most frequent reasons patients visit a doctor [3, 4]. Costs incurred by U.S. back pain suffers are staggering, estimated at $90.7 billion5 and growing.14, 45 People with back pain spend 60% more on health care than those without back pain.9, 14 Most people with low back pain do not visit a physician50, 51 because episodes of NLBP resolve spontaneously (Savigny et al., 2009), but of those who do see a doctor, 30% experience pain and disability a year later,40 and few return to normal activities (Savigny et al., 2009).
Businesses lose 100 million work days per year,9 with back pain accounting for 5.5% of all productivity loss in the United States — about $2,200 per employee per year.6, 7 Even employees with minor back pain lose 4.6 hours of productivity a week due to decreased performance on the job.10, 11 Beyond its economic toll, NLBP causes significant physical and psychological suffering.48, 49
Although no consensus has been reached about the best treatment for NLBP, multidisciplinary approaches reduce employee sick leave52-54 and are cost effective.41, 55 However, recommended NLBP treatments often involve specialized clinics, which are costly and not widely available, 2, 41-43 and insurance companies often don’t cover multidisciplinary treatments.40, 56 The Joint Clinical Practice Guidelines from the American College of Physicians and American Pain Society27, 57 recommend inclusion of psychosocial assessments and treatments for back pain, but such assessments are not normally conducted 42 because most physicians lack sufficient time and training to implement recommended procedures. 2, 41 This situation is problematic, however, because early management of NLBP is the best approach to prevent chronic back pain (Savigny et al., 2009). Nonetheless, intervention research on non-medical populations of back pain sufferers is rare.44
An alternative approach is to develop an NLBP intervention that could be widely available on the Internet without requiring medical supervision. A Google search for “backache” had 4,160,000 results and “non-specific low back pain,” had 1,380,000 results, indicating that the Web is a rich source of information for individuals with NSLBP. In a superficial search of these results, however, we found that although many sites offered education or treatment, none offered multidisciplinary behaviorally focused on-line interventions for NLBP, much less any that have been empirically tested for efficacy. Consequently, we developed an on-line program with responsive design architecture (accessible to computers and mobile devices; Adobe 2013) called FitBack, which provided tailored information and support with gain-framed messaging [Latimer A, Salovey P, Rothman A 2007; Rothman AJ, Salovey P 1999]. It was designed for individuals with NLBP to improve their self treatment and pain prevention behaviors.
In the research reported here, we tested FitBack in a randomized design (Clinicaltrials.gov NCT01950091). We hypothesized that the intervention would improve self reported outcomes of functionality and quality of life, that it would be correlated with theoretically relevant psychosocial mediators of behavior change (knowledge, attitudes, self efficacy, behavioral intentions), and that user acceptance would be positive. This was a “real-world” effectiveness trial [125; Flay & Sobel 1983] in the participants’ setting of choice, as opposed to an efficacy trial in a more controlled laboratory setting.

2a-ii) Scientific background, rationale: What is known about the (type of) system

Introduction
Nonspecific low back pain (NLBP), defined here as temporary back pain with no medical signs of a serious underlying condition (e.g., cancer, infection, fracture spinal stenosis) [27], is a pervasive and expensive public health problem in the United States[45-47], where four out of five adults experience it at some point in their lives [1,2]. Back pain is the leading cause of work-related disability and one of the most frequent reasons patients visit a doctor [3, 4]. Costs incurred by U.S. back pain suffers are staggering, estimated at $90.7 billion5 and growing.14, 45 People with back pain spend 60% more on health care than those without back pain.9, 14 Most people with low back pain do not visit a physician50, 51 because episodes of NLBP resolve spontaneously (Savigny et al., 2009), but of those who do see a doctor, 30% experience pain and disability a year later,40 and few return to normal activities (Savigny et al., 2009).
Businesses lose 100 million work days per year,9 with back pain accounting for 5.5% of all productivity loss in the United States — about $2,200 per employee per year.6, 7 Even employees with minor back pain lose 4.6 hours of productivity a week due to decreased performance on the job.10, 11 Beyond its economic toll, NLBP causes significant physical and psychological suffering.48, 49
Although no consensus has been reached about the best treatment for NLBP, multidisciplinary approaches reduce employee sick leave52-54 and are cost effective.41, 55 However, recommended NLBP treatments often involve specialized clinics, which are costly and not widely available, 2, 41-43 and insurance companies often don’t cover multidisciplinary treatments.40, 56 The Joint Clinical Practice Guidelines from the American College of Physicians and American Pain Society27, 57 recommend inclusion of psychosocial assessments and treatments for back pain, but such assessments are not normally conducted 42 because most physicians lack sufficient time and training to implement recommended procedures. 2, 41 This situation is problematic, however, because early management of NLBP is the best approach to prevent chronic back pain (Savigny et al., 2009). Nonetheless, intervention research on non-medical populations of back pain sufferers is rare.44
An alternative approach is to develop an NLBP intervention that could be widely available on the Internet without requiring medical supervision. A Google search for “backache” had 4,160,000 results and “non-specific low back pain,” had 1,380,000 results, indicating that the Web is a rich source of information for individuals with NSLBP. In a superficial search of these results, however, we found that although many sites offered education or treatment, none offered multidisciplinary behaviorally focused on-line interventions for NLBP, much less any that have been empirically tested for efficacy. Consequently, we developed an on-line program with responsive design architecture (accessible to computers and mobile devices; Adobe 2013) called FitBack, which provided tailored information and support with gain-framed messaging [Latimer A, Salovey P, Rothman A 2007; Rothman AJ, Salovey P 1999]. It was designed for individuals with NLBP to improve their self treatment and pain prevention behaviors.
In the research reported here, we tested FitBack in a randomized design (Clinicaltrials.gov NCT01950091). We hypothesized that the intervention would improve self reported outcomes of functionality and quality of life, that it would be correlated with theoretically relevant psychosocial mediators of behavior change (knowledge, attitudes, self efficacy, behavioral intentions), and that user acceptance would be positive. This was a “real-world” effectiveness trial [125; Flay & Sobel 1983] in the participants’ setting of choice, as opposed to an efficacy trial in a more controlled laboratory setting.

METHODS

3a) CONSORT: Description of trial design (such as parallel, factorial) including allocation ratio

Introduction

Nonspecific low back pain (NLBP), defined here as temporary back pain with no medical signs of a serious underlying condition (e.g., cancer, infection, fracture spinal stenosis) [27], is a pervasive and expensive public health problem in the United States[45-47], where four out of five adults experience it at some point in their lives [1,2]. Back pain is the leading cause of work-related disability and one of the most frequent reasons patients visit a doctor [3, 4]. Costs incurred by U.S. back pain suffers are staggering, estimated at $90.7 billion5 and growing.14, 45 People with back pain spend 60% more on health care than those without back pain.9, 14 Most people with low back pain do not visit a physician50, 51 because episodes of NLBP resolve spontaneously (Savigny et al., 2009), but of those who do see a doctor, 30% experience pain and disability a year later,40 and few return to normal activities (Savigny et al., 2009).

Businesses lose 100 million work days per year,9 with back pain accounting for 5.5% of all productivity loss in the United States — about $2,200 per employee per year.6, 7 Even employees with minor back pain lose 4.6 hours of productivity a week due to decreased performance on the job.10, 11 Beyond its economic toll, NLBP causes significant physical and psychological suffering.48, 49

Although no consensus has been reached about the best treatment for NLBP, multidisciplinary approaches reduce employee sick leave52-54 and are cost effective.41, 55 However, recommended NLBP treatments often involve specialized clinics, which are costly and not widely available, 2, 41-43 and insurance companies often don’t cover multidisciplinary treatments.40, 56 The Joint Clinical Practice Guidelines from the American College of Physicians and American Pain Society27, 57 recommend inclusion of psychosocial assessments and treatments for back pain, but such assessments are not normally conducted 42 because most physicians lack sufficient time and training to implement recommended procedures. 2, 41 This situation is problematic, however, because early management of NLBP is the best approach to prevent chronic back pain (Savigny et al., 2009). Nonetheless, intervention research on non-medical populations of back pain sufferers is rare.44

An alternative approach is to develop an NLBP intervention that could be widely available on the Internet without requiring medical supervision. A Google search for “backache” had 4,160,000 results and “non-specific low back pain,” had 1,380,000 results, indicating that the Web is a rich source of information for individuals with NSLBP. In a superficial search of these results, however, we found that although many sites offered education or treatment, none offered multidisciplinary behaviorally focused on-line interventions for NLBP, much less any that have been empirically tested for efficacy. Consequently, we developed an on-line program with responsive design architecture (accessible to computers and mobile devices; Adobe 2013) called FitBack, which provided tailored information and support with gain-framed messaging [Latimer A, Salovey P, Rothman A 2007; Rothman AJ, Salovey P 1999]. It was designed for individuals with NLBP to improve their self treatment and pain prevention behaviors.

In the research reported here, we tested FitBack in a randomized design (Clinicaltrials.gov NCT01950091). We hypothesized that the intervention would improve self reported outcomes of functionality and quality of life, that it would be correlated with theoretically relevant psychosocial mediators of behavior change (knowledge, attitudes, self efficacy, behavioral intentions), and that user acceptance would be positive. This was a “real-world” effectiveness trial [125; Flay & Sobel 1983] in the participants’ setting of choice, as opposed to an efficacy trial in a more controlled laboratory setting.

3b) CONSORT: Important changes to methods after trial commencement (such as eligibility criteria), with reasons

Recruitment

After approval by an Institutional Review Board for protection of human subjects (IRB), the study was conducted entirely on the Internet, with recruitment and assessments hosted by surveyconsole.com, a provider of on-line survey tools. The study was conducted in partnership with a large health insurer who promoted the project to client companies.

Four companies (trucking, manufacturing, technology, and a corporate headquarters) with a total of approximately 12,000 employees agreed to promote the research project via their preferred in-house communication channels. Some companies relied on flyers and hard copy media, whereas others used the company website, electronic media, and email.

Recruitment efforts were launched simultaneously in all four companies, but after 30 days, fewer than half the desired number of participants had signed up, and visits by potential participants to the informational website (see below) had declined dramatically. Consequently, while recruitment by the four companies continued, we initiated supplemental online recruitment of participants not affiliated with the 4 companies via craigslist, backdoor.com, trucker websites, online classifieds, and Facebook. We also sent emails to 1200 participants from previous unrelated ORCAS projects who had indicated an interest in possible involvement in future research projects.

Eligibility

Participants were required to (a) be 18 to 65 years of age living in the United States (because it was an NIH grant), (b) be employed at least half-time or retired (or a family member of an employee) at one of the 4 collaborating companies; one participant per family, (c) have experienced low back pain within the past three months, (d) not be experiencing back pain so intense it interfered with everyday life, (e) have no history of medical care for back pain or prescription medications for back pain, (f) not be participating in a monitored exercise program for back pain, (g) have a working email address, (h) respond to an on-line video demonstrating that they had access a computer that could play video on the Internet, (i) be cleared of medical risks by an on-line screening survey (see below). When recruitment was expanded to open Internet enrollment, a new parallel on-line screening process was developed with the same requirements except that all potential participants were required to report that they were employed at least half time.

3b-i) Bug fixes, Downtimes, Content Changes

NA

4a) CONSORT: Eligibility criteria for participants

Participant Screening

Interested individuals linked to an information website that described the research project and eligibility requirements. If still interested, they linked from there to a 5–15 minute on-line screening questionnaire to determine eligibility. The on-line screening questionnaire collected information on demographics, employment status, workplace, and possible medical risk factors.

The screening survey included 40 required questions about back pain history, including current and recent pain and symptoms and health conditions that might contribute to back pain (e.g., history of cancer, infections, fall or motor vehicle accident, osteoporosis, steroid use, bowel or bladder problems, foot drop, rheumatoid arthritis, ankylosing spondylitis, Crohn’s disease, Reiter’s syndrome, numbness in arms or legs, major muscle weakness). These questions were adapted from an instrument developed by the American Pain Society 27,57 in consultation with APS experts and physicians from our health insurance partner. These questions were designed to identify potential program users with medical symptoms or conditions that might be compromised by participating in the research. Individuals who did not meet medical or other eligibility criteria were not accepted to participate.

4a-i) Computer / Internet literacy

Participant Screening

Interested individuals linked to an information website that described the research project and eligibility requirements. If still interested, they linked from there to a 5–15 minute on-line screening questionnaire to determine eligibility. The on-line screening questionnaire collected information on demographics, employment status, workplace, and possible medical risk factors.

The screening survey included 40 required questions about back pain history, including current and recent pain and symptoms and health conditions that might contribute to back pain (e.g., history of cancer, infections, fall or motor vehicle accident, osteoporosis, steroid use, bowel or bladder problems, foot drop, rheumatoid arthritis, ankylosing spondylitis, Crohn’s disease, Reiter’s syndrome, numbness in arms or legs, major muscle weakness). These questions were adapted from an instrument developed by the American Pain Society 27,57 in consultation with APS experts and physicians from our health insurance partner. These questions were designed to identify potential program users with medical symptoms or conditions that might be compromised by participating in the research. Individuals who did not meet medical or other eligibility criteria were not accepted to participate.

4a-ii) Open vs. closed, web-based vs. face-to-face assessments:

Participant Screening
Interested individuals linked to an information website that described the research project and eligibility requirements. If still interested, they linked from there to a 5–15 minute on-line screening questionnaire to determine eligibility. The on-line screening questionnaire collected information on demographics, employment status, workplace, and possible medical risk factors.
The screening survey included 40 required questions about back pain history, including current and recent pain and symptoms and health conditions that might contribute to back pain (e.g., history of cancer, infections, fall or motor vehicle accident, osteoporosis, steroid use, bowel or bladder problems, foot drop, rheumatoid arthritis, ankylosing spondylitis, Crohn's disease, Reiter's syndrome, numbness in arms or legs, major muscle weakness). These questions were adapted from an instrument developed by the American Pain Society 27,57 in consultation with APS experts and physicians from our health insurance partner. These questions were designed to identify potential program users with medical symptoms or conditions that might be compromised by participating in the research. Individuals who did not meet medical or other eligibility criteria were not accepted to participate.
4a-iii) Information giving during recruitment
Recruitment
After approval by an Institutional Review Board for protection of human subjects (IRB), the study was conducted entirely on the Internet, with recruitment and assessments hosted by surveyconsole.com, a provider of on-line survey tools. The study was conducted in partnership with a large health insurer who promoted the project to client companies.
Four companies (trucking, manufacturing, technology, and a corporate headquarters) with a total of approximately 12,000 employees agreed to promote the research project via their preferred in-house communication channels. Some companies relied on flyers and hard copy media, whereas others used the company website, electronic media, and email.
Recruitment efforts were launched simultaneously in all four companies, but after 30 days, fewer than half the desired number of participants had signed up, and visits by potential participants to the informational website (see below) had declined dramatically. Consequently, while recruitment by the four companies continued, we initiated supplemental online recruitment of participants not affiliated with the 4 companies via craigslist, backdoor.com, trucker websites, online classifieds, and Facebook. We also sent emails to 1200 participants from previous unrelated ORCAS projects who had indicated an interest in possible involvement in future research projects.
Eligibility
Participants were required to (a) be 18 to 65 years of age living in the United States (because it was an NIH grant), (b) be employed at least half-time or retired (or a family member of an employee) at one of the 4 collaborating companies; one participant per family, (c) have experienced low back pain within the past three months, (d) not be experiencing back pain so intense it interfered with everyday life, (e) have no history of medical care for back pain or prescription medications for back pain, (f) not be participating in a monitored exercise program for back pain, (g) have a working email address, (h) respond to an on-line video demonstrating that they had access a computer that could play video on the Internet, (i) be cleared of medical risks by an on-line screening survey (see below). When recruitment was expanded to open Internet enrollment, a new parallel on-line screening process was developed with the same requirements except that all potential participants were required to report that they were employed at least half time.
Participant Screening
Interested individuals linked to an information website that described the research project and eligibility requirements. If still interested, they linked from there to a 5–15 minute on-line screening questionnaire to determine eligibility. The on-line screening questionnaire collected information on demographics, employment status, workplace, and possible medical risk factors.
The screening survey included 40 required questions about back pain history, including current and recent pain and symptoms and health conditions that might contribute to back pain (e.g., history of cancer, infections, fall or motor vehicle accident, osteoporosis, steroid use, bowel or bladder problems, foot drop, rheumatoid arthritis, ankylosing spondylitis, Crohn's disease, Reiter's syndrome, numbness in arms or legs, major muscle weakness). These questions were adapted from an instrument developed by the American Pain Society 27,57 in consultation with APS experts and physicians from our health insurance partner. These questions were designed to identify potential program users with medical symptoms or conditions that might be compromised by participating in the research. Individuals who did not meet medical or other eligibility criteria were not accepted to participate.
4b) CONSORT: Settings and locations where the data were collected
Recruitment
After approval by an Institutional Review Board for protection of human subjects (IRB), the study was conducted entirely on the Internet, with recruitment and assessments hosted by surveyconsole.com, a provider of on-line survey tools. The study was conducted in partnership with a large health insurer who promoted the project to client companies.
Four companies (trucking, manufacturing, technology, and a corporate headquarters) with a total of approximately 12,000 employees agreed to promote the research project via their preferred in-house communication channels. Some companies relied on flyers and hard copy media, whereas others used the company website, electronic media, and email.
Recruitment efforts were launched simultaneously in all four companies, but after 30 days, fewer than half the desired number of participants had signed up, and visits by potential participants to the informational website (see below) had declined dramatically. Consequently, while recruitment by the four companies continued, we initiated supplemental online recruitment of participants not affiliated with the 4 companies via craigslist, backdoor.com, trucker websites, online classifieds, and Facebook. We also sent emails to 1200 participants from previous unrelated ORCAS projects who had indicated an interest in possible involvement in future research projects.
Eligibility
Participants were required to (a) be 18 to 65 years of age living in the United States (because it was an NIH grant), (b) be employed at least half-time or retired (or a family member of an employee) at one of the 4 collaborating companies; one participant per family, (c) have experienced low back pain within the past three months, (d) not be experiencing back pain so intense it interfered with everyday life, (e) have no history of medical care for back pain or prescription medications for back pain, (f) not be participating in a monitored exercise program for back pain, (g) have a working email address, (h) respond to an on-line video demonstrating that they had access a computer that could play video on the Internet, (i) be cleared of medical risks by an on-line screening survey (see below). When recruitment was expanded to open Internet enrollment, a new parallel on-line screening process was developed with the same requirements except that all potential participants were required to report that they were employed at least half time.
Participant Screening
Interested individuals linked to an information website that described the research project and eligibility requirements. If still interested, they linked from there to a 5–15 minute on-line screening questionnaire to determine eligibility. The on-line screening questionnaire collected information on demographics, employment status, workplace, and possible medical risk factors.
The screening survey included 40 required questions about back pain history, including current and recent pain and symptoms and health conditions that might contribute to back pain (e.g., history of cancer, infections, fall or motor vehicle accident, osteoporosis, steroid use, bowel or bladder problems, foot drop, rheumatoid arthritis, ankylosing spondylitis, Crohn's disease, Reiter's syndrome, numbness in arms or legs, major muscle weakness). These questions were adapted from an instrument developed by the American Pain Society 27,57 in consultation with APS experts and physicians from our health insurance partner. These questions were designed to identify potential program users with medical symptoms or conditions that might be compromised by participating in the research. Individuals who did not meet medical or other eligibility criteria were not accepted to participate.
4b-i) Report if outcomes were (self-)assessed through online questionnaires

Procedures
After submitting the screening survey, eligible individuals were emailed a link to an informed consent. Participants read and agreed to the consent, after which they provided contact information, including email, mailing address, and telephone number. After their data were checked for fraud (see below), participants were emailed a link to the T1 assessment. Personal privacy was protected with a unique user ID and password for each participant. After submitting the T1 assessment, each participant was emailed his or her experimental group assignment (Tx group, AltCtrl group, Ctrl). Tx and AltCtrl group members subsequently received 8 weekly reminder emails to log on (Tx), or make use of the 6 website links included in the email (AltCtrl). The emails were automated by Mailchimp.com.
The protocol for prompting Tx participants who failed to submit assessments within 4 days of the first email included up to 4 emails at 3-day intervals, followed by a telephone call about 10 days after the fourth email reminder. The call attempted to verify that technical difficulties were not responsible for the lack of participant communication. This protocol was developed based on our experiences in other on-line studies and was approved by our IRB. We believe that it allowed for conscientious follow-up of participants without undo harassment.

4b-ii) Report how institutional affiliations are displayed

Procedures
After submitting the screening survey, eligible individuals were emailed a link to an informed consent. Participants read and agreed to the consent, after which they provided contact information, including email, mailing address, and telephone number. After their data were checked for fraud (see below), participants were emailed a link to the T1 assessment. Personal privacy was protected with a unique user ID and password for each participant. After submitting the T1 assessment, each participant was emailed his or her experimental group assignment (Tx group, AltCtrl group, Ctrl). Tx and AltCtrl group members subsequently received 8 weekly reminder emails to log on (Tx), or make use of the 6 website links included in the email (AltCtrl). The emails were automated by Mailchimp.com.
The protocol for prompting Tx participants who failed to submit assessments within 4 days of the first email included up to 4 emails at 3-day intervals, followed by a telephone call about 10 days after the fourth email reminder. The call attempted to verify that technical difficulties were not responsible for the lack of participant communication. This protocol was developed based on our experiences in other on-line studies and was approved by our IRB. We believe that it allowed for conscientious follow-up of participants without undo harassment.

5) CONSORT: Describe the interventions for each group with sufficient details to allow replication, including how and when they were actually administered

5-i) Mention names, credential, affiliations of the developers, sponsors, and owners

Recruitment
After approval by an Institutional Review Board for protection of human subjects (IRB), the study was conducted entirely on the Internet, with recruitment and assessments hosted by surveyconsole.com, a provider of on-line survey tools. The study was conducted in partnership with a large health insurer who promoted the project to client companies.
Four companies (trucking, manufacturing, technology, and a corporate headquarters) with a total of approximately 12,000 employees agreed to promote the research project via their preferred in-house communication channels. Some companies relied on flyers and hard copy media, whereas others used the company website, electronic media, and email.
Recruitment efforts were launched simultaneously in all four companies, but after 30 days, fewer than half the desired number of participants had signed up, and visits by potential participants to the informational website (see below) had declined dramatically. Consequently, while recruitment by the four companies continued, we initiated supplemental online recruitment of participants not affiliated with the 4 companies via craigslist, backdoor.com, trucker websites, online classifieds, and Facebook. We also sent emails to 1200 participants from previous unrelated ORCAS projects who had indicated an interest in possible involvement in future research projects.

5-ii) Describe the history/development process

Intervention Program
FitBack is a multiple-visit online program to give adults with NLBP education and behavioral strategies to manage current pain episodes and prevent future pain occurrences. The app's responsive design approach (Adobe 2013) allowed users to access the program from multiple devices and screen sizes (smart phone, tablet, computer). The interactive framework was developed in consultation with a panel of physicians and professionals who helped develop the participant medical screening survey (below) to ensure that participants for whom FitBack might create medical risks were excluded from participating in the study . These experts also helped develop content, approved scripts, and conducted usability tests on early versions of FitBack. Care was taken to recommend only activities that the participants could do safely with minimal equipment while unsupervised.
Using a pain tracking tool and gain-framed text and video messages, FitBack helped users develop a self-tailored plan if they were currently in pain, and it encouraged behaviors for prevention of future back pain. Content was tailored by job type: those who sit most of the day (sitters), those who stand most of the day (standers), those who drive most of the day (drivers), and those who do a substantial amount of lifting each day (lifters). Our rationale for a self-tailored approach was based on formative research in this and previous online physical activity studies with sedentary individuals [Irvine et al., 2011; 2013 NCT01579240]) and on the theoretical benefits of behavioral control espoused in social cognitive theory (Bandura 1977; 1986) and the theory of planned behavior (TPB; Ajzen, 1991; Fishbein & Ajzen 2009). Interventions based on TPB have recently been shown to produce large effects on behavior in online interventions [Web et al., 2010].

5-iii) Revisions and updating

NA

5-iv) Quality assurance methods

Intervention Program
FitBack is a multiple-visit online program to give adults with NLBP education and behavioral strategies to manage current pain episodes and prevent future pain occurrences. The app's responsive design approach (Adobe 2013) allowed users to access the program from multiple devices and screen sizes (smart phone, tablet, computer). The interactive framework was developed in consultation with a panel of physicians and professionals who helped develop the participant medical screening survey (below) to ensure that participants for whom FitBack might create medical risks were excluded from participating in the study . These experts also helped develop content, approved scripts, and conducted usability tests on early versions of FitBack. Care was taken to recommend only activities that the participants could do safely with minimal equipment while unsupervised.
Using a pain tracking tool and gain-framed text and video messages, FitBack helped users develop a self-tailored plan if they were currently in pain, and it encouraged behaviors for prevention of future back pain. Content was tailored by job type: those who sit most of the day (sitters), those who stand most of the day (standers), those who drive most of the day (drivers), and those who do a substantial amount of lifting each day (lifters). Our rationale for a self-tailored approach was based on formative research in this and previous online physical activity studies with sedentary individuals [Irvine et al., 2011; 2013 NCT01579240]) and on the theoretical benefits of behavioral control espoused in social cognitive theory (Bandura 1977; 1986) and the theory of planned behavior (TPB; Ajzen, 1991; Fishbein & Ajzen 2009). Interventions based on TPB have recently been shown to produce large effects on behavior in online interventions [Web et al., 2010].

5-v) Ensure replicability by publishing the source code, and/or providing screenshots/screen-capture video, and/or providing flowcharts of the algorithms used

no

5-vi) Digital preservation

no

5-vii) Access

Procedures
After submitting the screening survey, eligible individuals were emailed a link to an informed consent. Participants read and agreed to the consent, after which they provided contact information, including email, mailing address, and telephone number. After their data were checked for fraud (see below), participants were emailed a link to the T1 assessment. Personal privacy was protected with a unique user ID and password for each participant. After submitting the T1 assessment, each participant was emailed his or her experimental group assignment (Tx group, AltCtrl group, Ctrl). Tx and AltCtrl group members subsequently received 8 weekly reminder emails to log on (Tx), or make use of the 6 website links included in the email (AltCtrl). The emails were automated by Mailchimp.com.
The protocol for prompting Tx participants who failed to submit assessments within 4 days of the first email included up to 4 emails at 3-day intervals, followed by a telephone call about 10 days after the fourth email reminder. The call attempted to verify that technical difficulties were not responsible for the lack of participant communication. This protocol was developed based on our experiences in other on-line studies and was approved by our IRB. We believe that it allowed for conscientious follow-up of participants without undo harassment.
Group Assignment
Tx Group members received login information and a link to the FitBack intervention website. The subsequent 8 reminder emails to the Tx group highlighted different aspects of FitBack and encouraged its use, and some participants clicked a box to receive personal emails generated from within the FitBack app. Participants who did not make an initial visit to FitBack within two weeks of the assignment email were telephoned once by the research staff. The call was framed as a check-in to verify the participants were receiving the emails, and the caller encouraged the recipient (in person or via voice mail) to visit the program. Participants were not contacted further by the research staff, and we did not attempt to determine who clicked to open their reminder messages.
The AltCtrl group received an initial email and the 8 reminders emails that included links to 6 websites about NLBP (http://www.webmd.com/back-pain/default.htm, http://www.mayoclinic.com/health/back-pain/DS00171, http://en.wikipedia.org/wiki/Back_pain, http://www.emedicinehealth.com/back_pain/article_em.htm, http://www.nlm.nih.gov/medlineplus/backpain.html, http://orthoinfo.aaos.org/topic.cfm?topic=a00311). The websites provided a choice of popular, educational, and medically oriented online resources. We did not attempt to follow-up with participants who did not open their emails.
After the initial group-assignment email, Ctrl members were contacted by email only with links to the T2 and T3 assessments.

5-viii) Mode of delivery, features/functionalities/components of the intervention and comparator, and the theoretical framework

Procedures
After submitting the screening survey, eligible individuals were emailed a link to an informed consent. Participants read and agreed to the consent, after which they provided contact information, including email, mailing address, and telephone number. After their data were checked for fraud (see below), participants were emailed a link to the T1 assessment. Personal privacy was protected with a unique user ID and password for each participant. After submitting the T1 assessment, each participant was emailed his or her experimental group assignment (Tx group, AltCtrl group, Ctrl). Tx and AltCtrl group members subsequently received 8 weekly reminder emails to log on (Tx), or make use of the 6 website links included in the email (AltCtrl). The emails were automated by Mailchimp.com.
The protocol for prompting Tx participants who failed to submit assessments within 4 days of the first email included up to 4 emails at 3-day intervals, followed by a telephone call about 10 days after the fourth email reminder. The call attempted to verify that technical difficulties were not responsible for the lack of participant communication. This protocol was developed based on our experiences in other on-line studies and was approved by our IRB. We believe that it allowed for conscientious follow-up of participants without undo harassment.
Group Assignment
Tx Group members received login information and a link to the FitBack intervention website. The subsequent 8 reminder emails to the Tx group highlighted different aspects of FitBack and encouraged its use, and some participants clicked a box to receive personal emails generated from within the FitBack app. Participants who did not make an initial visit to FitBack within two weeks of the assignment email were telephoned once by the research staff. The call was framed as a check-in to verify the participants were receiving the emails, and the caller encouraged the recipient (in person or via voice mail) to visit the program. Participants were not contacted further by the research staff, and we did not attempt to determine who clicked to open their reminder messages.
The AltCtrl group received an initial email and the 8 reminders emails that included links to 6 websites about NLBP (http://www.webmd.com/back-pain/default.htm, http://www.mayoclinic.com/health/back-pain/DS00171, http://en.wikipedia.org/wiki/Back_pain, http://www.emedicinehealth.com/back_pain/article_em.htm, http://www.nlm.nih.gov/medlineplus/backpain.html, http://orthoinfo.aaos.org/topic.cfm?topic=a00311). The websites provided a choice of popular, educational, and medically oriented online resources. We did not attempt to follow-up with participants who did not open their emails.
After the initial group-assignment email, Ctrl members were contacted by email only with links to the T2 and T3 assessments.

5-ix) Describe use parameters

Procedures
After submitting the screening survey, eligible individuals were emailed a link to an informed consent. Participants read and agreed to the consent, after which they provided contact information, including email, mailing address, and telephone number. After their data were checked for fraud (see below), participants were emailed a link to the T1 assessment. Personal privacy was protected with a unique user ID and password for each participant. After submitting the T1 assessment, each participant was emailed his or her experimental group assignment (Tx group, AltCtrl group, Ctrl). Tx and AltCtrl group members subsequently received 8 weekly reminder emails to log on (Tx), or make use of the 6 website links included in the email (AltCtrl). The emails were automated by Mailchimp.com.
The protocol for prompting Tx participants who failed to submit assessments within 4 days of the first email included up to 4 emails at 3-day intervals, followed by a telephone call about 10 days after the fourth email reminder. The call attempted to verify that technical difficulties were not responsible for the lack of participant communication. This protocol was developed based on our experiences in other on-line studies and was approved by our IRB. We believe that it allowed for conscientious follow-up of participants without undo harassment.
Group Assignment
Tx Group members received login information and a link to the FitBack intervention website. The subsequent 8 reminder emails to the Tx group highlighted different aspects of FitBack and encouraged its use, and some participants clicked a box to receive personal emails generated from within the FitBack app. Participants who did not make an initial visit to FitBack within two weeks of the assignment email were telephoned once by the research staff. The call was framed as a check-in to verify the participants were receiving the emails, and the caller encouraged the recipient (in person or via voice mail) to visit the program. Participants were not contacted further by the research staff, and we did not attempt to determine who clicked to open their reminder messages.
The AltCtrl group received an initial email and the 8 reminders emails that included links to 6 websites about NLBP (http://www.webmd.com/back-pain/default.htm, http://www.mayoclinic.com/health/back-pain/DS00171, http://en.wikipedia.org/wiki/Back_pain, http://www.emedicinehealth.com/back_pain/article_em.htm, http://www.nlm.nih.gov/medlineplus/backpain.html, http://orthoinfo.aaos.org/topic.cfm?topic=a00311). The websites provided a choice of popular, educational, and medically oriented online resources. We did not attempt to follow-up with participants who did not open their emails.
After the initial group-assignment email, Ctrl members were contacted by email only with links to the T2 and T3 assessments.

5-x) Clarify the level of human involvement

Procedures
After submitting the screening survey, eligible individuals were emailed a link to an informed consent. Participants read and agreed to the consent, after which they provided contact information, including email, mailing address, and telephone number. After their data were checked for fraud (see below), participants were emailed a link to the T1 assessment. Personal privacy was protected with a unique user ID and password for each participant. After submitting the T1 assessment, each participant was emailed his or her experimental group assignment (Tx group, AltCtrl group, Ctrl). Tx and AltCtrl group members subsequently received 8 weekly reminder emails to log on (Tx), or make use of the 6 website links included in the email (AltCtrl). The emails were automated by Mailchimp.com.
The protocol for prompting Tx participants who failed to submit assessments within 4 days of the first email included up to 4 emails at 3-day intervals, followed by a telephone call about 10 days after the fourth email reminder. The call attempted to verify that technical difficulties were not responsible for the lack of participant communication. This protocol was developed based on our experiences in other on-line studies and was approved by our IRB. We believe that it allowed for conscientious follow-up of participants without undo harassment.
Group Assignment
Tx Group members received login information and a link to the FitBack intervention website. The subsequent 8 reminder emails to the Tx group highlighted different aspects of FitBack and encouraged its use, and some participants clicked a box to receive personal emails generated from within the FitBack app. Participants who did not make an initial visit to FitBack within two weeks of the assignment email were telephoned once by the research staff. The call was framed as a check-in to verify the participants were receiving the emails, and the caller encouraged the recipient (in person or via voice mail) to visit the program. Participants were not contacted further by the research staff, and we did not attempt to determine who clicked to open their reminder messages.
The AltCtrl group received an initial email and the 8 reminders emails that included links to 6 websites about NLBP (http://www.webmd.com/back-pain/default.htm, http://www.mayoclinic.com/health/back-pain/DS00171, http://en.wikipedia.org/wiki/Back_pain, http://www.emedicinehealth.com/back_pain/article_em.htm, http://www.nlm.nih.gov/medlineplus/backpain.html, http://orthoinfo.aaos.org/topic.cfm?topic=a00311). The websites provided a choice of popular, educational, and medically oriented online resources. We did not attempt to follow-up with participants who did not open their emails.
After the initial group-assignment email, Ctrl members were contacted by email only with links to the T2 and T3 assessments.

5-xi) Report any prompts/reminders used

Procedures
After submitting the screening survey, eligible individuals were emailed a link to an informed consent. Participants read and agreed to the consent, after which they provided contact information, including email, mailing address, and telephone number. After their data were checked for fraud (see below), participants were emailed a link to the T1 assessment. Personal privacy was protected with a unique user ID and password for each participant. After submitting the T1 assessment, each participant was emailed his or her experimental group assignment (Tx group, AltCtrl group, Ctrl). Tx and AltCtrl group members subsequently received 8 weekly reminder emails to log on (Tx), or make use of the 6 website links included in the email (AltCtrl). The emails were automated by Mailchimp.com.
The protocol for prompting Tx participants who failed to submit assessments within 4 days of the first email included up to 4 emails at 3-day intervals, followed by a telephone call about 10 days after the fourth email reminder. The call attempted to verify that technical difficulties were not responsible for the lack of participant communication. This protocol was developed based on our experiences in other on-line studies and was approved by our IRB. We believe that it allowed for conscientious follow-up of participants without undo harassment.
Group Assignment
Tx Group members received login information and a link to the FitBack intervention website. The subsequent 8 reminder emails to the Tx group highlighted different aspects of FitBack and encouraged its use, and some participants clicked a box to receive personal emails generated from within the FitBack app. Participants who did not make an initial visit to FitBack within two weeks of the assignment email were telephoned once by the research staff. The call was framed as a check-in to verify the participants were receiving the emails, and the caller encouraged the recipient (in person or via voice mail) to visit the program. Participants were not contacted further by the research staff, and we did not attempt to determine who clicked to open their reminder messages.
The AltCtrl group received an initial email and the 8 reminders emails that included links to 6 websites about NLBP (http://www.webmd.com/back-pain/default.htm, http://www.mayoclinic.com/health/back-pain/DS00171, http://en.wikipedia.org/wiki/Back_pain, http://www.emedicinehealth.com/back_pain/article_em.htm, http://www.nlm.nih.gov/medlineplus/backpain.html, http://orthoinfo.aaos.org/topic.cfm?topic=a00311). The websites provided a choice of popular, educational, and medically oriented online resources. We did not attempt to follow-up with participants who did not open their emails.
After the initial group-assignment email, Ctrl members were contacted by email only with links to the T2 and T3 assessments.

5-xii) Describe any co-interventions (incl. training/support)

NA

6a) CONSORT: Completely defined pre-specified primary and secondary outcome measures, including how and when they were assessed

Measures

Back Pain History

An individual's history of back pain is a potentially relevant indicator of the relevance of FitBack to each participant and has been recommended as an indicator of quality of life. (120) To assess participants' back pain history, an 8-item program-specific scale was adapted from the Modified Oswestry Low Back Pain Disability Questionnaire (alpha = .71-.87) 112 which is widely used by physical therapists to assesses functional disability due to low back pain. .

Prevention-helping Behaviors

Four items were designed for the study to assess how often in the past two months participants engaged in behaviors intended to help or prevent back pain (e.g., In the last two months, how often did you do exercises specifically to prevent recurrence of your back pain?). Response options were on a 5-point scale (1=none of the time, 5=most of the time) and a mean score computed with a higher score indicative of more engagement in helping behaviors. The measure showed acceptable reliability (alpha=.76).

Knowledge

Fourteen items designed for the study and based on teaching points in the program assessed improvement in knowledge about back pain (e.g., Fear and worry do not influence the intensity of low back pain; When your back hurts, doing simple back exercises many times a day is the best remedy.). Item response options were "true" and "false." The number of correct items were summed and divided by total number of items to reflect the proportion of items answered correctly.

Functionality and Quality of Life

A 10-item scale, adapted from the Multidimensional Pain Inventory Interference Scale 110 and the Interference Scale of the Brief Pain Inventory, 111 assessed functionality and quality of life during the past two months. Participants were asked how back pain interfered in different areas of their lives (day-to-day activities, mood, and productivity at work). Response options were on a 10-point scale (1=does not interfere, 10=completely interferes) and a mean score computed with a higher score indicative of more interference in daily activities due to back pain. The scaled showed good reliability (alpha=.94).

Patient Activation Measure

The Patient Activation Measure (PAM) is a reliable probabilistic scale that assesses activation of patients to take responsibility for their own health (Hibard et al., 2004; 2005). A 10-item scale was adapted from the PAM short form. Participants were asked about their perceptions of taking responsibility for care for their low back pain. Response options were on a 4-point scale (1=disagree, 4=agree) and a mean score computed with a higher score indicative of better functioning. The scale showed good reliability (alpha=.79).

Self-efficacy

The importance of behavioral self-efficacy to engage in recommended behaviors is supported by both social cognitive theory (Bandura 1977; 1986) and the TPB (Ajzen, 1991; Fishbein & Ajzen 2009). To assess this construct, a 13-item scale was adapted from the short form of the Brief Pain Inventory. 111 Participants were asked how confident they were in their ability to use the behaviors recommended in FitBack (e.g., How confident are you in your ability to use back exercises to reduce your low back pain?). Response options were on a 7-point scale (1=not at all confident, 7=extremely confident) and a mean score computed with a higher score indicative of greater levels of self-efficacy to use the practices taught in the program. The scale showed good reliability (alpha=.93).

Behavioral Intentions

The TPB suggests that behavioral intentions can predict adoption of new behaviors (Ajzen, 1991; Fishbein & Ajzen 2009). To assess participant intentions to perform the activities recommended in the program, a 14-item scale was created (e.g., The next time you experience back pain, how likely is it that you will take action to use the right amount of activity to help you get better faster?). Response options were on a 7-point scale (1=not at all confident, 7=extremely confident) and a mean score computed with a higher score indicative of more intention to perform the activities. The scale showed good reliability (alpha=.90).

Attitudes Toward Pain

Attitudes toward pain complicate perceptions of pain and quality of life119-120 and are linked by the TPB to self confidence and intentions to attempt behavioral remedies (Ajzen, 1991; Fishbein & Ajzen 2009). A 10-item adaptation of the short version of the Survey of Pain Attitudes (SOPA) (Tait & Chibnall,1997; Jensen et al., 1994) was used to assess participants' attitudes toward their pain. The items formed two subscales: a 6-item control scale to assess the extent to which a person believes s/he can control pain, and a 4-item emotion scale to assess the extent to which a person believes his/her emotions affect the experience of pain. Response options were on a 5-point scale (1=very untrue for me, 5=very true for me) and a mean score computed for each scale with a higher score indicative of more positive attitudes. Both the control and emotion scales showed good reliability (alphas=. 81 and .95, respectively).

Catastrophizing of Pain

Fear of pain might indicate vulnerability or a tendency to catastrophize about a painful problem. A 4-item scale that explains 54% of the variance of the Tampa Scale for Kinesiophobia (Burwinkle et al., 2005), was adapted to assess the degree to which a participant catastrophizes pain (e.g., My back pain puts my body at risk for the rest of my life). Items were assessed on a 4-point scale (1=strongly disagree, 5=strongly agree) and a mean score computed with a higher score indicative of greater levels of catastrophizing. The scale showed adequate reliability (alpha=.77).

Worker Productivity

The 4-item Work Limitations Questionnaire (WLQ), a reliable measure of how osteoarthritis affects worker productivity (Lerner et al., 2002), was used to assess the degree to which a participant's back pain interfered with work (e.g., In the past two weeks, how much time did your physical health or emotional health problems make it difficult for you to get going at the beginning of the workday?). Response options were on a 5-point scale (1=difficult all of the time, 5=difficult none of the time) and a mean score computed with a higher score indicative of greater productivity. The scale showed adequate reliability (alpha=.76).

Presenteeism

The concept of presenteeism encompasses workers who are present at their jobs but are underperforming in terms of quantity or quality due to an injury or health condition. The six item Stanford Presenteeism scale (Koopman et al., 2002) was adapted to assess the extent to which workers' back pain inhibited them from doing their jobs (e.g., "Despite having my back pain, I was able to finish hard tasks in my work."). Response options were on a 5-point scale (1=strongly disagree, 5=strongly agree) and mean score computed with a higher score indicative of more effective work practices. The scale showed adequate reliability (alpha=.77).

Dartmouth COOP

The 9-item Dartmouth COOP scale (Nelson et al., 1997) measures different aspects of patient health status, including function (physical endurance, emotional health, role function, and social function), well-being (overall health, change in health, level of pain), and quality of life (overall quality of life and social resources/support). Response options were on a 5-point scale and differed by item, with a higher score indicative of poorer health status for each scale. A total sum score was computed, and the scale showed adequate reliability (alpha=.78).

User Satisfaction

The satisfaction of users with program information and components might be a good relative measure of participant acceptance of an intervention, which in turn could increase the likelihood of follow-through with program recommendations over time. For this reason we developed multiple measures to assess user satisfaction.

Four items were administered at T2 and T3 to both Tx and AltCtrl group participants for comparison purposes. They included satisfaction with information on back health provided, likelihood of recommending the resources to a friend, and value in helping self treatment of low back pain occurrence and helping prevent back pain occurrence. Response options were on a 7-point scale (1=not at all, 7=extremely) with a higher score indicative of greater satisfaction with the program.

Website Usability

Website Usability
The System Usability Scale (SUS) is a 10-item survey referenced in more than 600 publications (Sauro, 2011). The SUS is a well-used tool for assessing the usability of a product, including websites, cell phones, interactive voice response systems, and TV applications (Bangor, Kortum, & Miller, 2009), and we used it to ascertain participants' attitudes toward the functionality of the FitBack program. It consists of five positively worded items (e.g., "I think that I would like to use FitBack frequently"; "I found the various functions in FitBack were well integrated.") and five negatively worded items (e.g., "I found FitBack unnecessarily complex"; "I think that I would need the support of a technical person to be able to use FitBack.") on a 5-point agree–disagree rating scale. When scoring the SUS, the items are rescaled so that when they are summed they range from 0 to 100. An overall sum score was computed with a higher score indicative of more positive attitudes toward the program. The SUS can be scored as a percentile rank and compared with 500 other studies in a process comparable to grading on a curve, with a score of 68 considered average (Bangor et al., 2009; Sauro, 2011).
Perceptions of Employers
Five items were used to assess how employees would view an employer who made the FitBack program available. The stem was "If my employer made FitBack available to all company employees," and responses included, "I would feel like my company cares about me"; "I would feel more positive about my company"; "I would feel a greater commitment to my company"; "I would be more productive at my job"; and I would feel more satisfied with my job." Response options were on 6-point scale (1=strongly disagree, 6=strongly agree) with a higher score indicative of a more favorable impression of the employer.
Understanding and Implementation Survey
A seven item survey was designed to ascertain to what degree the participant understood and implemented the teaching points of the FitBack program (e.g., Did you understand program recommendations about using heat or ice to help deal with back pain when it occurs?). Categorical response options were "yes," "yes, somewhat," "no," not much," and "not at all."
6a-i) Online questionnaires: describe if they were validated for online use and apply CHERRIES items to describe how the questionnaires were designed/deployed

What's a CHERRIES?

Measures

Back Pain History

An individual's history of back pain is a potentially relevant indicator of the relevance of FitBack to each participant and has been recommended as an indicator of quality of life. (120) To assess participants' back pain history, an 8-item program-specific scale was adapted from the Modified Oswestry Low Back Pain Disability Questionnaire (alpha = .71-.87) 112 which is widely used by physical therapists to assesses functional disability due to low back pain. .

Prevention-helping Behaviors

Four items were designed for the study to assess how often in the past two months participants engaged in behaviors intended to help or prevent back pain (e.g., In the last two months, how often did you do exercises specifically to prevent recurrence of your back pain?). Response options were on a 5-point scale (1=none of the time, 5=most of the time) and a mean score computed with a higher score indicative of more engagement in helping behaviors. The measure showed acceptable reliability (alpha=.76).

Knowledge

Fourteen items designed for the study and based on teaching points in the program assessed improvement in knowledge about back pain (e.g., Fear and worry do not influence the intensity of low back pain; When your back hurts, doing simple back exercises many times a day is the best remedy.). Item response options were "true" and "false." The number of correct items were summed and divided by total number of items to reflect the proportion of items answered correctly.

Functionality and Quality of Life

A 10-item scale, adapted from the Multidimensional Pain Inventory Interference Scale 110 and the Interference Scale of the Brief Pain Inventory, 111 assessed functionality and quality of life during the past two months. Participants were asked how back pain interfered in different areas of their lives (day-to-day activities, mood, and productivity at work). Response options were on a 10-point scale (1=does not interfere, 10=completely interferes) and a mean score computed with a higher score indicative of more interference in daily activities due to back pain. The scaled showed good reliability (alpha=.94).

Patient Activation Measure

The Patient Activation Measure (PAM) is a reliable probabilistic scale that assesses activation of patients to take responsibility for their own health (Hibard et al., 2004; 2005). A 10-item scale was adapted from the PAM short form. Participants were asked about their perceptions of taking responsibility for care for their low back pain. Response options were on a 4-point scale (1=disagree, 4=agree) and a mean score computed with a higher score indicative of better functioning. The scale showed good reliability (alpha=.79).

Self-efficacy

The importance of behavioral self-efficacy to engage in recommended behaviors is supported by both social cognitive theory (Bandura 1977; 1986) and the TPB (Ajzen, 1991; Fishbein & Ajzen 2009). To assess this construct, a 13-item scale was adapted from the short form of the Brief Pain Inventory. 111 Participants were asked how confident they were in their ability to use the behaviors recommended in FitBack (e.g., How confident are you in your ability to use back exercises to reduce your low back pain?). Response options were on a 7-point scale (1=not at all confident, 7=extremely confident) and a mean score computed with a higher score indicative of greater levels of self-efficacy to use the practices taught in the program. The scale showed good reliability (alpha=.93).

Behavioral Intentions

The TPB suggests that behavioral intentions can predict adoption of new behaviors (Ajzen, 1991; Fishbein & Ajzen 2009). To assess participant intentions to perform the activities recommended in the program, a 14-item scale was created (e.g., The next time you experience back pain, how likely is it that you will take action to use the right amount of activity to help you get better faster?). Response options were on a 7-point scale (1=not at all confident, 7=extremely confident) and a mean score computed with a higher score indicative of more intention to perform the activities. The scale showed good reliability (alpha=.90).

Attitudes Toward Pain

Attitudes toward pain complicate perceptions of pain and quality of life119-120 and are linked by the TPB to self confidence and intentions to attempt behavioral remedies (Ajzen, 1991; Fishbein & Ajzen 2009). A 10-item adaptation of the short version of the Survey of Pain Attitudes (SOPA) (Tait & Chibnall,1997; Jensen et al., 1994) was used to assess participants' attitudes toward their pain. The items formed two subscales: a 6-item control scale to assess the extent to which a person believes s/he can control pain, and a 4-item emotion scale to assess the extent to which a person believes his/her emotions affect the experience of pain. Response options were on a 5-point scale (1=very untrue for me, 5=very true for me) and a mean score computed for each scale with a higher score indicative of more positive attitudes. Both the control and emotion scales showed good reliability (alphas=. 81 and .95, respectively).

Catastrophizing of Pain

Fear of pain might indicate vulnerability or a tendency to catastrophize about a painful problem. A 4-item scale that explains 54% of the variance of the Tampa Scale for Kinesiophobia (Burwinkle et al., 2005), was adapted to assess the degree to which a participant catastrophizes pain (e.g., My back pain puts my body at risk for the rest of my life). Items were assessed on a 4-point scale (1=strongly disagree, 5=strongly agree) and a mean score computed with a higher score indicative of greater levels of catastrophizing. The scale showed adequate reliability (alpha=.77).

Worker Productivity

The 4-item Work Limitations Questionnaire (WLQ), a reliable measure of how osteoarthritis affects worker productivity (Lerner et al., 2002), was used to assess the degree to which a participant's back pain interfered with work (e.g., In the past two weeks, how much time did your physical health or emotional health problems make it difficult for you to get going at the beginning of the workday?). Response options were on a 5-point scale (1=difficult all of the time, 5=difficult none of the time) and a mean score computed with a higher score indicative of greater productivity. The scale showed adequate reliability (alpha=.76).

Presenteeism

The concept of presenteeism encompasses workers who are present at their jobs but are underperforming in terms of quantity or quality due to an injury or health condition. The six item Stanford Presenteeism scale (Koopman et al., 2002) was adapted to assess the extent to which workers' back pain inhibited them from doing their jobs (e.g., "Despite having my back pain, I was able to finish hard tasks in my work."). Response options were on a 5-point scale (1=strongly disagree, 5=strongly agree) and mean score computed with a higher score indicative of more effective work practices. The scale showed adequate reliability (alpha=.77).

Dartmouth COOP

The 9-item Dartmouth COOP scale (Nelson et al., 1997) measures different aspects of patient health status, including function (physical endurance, emotional health, role function, and social function), well-being (overall health, change in health, level of pain), and quality of life (overall quality of life and social resources/support). Response options were on a 5-point scale and differed by item, with a higher score indicative of poorer health status for each scale. A total sum score was computed, and the scale showed adequate reliability (alpha=.78).

User Satisfaction

The satisfaction of users with program information and components might be a good relative measure of participant acceptance of an intervention, which in turn could increase the likelihood of follow-through with program recommendations over time. For this reason we developed multiple measures to assess user satisfaction.

Four items were administered at T2 and T3 to both Tx and AltCtrl group participants for comparison purposes. They included satisfaction with information on back health provided, likelihood of recommending the resources to a friend, and value in helping self treatment of low back pain occurrence and helping prevent back pain occurrence. Response options were on a 7-point scale (1=not at all, 7=extremely) with a higher score indicative of greater satisfaction with the program

indicative of greater satisfaction with the program.
Website Usability
The System Usability Scale (SUS) is a 10-item survey referenced in more than 600 publications (Sauro, 2011). The SUS is a well-used tool for assessing the usability of a product, including websites, cell phones, interactive voice response systems, and TV applications (Bangor, Kortum, & Miller, 2009), and we used it to ascertain participants' attitudes toward the functionality of the FitBack program. It consists of five positively worded items (e.g., “I think that I would like to use FitBack frequently”; “I found the various functions in FitBack were well integrated.”) and five negatively worded items (e. g., “I found FitBack unnecessarily complex”; “I think that I would need the support of a technical person to be able to use FitBack.”) on a 5-point agree–disagree rating scale. When scoring the SUS, the items are rescaled so that when they are summed they range from 0 to 100. An overall sum score was computed with a higher score indicative of more positive attitudes toward the program. The SUS can be scored as a percentile rank and compared with 500 other studies in a process comparable to grading on a curve, with a score of 68 considered average (Bangor et al., 2009; Sauro, 2011).
Perceptions of Employers
Five items were used to assess how employees would view an employer who made the FitBack program available. The stem was “If my employer made FitBack available to all company employees,” and responses included, “I would feel like my company cares about me”; “I would feel more positive about my company”; “I would feel a greater commitment to my company”; “I would be more productive at my job”; and I would feel more satisfied with my job.” Response options were on 6-point scale (1=strongly disagree, 6=strongly agree) with a higher score indicative of a more favorable impression of the employer.
Understanding and Implementation Survey
A seven item survey was designed to ascertain to what degree the participant understood and implemented the teaching points of the FitBack program (e.g., Did you understand program recommendations about using heat or ice to help deal with back pain when it occurs?). Categorical response options were “yes,” “yes, somewhat,” “no,” not much,” and “not at all.”

6a-ii) Describe whether and how “use” (including intensity of use/dosage) was defined/measured/monitored

Statistical Methods
Preliminary Analysis
Chi-square tests and one-way analysis of variance models were used to compare the three groups on the demographic characteristics, baseline outcomes, and baseline back pain measures. None were found to be statistically significant at $p<.05$, suggesting randomization produced initially equivalent groups.
All 597 participants completed the T1 assessment, 586 (98%) completed T2, and 582 (98%) completed T3. Participants who completed all three assessments (n=590, 97%) were compared to those who did not (n=17, 3%) on study condition, demographic characteristics, baseline outcomes, and baseline back pain measures. No statistically significant differences were found with the exception of the baseline intention score. Participants who did not complete all three assessments had significantly (t-value=2.22, p=.049) lower intention scores than those who did complete all assessments (3.45 vs. 4.07, respectively). However, mean differences were associated with a small effect size (Cohen's d=.27; Cohen, 1988).
Missing Data
Rates of missing data ranged from 0–5% at T1, 2–5% at T2, and 3–8% at T3. Despite the low rates of missing data, one fully imputed data set was generated for this intent-to-treat analysis as it produces less bias then other missing data techniques, such as list-wise deletion and last observation carried forward (Shafer and Graham, 2002). Missing data were imputed using IVEWare (Raghunathan, Solenberger, & Van Hoewyk, 2002), which uses all available data to impute missing data via a sequential regression approach. The observed and imputed data were compared to ensure they showed similar distributions (Abayomi, Gelman, & Levy, 2008).
Analytic Models
Primary analysis included analysis of covariance (ANCOVA) models to test for group differences on study outcomes at T2 and T3, separately, with the T1 score as a covariate and study condition as a three-level predictor (1=treatment, 2=alternative care, 3=control). If the overall omnibus test was significant, then follow-up planned contrasts (treatment vs. alternative care and treatment vs. control) were examined. Eta-square is provided as a measure of effects size with the convention .01 small, .06 moderate, and .14 large (Cohen, 1988). Next, the moderating effects of gender and age were tested at T2 and T3 by entering the moderator and the moderator by study condition interaction term. A Benjamini-Hochberg false discovery rate correction (Benjamini, & Hochberg 1995) was made to overall tests of main effects, planned contrasts, and moderating models to help protect against Type-I errors.
Secondary analysis included examination of the survey item that ascertained whether or not a study participant was currently experiencing back pain (“yes” or “no” response option). Logistic regression models were used to determine whether study condition predicted current back pain status at T2 and T3, separately, with the T1 response as a covariate. A within-subject dose response analysis was also performed with the FitBack intervention participants to test the hypothesis that those participants who used the program more showed greater gains in the study outcomes. Total time on the site was obtained from the log files and correlated with change scores (T2 minus T1 scores and T3 minus T1 scores). Finally, independent t-tests were used to compare the FitBack participants and alternative care participants on four program satisfaction items administered as part of the T2 and T3 surveys.

6a-iii) Describe whether, how, and when qualitative feedback from participants was obtained

NA

6b) CONSORT: Any changes to trial outcomes after the trial commenced, with reasons

NA

7a) CONSORT: How sample size was determined

7a-i) Describe whether and how expected attrition was taken into account when calculating the sample size

Statistical Methods

Preliminary Analysis

Chi-square tests and one-way analysis of variance models were used to compare the three groups on the demographic characteristics, baseline outcomes, and baseline back pain measures. None were found to be statistically significant at $p<.05$, suggesting randomization produced initially equivalent groups.

All 597 participants completed the T1 assessment, 586 (98%) completed T2, and 582 (98%) completed T3. Participants who completed all three assessments (n=590, 97%) were compared to those who did not (n=17, 3%) on study condition, demographic characteristics, baseline outcomes, and baseline back pain measures. No statistically significant differences were found with the exception of the baseline intention score. Participants who did not complete all three assessments had significantly (t-value=2.22, $p=.049$) lower intention scores than those who did complete all assessments (3.45 vs. 4.07, respectively). However, mean differences were associated with a small effect size (Cohen's d=.27; Cohen, 1988).

Missing Data

Rates of missing data ranged from 0–5% at T1, 2–5% at T2, and 3–8% at T3. Despite the low rates of missing data, one fully imputed data set was generated for this intent-to-treat analysis as it produces less bias then other missing data techniques, such as list-wise deletion and last observation carried forward (Shafer and Graham, 2002). Missing data were imputed using IVEWare (Raghunathan, Solenberger, & Van Hoewyk, 2002), which uses all available data to impute missing data via a sequential regression approach. The observed and imputed data were compared to ensure they showed similar distributions (Abayomi, Gelman, & Levy, 2008).

Analytic Models

Primary analysis included analysis of covariance (ANCOVA) models to test for group differences on study outcomes at T2 and T3, separately, with the T1 score as a covariate and study condition as a three-level predictor (1=treatment, 2=alternative care, 3=control). If the overall omnibus test was significant, then follow-up planned contrasts (treatment vs. alternative care and treatment vs. control) were examined. Eta-square is provided as a measure of effects size with the convention .01 small, .06 moderate, and .14 large (Cohen, 1988). Next, the moderating effects of gender and age were tested at T2 and T3 by entering the moderator and the moderator by study condition interaction term. A Benjamini-Hochberg false discovery rate correction (Benjamini, & Hochberg 1995) was made to overall tests of main effects, planned contrasts, and moderating models to help protect against Type-I errors.

Secondary analysis included examination of the survey item that ascertained whether or not a study participant was currently experiencing back pain ("yes" or "no" response option). Logistic regression models were used to determine whether study condition predicted current back pain status at T2 and T3, separately, with the T1 response as a covariate. A within-subject dose response analysis was also performed with the FitBack intervention participants to test the hypothesis that those participants who used the program more showed greater gains in the study outcomes. Total time on the site was obtained from the log files and correlated with change scores (T2 minus T1 scores and T3 minus T1 scores). Finally, independent t-tests were used to compare the FitBack participants and alternative care participants on four program satisfaction items administered as part of the T2 and T3 surveys.

7b) CONSORT: When applicable, explanation of any interim analyses and stopping guidelines

Statistical Methods

Preliminary Analysis

Chi-square tests and one-way analysis of variance models were used to compare the three groups on the demographic characteristics, baseline outcomes, and baseline back pain measures. None were found to be statistically significant at $p<.05$, suggesting randomization produced initially equivalent groups.

All 597 participants completed the T1 assessment, 586 (98%) completed T2, and 582 (98%) completed T3. Participants who completed all three assessments (n=590, 97%) were compared to those who did not (n=17, 3%) on study condition, demographic characteristics, baseline outcomes, and baseline back pain measures. No statistically significant differences were found with the exception of the baseline intention score. Participants who did not complete all three assessments had significantly (t-value=2.22, $p=.049$) lower intention scores than those who did complete all assessments (3.45 vs. 4.07, respectively). However, mean differences were associated with a small effect size (Cohen's d=.27; Cohen, 1988).

Missing Data

Rates of missing data ranged from 0–5% at T1, 2–5% at T2, and 3–8% at T3. Despite the low rates of missing data, one fully imputed data set was generated for this intent-to-treat analysis as it produces less bias then other missing data techniques, such as list-wise deletion and last observation carried forward (Shafer and Graham, 2002). Missing data were imputed using IVEWare (Raghunathan, Solenberger, & Van Hoewyk, 2002), which uses all available data to impute missing data via a sequential regression approach. The observed and imputed data were compared to ensure they showed similar distributions (Abayomi, Gelman, & Levy, 2008).

Analytic Models

Primary analysis included analysis of covariance (ANCOVA) models to test for group differences on study outcomes at T2 and T3, separately, with the T1 score as a covariate and study condition as a three-level predictor (1=treatment, 2=alternative care, 3=control). If the overall omnibus test was significant, then follow-up planned contrasts (treatment vs. alternative care and treatment vs. control) were examined. Eta-square is provided as a measure of effects size with the convention .01 small, .06 moderate, and .14 large (Cohen, 1988). Next, the moderating effects of gender and age were tested at T2 and T3 by entering the moderator and the moderator by study condition interaction term. A Benjamini-Hochberg false discovery rate correction (Benjamini, & Hochberg 1995) was made to overall tests of main effects, planned contrasts, and moderating models to help protect against Type-I errors.

Secondary analysis included examination of the survey item that ascertained whether or not a study participant was currently experiencing back pain ("yes" or "no" response option). Logistic regression models were used to determine whether study condition predicted current back pain status at T2 and T3, separately, with the T1 response as a covariate. A within-subject dose response analysis was also performed with the FitBack intervention participants to test the hypothesis that those participants who used the program more showed greater gains in the study outcomes. Total time on the site was obtained from the log files and correlated with change scores (T2 minus T1 scores and T3 minus T1 scores). Finally, independent t-tests were used to compare the FitBack participants and alternative care participants on four program satisfaction items administered as part of the T2 and T3 surveys.

8a) CONSORT: Method used to generate the random allocation sequence

Statistical Methods
Preliminary Analysis
Chi-square tests and one-way analysis of variance models were used to compare the three groups on the demographic characteristics, baseline outcomes, and baseline back pain measures. None were found to be statistically significant at $p<.05$, suggesting randomization produced initially equivalent groups.
All 597 participants completed the T1 assessment, 586 (98%) completed T2, and 582 (98%) completed T3. Participants who completed all three assessments (n=590, 97%) were compared to those who did not (n=17, 3%) on study condition, demographic characteristics, baseline outcomes, and baseline back pain measures. No statistically significant differences were found with the exception of the baseline intention score. Participants who did not complete all three assessments had significantly (t-value=2.22, p=.049) lower intention scores than those who did complete all assessments (3.45 vs. 4.07, respectively). However, mean differences were associated with a small effect size (Cohen's d=.27; Cohen, 1988).
Missing Data
Rates of missing data ranged from 0–5% at T1, 2–5% at T2, and 3–8% at T3. Despite the low rates of missing data, one fully imputed data set was generated for this intent-to-treat analysis as it produces less bias then other missing data techniques, such as list-wise deletion and last observation carried forward (Shafer and Graham, 2002). Missing data were imputed using IVEWare (Raghunathan, Solenberger, & Van Hoewyk, 2002), which uses all available data to impute missing data via a sequential regression approach. The observed and imputed data were compared to ensure they showed similar distributions (Abayomi, Gelman, & Levy, 2008).
Analytic Models
Primary analysis included analysis of covariance (ANCOVA) models to test for group differences on study outcomes at T2 and T3, separately, with the T1 score as a covariate and study condition as a three-level predictor (1=treatment, 2=alternative care, 3=control). If the overall omnibus test was significant, then follow-up planned contrasts (treatment vs. alternative care and treatment vs. control) were examined. Eta-square is provided as a measure of effects size with the convention .01 small, .06 moderate, and .14 large (Cohen, 1988). Next, the moderating effects of gender and age were tested at T2 and T3 by entering the moderator and the moderator by study condition interaction term. A Benjamini-Hochberg false discovery rate correction (Benjamini, & Hochberg 1995) was made to overall tests of main effects, planned contrasts, and moderating models to help protect against Type-I errors.
Secondary analysis included examination of the survey item that ascertained whether or not a study participant was currently experiencing back pain ("yes" or "no" response option). Logistic regression models were used to determine whether study condition predicted current back pain status at T2 and T3, separately, with the T1 response as a covariate. A within-subject dose response analysis was also performed with the FitBack intervention participants to test the hypothesis that those participants who used the program more showed greater gains in the study outcomes. Total time on the site was obtained from the log files and correlated with change scores (T2 minus T1 scores and T3 minus T1 scores). Finally, independent t-tests were used to compare the FitBack participants and alternative care participants on four program satisfaction items administered as part of the T2 and T3 surveys.

8b) CONSORT: Type of randomisation; details of any restriction (such as blocking and block size)

Statistical Methods
Preliminary Analysis
Chi-square tests and one-way analysis of variance models were used to compare the three groups on the demographic characteristics, baseline outcomes, and baseline back pain measures. None were found to be statistically significant at $p<.05$, suggesting randomization produced initially equivalent groups.
All 597 participants completed the T1 assessment, 586 (98%) completed T2, and 582 (98%) completed T3. Participants who completed all three assessments (n=590, 97%) were compared to those who did not (n=17, 3%) on study condition, demographic characteristics, baseline outcomes, and baseline back pain measures. No statistically significant differences were found with the exception of the baseline intention score. Participants who did not complete all three assessments had significantly (t-value=2.22, p=.049) lower intention scores than those who did complete all assessments (3.45 vs. 4.07, respectively). However, mean differences were associated with a small effect size (Cohen's d=.27; Cohen, 1988).
Missing Data
Rates of missing data ranged from 0–5% at T1, 2–5% at T2, and 3–8% at T3. Despite the low rates of missing data, one fully imputed data set was generated for this intent-to-treat analysis as it produces less bias then other missing data techniques, such as list-wise deletion and last observation carried forward (Shafer and Graham, 2002). Missing data were imputed using IVEWare (Raghunathan, Solenberger, & Van Hoewyk, 2002), which uses all available data to impute missing data via a sequential regression approach. The observed and imputed data were compared to ensure they showed similar distributions (Abayomi, Gelman, & Levy, 2008).
Analytic Models
Primary analysis included analysis of covariance (ANCOVA) models to test for group differences on study outcomes at T2 and T3, separately, with the T1 score as a covariate and study condition as a three-level predictor (1=treatment, 2=alternative care, 3=control). If the overall omnibus test was significant, then follow-up planned contrasts (treatment vs. alternative care and treatment vs. control) were examined. Eta-square is provided as a measure of effects size with the convention .01 small, .06 moderate, and .14 large (Cohen, 1988). Next, the moderating effects of gender and age were tested at T2 and T3 by entering the moderator and the moderator by study condition interaction term. A Benjamini-Hochberg false discovery rate correction (Benjamini, & Hochberg 1995) was made to overall tests of main effects, planned contrasts, and moderating models to help protect against Type-I errors.
Secondary analysis included examination of the survey item that ascertained whether or not a study participant was currently experiencing back pain ("yes" or "no" response option). Logistic regression models were used to determine whether study condition predicted current back pain status at T2 and T3, separately, with the T1 response as a covariate. A within-subject dose response analysis was also performed with the FitBack intervention participants to test the hypothesis that those participants who used the program more showed greater gains in the study outcomes. Total time on the site was obtained from the log files and correlated with change scores (T2 minus T1 scores and T3 minus T1 scores). Finally, independent t-tests were used to compare the FitBack participants and alternative care participants on four program satisfaction items administered as part of the T2 and T3 surveys.

9) CONSORT: Mechanism used to implement the random allocation sequence (such as sequentially numbered containers), describing any steps taken to conceal the sequence until interventions were assigned

NA

10) CONSORT: Who generated the random allocation sequence, who enrolled participants, and who assigned participants to interventions

we did

11a) CONSORT: Blinding - If done, who was blinded after assignment to interventions (for example, participants, care providers, those assessing outcomes) and how

11a-i) Specify who was blinded, and who wasn't

NA

11a-ii) Discuss e.g., whether participants knew which intervention was the "intervention of interest" and which one was the "comparator"

NA

11b) CONSORT: If relevant, description of the similarity of interventions

Group Assignment
Tx Group members received login information and a link to the FitBack intervention website. The subsequent 8 reminder emails to the Tx group highlighted different aspects of FitBack and encouraged its use, and some participants clicked a box to receive personal emails generated from within the FitBack app. Participants who did not make an initial visit to FitBack within two weeks of the assignment email were telephoned once by the research staff. The call was framed as a check-in to verify the participants were receiving the emails, and the caller encouraged the recipient (in person or via voice mail) to visit the program. Participants were not contacted further by the research staff, and we did not attempt to determine who clicked to open their reminder messages.
The AltCtrl group received an initial email and the 8 reminders emails that included links to 6 websites about NLBP (http://www.webmd.com/back-pain/default.htm, http://www.mayoclinic.com/health/back-pain/DS00171, http://en.wikipedia.org/wiki/Back_pain, http://www.emedicinehealth.com/back_pain/article_em.htm, http://www.nlm.nih.gov/medlineplus/backpain.html, http://orthoinfo.aaos.org/topic.cfm?topic=a00311). The websites provided a choice of popular, educational, and medically oriented online resources. We did not attempt to follow-up with participants who did not open their emails.
After the initial group-assignment email, Ctrl members were contacted by email only with links to the T2 and T3 assessments.

12a) CONSORT: Statistical methods used to compare groups for primary and secondary outcomes

Statistical Methods
Preliminary Analysis
Chi-square tests and one-way analysis of variance models were used to compare the three groups on the demographic characteristics, baseline outcomes, and baseline back pain measures. None were found to be statistically significant at $p<.05$, suggesting randomization produced initially equivalent groups.
All 597 participants completed the T1 assessment, 586 (98%) completed T2, and 582 (98%) completed T3. Participants who completed all three assessments (n=590, 97%) were compared to those who did not (n=17, 3%) on study condition, demographic characteristics, baseline outcomes, and baseline back pain measures. No statistically significant differences were found with the exception of the baseline intention score. Participants who did not complete all three assessments had significantly (t-value=2.22, p=.049) lower intention scores than those who did complete all assessments (3.45 vs. 4.07, respectively). However, mean differences were associated with a small effect size (Cohen’s d=.27; Cohen, 1988).
Missing Data
Rates of missing data ranged from 0–5% at T1, 2–5% at T2, and 3–8% at T3. Despite the low rates of missing data, one fully imputed data set was generated for this intent-to-treat analysis as it produces less bias then other missing data techniques, such as list-wise deletion and last observation carried forward (Shafer and Graham, 2002). Missing data were imputed using IVEWare (Raghunathan, Solenberger, & Van Hoewyk, 2002), which uses all available data to impute missing data via a sequential regression approach. The observed and imputed data were compared to ensure they showed similar distributions (Abayomi, Gelman, & Levy, 2008).
Analytic Models
Primary analysis included analysis of covariance (ANCOVA) models to test for group differences on study outcomes at T2 and T3, separately, with the T1 score as a covariate and study condition as a three-level predictor (1=treatment, 2=alternative care, 3=control). If the overall omnibus test was significant, then follow-up planned contrasts (treatment vs. alternative care and treatment vs. control) were examined. Eta-square is provided as a measure of effects size with the convention .01 small, .06 moderate, and .14 large (Cohen, 1988). Next, the moderating effects of gender and age were tested at T2 and T3 by entering the moderator and the moderator by study condition interaction term. A Benjamini-Hochberg false discovery rate correction (Benjamini, & Hochberg 1995) was made to overall tests of main effects, planned contrasts, and moderating models to help protect against Type-I errors.
Secondary analysis included examination of the survey item that ascertained whether or not a study participant was currently experiencing back pain (“yes” or “no” response option). Logistic regression models were used to determine whether study condition predicted current back pain status at T2 and T3, separately, with the T1 response as a covariate. A within-subject dose response analysis was also performed with the FitBack intervention participants to test the hypothesis that those participants who used the program more showed greater gains in the study outcomes. Total time on the site was obtained from the log files and correlated with change scores (T2 minus T1 scores and T3 minus T1 scores). Finally, independent t-tests were used to compare the FitBack participants and alternative care participants on four program satisfaction items administered as part of the T2 and T3 surveys.

12a-i) Imputation techniques to deal with attrition / missing values

Statistical Methods
Preliminary Analysis
Chi-square tests and one-way analysis of variance models were used to compare the three groups on the demographic characteristics, baseline outcomes, and baseline back pain measures. None were found to be statistically significant at $p<.05$, suggesting randomization produced initially equivalent groups.
All 597 participants completed the T1 assessment, 586 (98%) completed T2, and 582 (98%) completed T3. Participants who completed all three assessments (n=590, 97%) were compared to those who did not (n=17, 3%) on study condition, demographic characteristics, baseline outcomes, and baseline back pain measures. No statistically significant differences were found with the exception of the baseline intention score. Participants who did not complete all three assessments had significantly (t-value=2.22, p=.049) lower intention scores than those who did complete all assessments (3.45 vs. 4.07, respectively). However, mean differences were associated with a small effect size (Cohen’s d=.27; Cohen, 1988).
Missing Data
Rates of missing data ranged from 0–5% at T1, 2–5% at T2, and 3–8% at T3. Despite the low rates of missing data, one fully imputed data set was generated for this intent-to-treat analysis as it produces less bias then other missing data techniques, such as list-wise deletion and last observation carried forward (Shafer and Graham, 2002). Missing data were imputed using IVEWare (Raghunathan, Solenberger, & Van Hoewyk, 2002), which uses all available data to impute missing data via a sequential regression approach. The observed and imputed data were compared to ensure they showed similar distributions (Abayomi, Gelman, & Levy, 2008).
Analytic Models
Primary analysis included analysis of covariance (ANCOVA) models to test for group differences on study outcomes at T2 and T3, separately, with the T1 score as a covariate and study condition as a three-level predictor (1=treatment, 2=alternative care, 3=control). If the overall omnibus test was significant, then follow-up planned contrasts (treatment vs. alternative care and treatment vs. control) were examined. Eta-square is provided as a measure of effects size with the convention .01 small, .06 moderate, and .14 large (Cohen, 1988). Next, the moderating effects of gender and age were tested at T2 and T3 by entering the moderator and the moderator by study condition interaction term. A Benjamini-Hochberg false discovery rate correction (Benjamini, & Hochberg 1995) was made to overall tests of main effects, planned contrasts, and moderating models to help protect against Type-I errors.
Secondary analysis included examination of the survey item that ascertained whether or not a study participant was currently experiencing back pain (“yes” or “no” response option). Logistic regression models were used to determine whether study condition predicted current back pain status at T2 and T3, separately, with the T1 response as a covariate. A within-subject dose response analysis was also performed with the FitBack intervention participants to test the hypothesis that those participants who used the program more showed greater gains in the study outcomes. Total time on the site was obtained from the log files and correlated with change scores (T2 minus T1 scores and T3 minus T1 scores). Finally, independent t-tests were used to compare the FitBack participants and alternative care participants on four program satisfaction items administered as part of the T2 and T3 surveys.

12b) CONSORT: Methods for additional analyses, such as subgroup analyses and adjusted analyses

Statistical Methods
Preliminary Analysis
Chi-square tests and one-way analysis of variance models were used to compare the three groups on the demographic characteristics, baseline outcomes, and baseline back pain measures. None were found to be statistically significant at $p<.05$, suggesting randomization produced initially equivalent groups.
All 597 participants completed the T1 assessment, 586 (98%) completed T2, and 582 (98%) completed T3. Participants who completed all three assessments (n=590, 97%) were compared to those who did not (n=17, 3%) on study condition, demographic characteristics, baseline outcomes, and baseline back pain measures. No statistically significant differences were found with the exception of the baseline intention score. Participants who did not complete all three assessments had significantly (t-value=2.22, p=.049) lower intention scores than those who did complete all assessments (3.45 vs. 4.07, respectively). However, mean differences were associated with a small effect size (Cohen's d=.27; Cohen, 1988).
Missing Data
Rates of missing data ranged from 0–5% at T1, 2–5% at T2, and 3–8% at T3. Despite the low rates of missing data, one fully imputed data set was generated for this intent-to-treat analysis as it produces less bias then other missing data techniques, such as list-wise deletion and last observation carried forward (Shafer and Graham, 2002). Missing data were imputed using IVEWare (Raghunathan, Solenberger, & Van Hoewyk, 2002), which uses all available data to impute missing data via a sequential regression approach. The observed and imputed data were compared to ensure they showed similar distributions (Abayomi, Gelman, & Levy, 2008).
Analytic Models
Primary analysis included analysis of covariance (ANCOVA) models to test for group differences on study outcomes at T2 and T3, separately, with the T1 score as a covariate and study condition as a three-level predictor (1=treatment, 2=alternative care, 3=control). If the overall omnibus test was significant, then follow-up planned contrasts (treatment vs. alternative care and treatment vs. control) were examined. Eta-square is provided as a measure of effects size with the convention .01 small, .06 moderate, and .14 large (Cohen, 1988). Next, the moderating effects of gender and age were tested at T2 and T3 by entering the moderator and the moderator by study condition interaction term. A Benjamini-Hochberg false discovery rate correction (Benjamini, & Hochberg 1995) was made to overall tests of main effects, planned contrasts, and moderating models to help protect against Type-I errors.
Secondary analysis included examination of the survey item that ascertained whether or not a study participant was currently experiencing back pain ("yes" or "no" response option). Logistic regression models were used to determine whether study condition predicted current back pain status at T2 and T3, separately, with the T1 response as a covariate. A within-subject dose response analysis was also performed with the FitBack intervention participants to test the hypothesis that those participants who used the program more showed greater gains in the study outcomes. Total time on the site was obtained from the log files and correlated with change scores (T2 minus T1 scores and T3 minus T1 scores). Finally, independent t-tests were used to compare the FitBack participants and alternative care participants on four program satisfaction items administered as part of the T2 and T3 surveys.

RESULTS

13a) CONSORT: For each group, the numbers of participants who were randomly assigned, received intended treatment, and were analysed for the primary outcome

Results

Participants

Participants were 597 workers recruited from our worksite partner (n=244) and the general work population (n=353). Worker job types and other demographic information is shown in Table 1. About half of the participants (51%) indicated they currently had low back pain. Chi-square statistics and associated p-values show groups did not differ on demographic characteristics.

The pain participants reported is described in Table 2 along with the causes to which they ascribed their pain. Table 2 shows the history of back pain items by each of the three study conditions. Chi-square tests were computed to compare groups on all items; no statistically significant differences were found.

Analyses

Primary Analysis

Table 3 provides means and standard deviations for all outcomes at each time point across all three study conditions, and Table 4 provides the results of the ANCOVA models testing for group differences at the T2 and T3 assessments, including effect size measurements. Seven of the twelve omnibus tests at T2 (prevention/helping behaviors, back pain knowledge, functionality and quality of life, PAM, self-efficacy, intentions, and SOPA control scale) and eight of the twelve omnibus tests at T3 (prevention/helping behaviors, back pain knowledge, functionality and quality of life, PAM, self-efficacy, intentions, SOPA control scale, and Dartmouth COOP) were statistically significant and warranted follow-up comparisons.

All Tx vs. Ctrl follow-up comparisons were statistically significant at T2 and at T3. The T2 comparisons showed five medium effects ($\eta2$=.07–.10) and two small effects ($\eta2$=. 02–.05). The T3 comparisons showed two large effects (both $\eta2$=.15), four medium effects (PAM, $\eta2$=.09–.12) and one small effect ($\eta2$=.03).

Tx vs. AltCtrl showed only three of the seven T2 follow-up comparisons to be statistically significant, and all were associated with small effect sizes ($\eta2$=.01–.02). For the T3 follow-up comparisons, seven of the eight comparisons were statistically significant with small effects sizes ($\eta2$=.02–.05). Examination of observed means shows that in all comparisons, the Tx group showed more favorable mean outcome scores than the AltCtrl group.

Taken together, results of the main effects analyses show that the Tx group outperformed the Ctrl group on most of the study outcome measures at both follow-up assessments. The average effect sizes were $\eta2$=.07 at T2 and $\eta2$=.10 at T3. These results indicate that the intervention effects increased in magnitude through the 4-month follow-up. Results were less robust when compared to the AltCtrl group, but nonetheless, the Tx group did show small positive changes on most of the study outcomes at the 4-month follow-up assessment.

Results of the moderation analysis by gender and age showed no significant condition by sex and age interaction terms at the T2 or T2 assessments. Thus, the FitBack program worked equally well for male and female participants and age was not a factor.

Secondary Analyses

Rates of current back pain were 48%, 54%, and 50% for the Tx, AltCtrl, and Ctrl participants, respectively, at T1 ($\chi2$[2,597]=1.78, p=.410); 42%, 46%, and 49% at T2 ($\chi2$[2,597]=2.00, p=.368); and 29%, 41%, and 41% at T3 ($\chi2$[2,597]=7.61, p=.022). Two contrasts were created: Tx (=0) vs. Ctrl (=1) and Tx (=0) vs. AltCtrl (=1). Logistic regression models were run with the contrasts as the outcomes, with T2 and T3 current back pain scores as predictors, while controlling for T1 current back pain score. Current adjusted back pain status at T2 was not a statistically significant predictor of either contrast. At T3, however, current adjusted back pain status was a significant predictor for both the Tx vs. Ctrl (OR=1.72, 95% CI=1.11-2.68, p=.016) and Tx vs. AltCtrl (OR=1.60, 95% CI=1.03-2.50, p=.035) contrasts. If a study participant reported current back pain at T3, s/he was approximately 1.6 times more likely to be in the AltCtrl group and 1.7 times more likely to be in the Ctrl group than to be in the FitBack Tx group; both odds ratios are associated with small to medium effects.

Examination of log files showed the average total number of minutes spent using the FitBack program by Tx participants was 29.1 (SD=41.8, minimum=0, maximum=341.9). The number of visits to the site ranged from 0 to 87 (M=4.9; SD=4.2). The total time on the site was correlated with T2–T1 and T3–T1 change scores for each of the study outcomes. At T2, more time using the site was significantly correlated with four of the twelve change scores: prevention helping behaviors (r=.15, p=.033), knowledge (r=.21, p=.003), PAM (r=.16, p=.028), and SOPA emotion subscale (r=.18, p=.013). At T3, more time spent using the site was significantly correlated with five of the twelve change scores: knowledge (r=.20, p=.005), self-efficacy (r=.14, p=.043), SOPA control scale (r=.14, p=.044), WLQ productivity (r=.14, p=.043), and the Dartmouth COOP (r=-.15, p=.034). All correlations were associated with small/medium effects sizes and in the hypothesized direction: more time spent using the site resulted in more favorable change in study outcomes. Comparable time of use data for the AltCtrl participants was not obtainable because these participants received a list of on-line back pain resources embedded in their initial and reminder emails. Click rates recorded by the email host indicated that participants clicked to open 75.6% of the initial emails with that percentage decreasing to 55.8 % of the last reminders. The click rate on one of the included links varied from 29.3% and 31.2% in reminder emails 1 and 2 respectively, to 12.6% in reminder 8. The most popular websites were WebMD Back Pain Health Center (104 clicks), Medline Plus Back Pain (100 clicks), Mayo Clinic (98 clicks), and American Academy of Orthopedic Surgeons (98 clicks).

Indices of user acceptance were all positive. Compared to the AltCtrl participants, FitBack program users had higher satisfaction ratings. The mean total score for FitBack participants was statistically greater at T2 (t[380]=4.40, p<.001, d=.54) and T3 (t[382]=3.51, p<.001, d=.37). The SUS score (M=78.6, SD=15.7), when compared to normative data, is associated with “good” to “excellent” ratings and corresponds to a “B–” (Bangor et al., 2009). For comparison, across 3,500 surveys within 273 studies on different platforms (web, cell phones, TV, etc.), the average SUS score was approximately 70. For web applications, the average SUS score was 68.2 (Bangor et al., 2009). The Perception of Employer Survey results suggest that participants believed they would have a positive impression of employers who made the FitBack program available to employees. They felt that the company would care about them (M=4.7; SD=1.1); they would feel more positive about the company (M=4.6; SD=1.1); they would have greater commitment to the company (M=4.1; SD=1.2); they would be more productive (M=4.1; SD=1.2); and they would feel more job satisfaction (M=4.0; SD=1.3). Results from the Understanding and Implementation Survey indicate that 96%–98% of participants thought they understood the program recommendations for use of heat and ice, over-the-counter medications, exercising to deal with back pain, and relaxation techniques. Implementation of recommendations by participants was reported for relaxation activities (67.8%) and exercises for prevention (78.2%) and dealing with pain occurrences (86%).

13b) CONSORT: For each group, losses and exclusions after randomisation, together with reasons

Results
Participants
Participants were 597 workers recruited from our worksite partner (n=244) and the general work population (n=353). Worker job types and other demographic information is shown in Table 1. About half of the participants (51%) indicated they currently had low back pain. Chi-square statistics and associated p-values show groups did not differ on demographic characteristics.
The pain participants reported is described in Table 2 along with the causes to which they ascribed their pain. Table 2 shows the history of back pain items by each of the three study conditions. Chi-square tests were computed to compare groups on all items; no statistically significant differences were found.
Analyses
Primary Analysis
Table 3 provides means and standard deviations for all outcomes at each time point across all three study conditions, and Table 4 provides the results of the ANCOVA models testing for group differences at the T2 and T3 assessments, including effect size measurements. Seven of the twelve omnibus tests at T2 (prevention/helping behaviors, back pain knowledge, functionality and quality of life, PAM, self-efficacy, intentions, and SOPA control scale) and eight of the twelve omnibus tests at T3 (prevention/helping behaviors, back pain knowledge, functionality and quality of life, PAM, self-efficacy, intentions, SOPA control scale, and Dartmouth COOP) were statistically significant and warranted follow-up comparisons.
All Tx vs. Ctrl follow-up comparisons were statistically significant at T2 and at T3. The T2 comparisons showed five medium effects (η^2=.07–.10) and two small effects (η^2=. 02–.05). The T3 comparisons showed two large effects (both η^2=.15), four medium effects (PAM, η^2=.09–.12) and one small effect (η^2=.03).
Tx vs. AltCtrl showed only three of the seven T2 follow-up comparisons to be statistically significant, and all were associated with small effect sizes (η^2=.01–.02). For the T3 follow-up comparisons, seven of the eight comparisons were statistically significant with small effects sizes (η^2=.02–.05). Examination of observed means shows that in all comparisons, the Tx group showed more favorable mean outcome scores than the AltCtrl group.
Taken together, results of the main effects analyses show that the Tx group outperformed the Ctrl group on most of the study outcome measures at both follow-up assessments. The average effect sizes were η^2=.07 at T2 and η^2=.10 at T3. These results indicate that the intervention effects increased in magnitude through the 4-month follow-up. Results were less robust when compared to the AltCtrl group, but nonetheless, the Tx group did show small positive changes on most of the study outcomes at the 4-month follow-up assessment.
Results of the moderation analysis by gender and age showed no significant condition by sex and age interaction terms at the T2 or T2 assessments. Thus, the FitBack program worked equally well for male and female participants and age was not a factor.
Secondary Analyses
Rates of current back pain were 48%, 54%, and 50% for the Tx, AltCtrl, and Ctrl participants, respectively, at T1 (χ^2[2,597]=1.78, p=.410); 42%, 46%, and 49% at T2 (χ^2[2,597]=2.00, p=.368); and 29%, 41%, and 41% at T3 (χ^2[2,597]=7.61, p=.022). Two contrasts were created: Tx (=0) vs. Ctrl (=1) and Tx (=0) vs. AltCtrl (=1). Logistic regression models were run with the contrasts as the outcomes, with T2 and T3 current back pain scores as predictors, while controlling for T1 current back pain score. Current adjusted back pain status at T2 was not a statistically significant predictor of either contrast. At T3, however, current adjusted back pain status was a significant predictor for both the Tx vs. Ctrl (OR=1.72, 95% CI=1.11-2.68, p=.016) and Tx vs. AltCtrl (OR=1.60, 95% CI=1.03-2.50, p=.035) contrasts. If a study participant reported current back pain at T3, s/he was approximately 1.6 times more likely to be in the AltCtrl group and 1.7 times more likely to be in the Ctrl group than to be in the FitBack Tx group; both odds ratios are associated with small to medium effects.
Examination of log files showed the average total number of minutes spent using the FitBack program by Tx participants was 29.1 (SD=41.8, minimum=0, maximum=341.9). The number of visits to the site ranged from 0 to 87 (M=4.9; SD=4.2). The total time on the site was correlated with T2–T1 and T3–T1 change scores for each of the study outcomes. At T2, more time using the site was significantly correlated with four of the twelve change scores: prevention helping behaviors (r=.15, p=.033), knowledge (r=.21, p=.003), PAM (r=.16, p=.028), and SOPA emotion subscale (r=.18, p=.013). At T3, more time spent using the site was significantly correlated with five of the twelve change scores: knowledge (r=.20, p=.005), self-efficacy (r=.14, p=. 043), SOPA control scale (r=.14, p=.044), WLQ productivity (r=.14, p=.043), and the Dartmouth COOP (r=-.15, p=.034). All correlations were associated with small/medium effects sizes and in the hypothesized direction: more time spent using the site resulted in more favorable change in study outcomes.
Comparable time of use data for the AltCtrl participants was not obtainable because these participants received a list of on-line back pain resources embedded in their initial and reminder emails. Click rates recorded by the email host indicated that participants clicked to open 75.6% of the initial emails with that percentage decreasing to 55.8 % of the last reminders. The click rate on one of the included links varied from 29.3% and 31.2% in reminder emails 1 and 2 respectively, to 12.6% in reminder 8. The most popular websites were WebMD Back Pain Health Center (104 clicks), Medline Plus Back Pain (100 clicks), Mayo Clinic (98 clicks), and American Academy of Orthopedic Surgeons (98 clicks).
Indices of user acceptance were all positive. Compared to the AltCtrl participants, FitBack program users had higher satisfaction ratings. The mean total score for FitBack participants was statistically greater at T2 (t[380]=4.40, p<.001, d=.54) and T3 (t[382]=3.51, p<.001, d=.37). The SUS score (M=78.6, SD=15.7), when compared to normative data, is associated with "good" to "excellent" ratings and corresponds to a "B–" (Bangor et al., 2009). For comparison, across 3,500 surveys within 273 studies on different platforms (web, cell phones, TV, etc.), the average SUS score was approximately 70. For web applications, the average SUS score was 68.2 (Bangor et al., 2009). The Perception of Employer Survey results suggest that participants believed they would have a positive impression of employers who made the FitBack program available to employees. They felt that the company would care about them (M=4.7; SD=1.1); they would feel more positive about the company (M=4.6; SD=1.1); they would have greater commitment to the company (M=4.1; SD=1.2); they would be more productive (M=4.1; SD=1.2); and they would feel more job satisfaction (M=4.0; SD=1.3). Results from the Understanding and Implementation Survey indicate that 96%–98% of participants thought they understood the program recommendations for use of heat and ice, over-the-counter medications, exercising to deal with back pain, and relaxation techniques. Implementation of recommendations by participants was reported for relaxation activities (67.8%) and exercises for prevention (78.2%) and dealing with pain occurrences (86%).
13b-i) Attrition diagram

Results
Participants
Participants were 597 workers recruited from our worksite partner (n=244) and the general work population (n=353). Worker job types and other demographic information is shown in Table 1. About half of the participants (51%) indicated they currently had low back pain. Chi-square statistics and associated p-values show groups did not differ on demographic characteristics.
The pain participants reported is described in Table 2 along with the causes to which they ascribed their pain. Table 2 shows the history of back pain items by each of the three study conditions. Chi-square tests were computed to compare groups on all items; no statistically significant differences were found.
Analyses
Primary Analysis
Table 3 provides means and standard deviations for all outcomes at each time point across all three study conditions, and Table 4 provides the results of the ANCOVA models testing for group differences at the T2 and T3 assessments, including effect size measurements. Seven of the twelve omnibus tests at T2 (prevention/helping behaviors, back pain knowledge, functionality and quality of life, PAM, self-efficacy, intentions, and SOPA control scale) and eight of the twelve omnibus tests at T3 (prevention/helping behaviors, back pain knowledge, functionality and quality of life, PAM, self-efficacy, intentions, SOPA control scale, and Dartmouth COOP) were statistically significant and warranted follow-up comparisons.
All Tx vs. Ctrl follow-up comparisons were statistically significant at T2 and at T3. The T2 comparisons showed five medium effects (η^2=.07–.10) and two small effects (η^2=. 02–.05). The T3 comparisons showed two large effects (both η^2=.15), four medium effects (PAM, η^2=.09–.12) and one small effect (η^2=.03).
Tx vs. AltCtrl showed only three of the seven T2 follow-up comparisons to be statistically significant, and all were associated with small effect sizes (η^2=.01–.02). For the T3 follow-up comparisons, seven of the eight comparisons were statistically significant with small effects sizes (η^2=.02–.05). Examination of observed means shows that in all comparisons, the Tx group showed more favorable mean outcome scores than the AltCtrl group.
Taken together, results of the main effects analyses show that the Tx group outperformed the Ctrl group on most of the study outcome measures at both follow-up assessments. The average effect sizes were η^2=.07 at T2 and η^2=.10 at T3. These results indicate that the intervention effects increased in magnitude through the 4-month follow-up. Results were less robust when compared to the AltCtrl group, but nonetheless, the Tx group did show small positive changes on most of the study outcomes at the 4-month follow-up assessment.
Results of the moderation analysis by gender and age showed no significant condition by sex and age interaction terms at the T2 or T2 assessments. Thus, the FitBack program worked equally well for male and female participants and age was not a factor.
Secondary Analyses
Rates of current back pain were 48%, 54%, and 50% for the Tx, AltCtrl, and Ctrl participants, respectively, at T1 (χ^2[2,597]=1.78, p=.410); 42%, 46%, and 49% at T2 (χ^2[2,597]=2.00, p=.368); and 29%, 41%, and 41% at T3 (χ^2[2,597]=7.61, p=.022). Two contrasts were created: Tx (=0) vs. Ctrl (=1) and Tx (=0) vs. AltCtrl (=1). Logistic regression models were run with the contrasts as the outcomes, with T2 and T3 current back pain scores as predictors, while controlling for T1 current back pain score. Current adjusted back pain status at T2 was not a statistically significant predictor of either contrast. At T3, however, current adjusted back pain status was a significant predictor for both the Tx vs. Ctrl (OR=1.72, 95% CI=1.11-2.68, p=.016) and Tx vs. AltCtrl (OR=1.60, 95% CI=1.03-2.50, p=.035) contrasts. If a study participant reported current back pain at T3, s/he was approximately 1.6 times more likely to be in the AltCtrl group and 1.7 times more likely to be in the Ctrl group than to be in the FitBack Tx group; both odds ratios are associated with small to medium effects.
Examination of log files showed the average total number of minutes spent using the FitBack program by Tx participants was 29.1 (SD=41.8, minimum=0, maximum=341.9). The number of visits to the site ranged from 0 to 87 (M=4.9; SD=4.2). The total time on the site was correlated with T2–T1 and T3–T1 change scores for each of the study outcomes. At T2, more time using the site was significantly correlated with four of the twelve change scores: prevention helping behaviors (r=.15, p=.033), knowledge (r=.21, p=.003), PAM (r=.16, p=.028), and SOPA emotion subscale (r=.18, p=.013). At T3, more time spent using the site was significantly correlated with five of the twelve change scores: knowledge (r=.20, p=.005), self-efficacy (r=.14, p=. 043), SOPA control scale (r=.14, p=.044), WLQ productivity (r=.14, p=.043), and the Dartmouth COOP (r=-.15, p=.034). All correlations were associated with small/medium effects sizes and in the hypothesized direction: more time spent using the site resulted in more favorable change in study outcomes. Comparable time of use data for the AltCtrl participants was not obtainable because these participants received a list of on-line back pain resources embedded in their initial and reminder emails. Click rates recorded by the email host indicated that participants clicked to open 75.6% of the initial emails with that percentage decreasing to 55.8 % of the last reminders. The click rate on one of the included links varied from 29.3% and 31.2% in reminder emails 1 and 2 respectively, to 12.6% in reminder 8. The most popular websites were WebMD Back Pain Health Center (104 clicks), Medline Plus Back Pain (100 clicks), Mayo Clinic (98 clicks), and American Academy of Orthopedic Surgeons (98 clicks).
Indices of user acceptance were all positive. Compared to the AltCtrl participants, FitBack program users had higher satisfaction ratings. The mean total score for FitBack participants was statistically greater at T2 (t[380]=4.40, p<.001, d=.54) and T3 (t[382]=3.51, p<.001, d=.37). The SUS score (M=78.6, SD=15.7), when compared to normative data, is associated with “good” to “excellent” ratings and corresponds to a “B–” (Bangor et al., 2009). For comparison, across 3,500 surveys within 273 studies on different platforms (web, cell phones, TV, etc.), the average SUS score was approximately 70. For web applications, the average SUS score was 68.2 (Bangor et al., 2009). The Perception of Employer Survey results suggest that participants believed they would have a positive impression of employers who made the FitBack program available to employees. They felt that the company would care about them (M=4.7; SD=1.1); they would feel more positive about the company (M=4.6; SD=1.1); they would have greater commitment to the company (M=4.1; SD=1.2); they would be more productive (M=4.1; SD=1.2); and they would feel more job satisfaction (M=4.0; SD=1.3). Results from the Understanding and Implementation Survey indicate that 96%–98% of participants thought they understood the program recommendations for use of heat and ice, over-the-counter medications, exercising to deal with back pain, and relaxation techniques. Implementation of recommendations by participants was reported for relaxation activities (67.8%) and exercises for prevention (78.2%) and dealing with pain occurrences (86%).
14a) CONSORT: Dates defining the periods of recruitment and follow-up
NA
14a-i) Indicate if critical “secular events” fell into the study period
na
14b) CONSORT: Why the trial ended or was stopped (early)
na
15) CONSORT: A table showing baseline demographic and clinical characteristics for each group

Table 1. Study Demographic Characteristics.
Treatment (n=199)Alternate Care (n=199)Control (n=199)
n%n%n%χ2p
Female11658.311758.812562.81.02.600
Hispanic/Latino2010.12412.12311.60.44.804
Race10.31.413
American Indian/Alaskan Native21.010.510.5
Asian147.0105.0115.5
Black or African American168.0178.5105.0
White or Caucasian15276.415879.416381.9
Multiracial126.063.052.5
Other/unknown31.573.594.5
Marital status11.82.159
Married or living with a partner12663.313969.814472.4
Divorced2010.1157.52311.6
Widowed10.521.000.0
Separated73.531.563.0
Single4522.64020.12613.1
Highest level of education8.77.362
Less than high school graduate21.000.021.0
High school graduate/GED2412.1168.0136.5
Some college5728.66432.07537.7
College degree (BA, BS, BFA)7537.77839.27636.7
Graduate school (MA, MS, PhD)4120.64120.63618.1
Annual household income11.70.306
Less than $20,000105.094.5136.5
$20,000 - $39,999 3819.12814.13618.1
$40,000 - $59,9994522.63819.15226.1
$60,000 - $79,9993718.63417.12412.1
$80,000 - $99,9992613.12914.62914.6
More than $100,0004321.66130.74522.6
Employment status12.75.388
Full time14472.414371.914371.9
Part time2814.13115.63015.1
Retired31.510.500.0
Volunteer31.500.021.0
Homemaker157.5178.5178.5
Unemployed10.563.042.0
Non-working student52.510.531.5
Worker classification1.82.936
Sitter12462.312462.312462.3
Stander5025.15125.64924.6
Lifter2211.12311.62211.1
Driver31.510.542.0

15-i) Report demographics associated with digital divide issues

Table 1. Study Demographic Characteristics.
Treatment (n=199)Alternate Care (n=199)Control (n=199)
n%n%n%χ2p
Female11658.311758.812562.81.02.600
Hispanic/Latino2010.12412.12311.60.44.804
Race10.31.413
American Indian/Alaskan Native21.010.510.5
Asian147.0105.0115.5
Black or African American168.0178.5105.0
White or Caucasian15276.415879.416381.9
Multiracial126.063.052.5
Other/unknown31.573.594.5
Marital status11.82.159
Married or living with a partner12663.313969.814472.4
Divorced2010.1157.52311.6
Widowed10.521.000.0
Separated73.531.563.0
Single4522.64020.12613.1
Highest level of education8.77.362
Less than high school graduate21.000.021.0
High school graduate/GED2412.1168.0136.5
Some college5728.66432.07537.7
College degree (BA, BS, BFA)7537.77839.27636.7
Graduate school (MA, MS, PhD)4120.64120.63618.1
Annual household income11.70.306
Less than $20,000105.094.5136.5
$20,000 - $39,999 3819.12814.13618.1
$40,000 - $59,9994522.63819.15226.1
$60,000 - $79,9993718.63417.12412.1
$80,000 - $99,9992613.12914.62914.6
More than $100,0004321.66130.74522.6
Employment status12.75.388
Full time14472.414371.914371.9
Part time2814.13115.63015.1
Retired31.510.500.0
Volunteer31.500.021.0
Homemaker157.5178.5178.5
Unemployed10.563.042.0
Non-working student52.510.531.5
Worker classification1.82.936
Sitter12462.312462.312462.3
Stander5025.15125.64924.6
Lifter2211.12311.62211.1
Driver31.510.542.0

16a) CONSORT: For each group, number of participants (denominator) included in each analysis and whether the analysis was by original assigned groups

16-i) Report multiple “denominators” and provide definitions

Results

Participants

Participants were 597 workers recruited from our worksite partner (n=244) and the general work population (n=353). Worker job types and other demographic information is shown in Table 1. About half of the participants (51%) indicated they currently had low back pain. Chi-square statistics and associated p-values show groups did not differ on demographic characteristics.

The pain participants reported is described in Table 2 along with the causes to which they ascribed their pain. Table 2 shows the history of back pain items by each of the three study conditions. Chi-square tests were computed to compare groups on all items; no statistically significant differences were found.

Analyses

Primary Analysis

Table 3 provides means and standard deviations for all outcomes at each time point across all three study conditions, and Table 4 provides the results of the ANCOVA models testing for group differences at the T2 and T3 assessments, including effect size measurements. Seven of the twelve omnibus tests at T2 (prevention/helping behaviors, back pain knowledge, functionality and quality of life, PAM, self-efficacy, intentions, and SOPA control scale) and eight of the twelve omnibus tests at T3 (prevention/helping behaviors, back pain knowledge, functionality and quality of life, PAM, self-efficacy, intentions, SOPA control scale, and Dartmouth COOP) were statistically significant and warranted follow-up comparisons.

All Tx vs. Ctrl follow-up comparisons were statistically significant at T2 and at T3. The T2 comparisons showed five medium effects (η^2=.07–.10) and two small effects (η^2=. 02–.05). The T3 comparisons showed two large effects (both η^2=.15), four medium effects (PAM, η^2=.09–.12) and one small effect (η^2=.03).

Tx vs. AltCtrl showed only three of the seven T2 follow-up comparisons to be statistically significant, and all were associated with small effect sizes (η^2=.01–.02). For the T3 follow-up comparisons, seven of the eight comparisons were statistically significant with small effects sizes (η^2=.02–.05). Examination of observed means shows that in all comparisons, the Tx group showed more favorable mean outcome scores than the AltCtrl group.

Taken together, results of the main effects analyses show that the Tx group outperformed the Ctrl group on most of the study outcome measures at both follow-up assessments. The average effect sizes were η^2=.07 at T2 and η^2=.10 at T3. These results indicate that the intervention effects increased in magnitude through the 4-month follow-up. Results were less robust when compared to the AltCtrl group, but nonetheless, the Tx group did show small positive changes on most of the study outcomes at the 4-month follow-up assessment.

Results of the moderation analysis by gender and age showed no significant condition by sex and age interaction terms at the T2 or T2 assessments. Thus, the FitBack program worked equally well for male and female participants and age was not a factor.

Secondary Analyses

Rates of current back pain were 48%, 54%, and 50% for the Tx, AltCtrl, and Ctrl participants, respectively, at T1 (χ^2[2,597]=1.78, p=.410); 42%, 46%, and 49% at T2 (χ^2[2,597]=2.00, p=.368); and 29%, 41%, and 41% at T3 (χ^2[2,597]=7.61, p=.022). Two contrasts were created: Tx (=0) vs. Ctrl (=1) and Tx (=0) vs. AltCtrl (=1). Logistic regression models were run with the contrasts as the outcomes, with T2 and T3 current back pain scores as predictors, while controlling for T1 current back pain score. Current adjusted back pain status at T2 was not a statistically significant predictor of either contrast. At T3, however, current adjusted back pain status was a significant predictor for both the Tx vs. Ctrl (OR=1.72, 95% CI=1.11-2.68, p=.016) and Tx vs. AltCtrl (OR=1.60, 95% CI=1.03-2.50, p=.035) contrasts. If a study participant reported current back pain at T3, s/he was approximately 1.6 times more likely to be in the AltCtrl group and 1.7 times more likely to be in the Ctrl group than to be in the FitBack Tx group; both odds ratios are associated with small to medium effects.

Examination of log files showed the average total number of minutes spent using the FitBack program by Tx participants was 29.1 (SD=41.8, minimum=0, maximum=341.9). The number of visits to the site ranged from 0 to 87 (M=4.9; SD=4.2). The total time on the site was correlated with T2–T1 and T3–T1 change scores for each of the study outcomes. At T2, more time using the site was significantly correlated with four of the twelve change scores: prevention helping behaviors (r=.15, p=.033), knowledge (r=.21, p=.003), PAM (r=.16, p=.028), and SOPA emotion subscale (r=.18, p=.013). At T3, more time spent using the site was significantly correlated with five of the twelve change scores: knowledge (r=.20, p=.005), self-efficacy (r=.14, p=. 043), SOPA control scale (r=.14, p=.044), WLQ productivity (r=.14, p=.043), and the Dartmouth COOP (r=-.15, p=.034). All correlations were associated with small/medium effects sizes and in the hypothesized direction: more time spent using the site resulted in more favorable change in study outcomes. Comparable time of use data for the AltCtrl participants was not obtainable because these participants received a list of on-line back pain resources embedded in their initial and reminder emails. Click rates recorded by the email host indicated that participants clicked to open 75.6% of the initial emails with that percentage decreasing to 55.8 % of the last reminders. The click rate on one of the included links varied from 29.3% and 31.2% in reminder emails 1 and 2 respectively, to 12.6% in reminder 8. The most popular websites were WebMD Back Pain Health Center (104 clicks), Medline Plus Back Pain (100 clicks), Mayo Clinic (98 clicks), and American Academy of Orthopedic Surgeons (98 clicks).

Indices of user acceptance were all positive. Compared to the AltCtrl participants, FitBack program users had higher satisfaction ratings. The mean total score for FitBack participants was statistically greater at T2 (t[380]=4.40, p<.001, d=.54) and T3 (t[382]=3.51, p<.001, d=.37). The SUS score (M=78.6, SD=15.7), when compared to normative data, is associated with “good” to “excellent” ratings and corresponds to a “B–” (Bangor et al., 2009). For comparison, across 3,500 surveys within 273 studies on different platforms (web, cell phones, TV, etc.), the average SUS score was approximately 70. For web applications, the average SUS score was 68.2 (Bangor et al., 2009). The Perception of Employer Survey results suggest that participants believed they would have a positive impression of employers who made the FitBack program available to employees. They felt that the company would care about them (M=4.7; SD=1.1); they would feel more positive about the company (M=4.6; SD=1.1); they would have greater commitment to the company (M=4.1; SD=1.2); they would be more productive (M=4.1; SD=1.2); and they would feel more job satisfaction (M=4.0; SD=1.3). Results from the Understanding and Implementation Survey indicate that 96%–98% of participants thought they understood the program recommendations for use of heat and ice, over-the-counter medications, exercising to deal with back pain, and relaxation techniques. Implementation of recommendations by participants was reported for relaxation activities (67.8%) and exercises for prevention (78.2%) and dealing with pain occurrences (86%).

16-ii) Primary analysis should be intent-to-treat

Results

Participants

Participants were 597 workers recruited from our worksite partner (n=244) and the general work population (n=353). Worker job types and other demographic information is shown in Table 1. About half of the participants (51%) indicated they currently had low back pain. Chi-square statistics and associated p-values show groups did not differ on demographic characteristics.

The pain participants reported is described in Table 2 along with the causes to which they ascribed their pain. Table 2 shows the history of back pain items by each of the three study conditions. Chi-square tests were computed to compare groups on all items; no statistically significant differences were found.

Analyses

Primary Analysis

Table 3 provides means and standard deviations for all outcomes at each time point across all three study conditions, and Table 4 provides the results of the ANCOVA models testing for group differences at the T2 and T3 assessments, including effect size measurements. Seven of the twelve omnibus tests at T2 (prevention/helping behaviors, back pain knowledge, functionality and quality of life, PAM, self-efficacy, intentions, and SOPA control scale) and eight of the twelve omnibus tests at T3 (prevention/helping behaviors, back pain knowledge, functionality and quality of life, PAM, self-efficacy, intentions, SOPA control scale, and Dartmouth COOP) were statistically significant and warranted follow-up comparisons.

All Tx vs. Ctrl follow-up comparisons were statistically significant at T2 and at T3. The T2 comparisons showed five medium effects (η^2=.07–.10) and two small effects (η^2=. 02–.05). The T3 comparisons showed two large effects (both η^2=.15), four medium effects (PAM, η^2=.09–.12) and one small effect (η^2=.03).

Tx vs. AltCtrl showed only three of the seven T2 follow-up comparisons to be statistically significant, and all were associated with small effect sizes (η^2=.01–.02). For the T3 follow-up comparisons, seven of the eight comparisons were statistically significant with small effects sizes (η^2=.02–.05). Examination of observed means shows that in all comparisons, the Tx group showed more favorable mean outcome scores than the AltCtrl group.

Taken together, results of the main effects analyses show that the Tx group outperformed the Ctrl group on most of the study outcome measures at both follow-up assessments. The average effect sizes were η^2=.07 at T2 and η^2=.10 at T3. These results indicate that the intervention effects increased in magnitude through the 4-month follow-up. Results were less robust when compared to the AltCtrl group, but nonetheless, the Tx group did show small positive changes on most of the study outcomes at the 4-month follow-up assessment.

Results of the moderation analysis by gender and age showed no significant condition by sex and age interaction terms at the T2 or T2 assessments. Thus, the FitBack program worked equally well for male and female participants and age was not a factor.

Secondary Analyses

Rates of current back pain were 48%, 54%, and 50% for the Tx, AltCtrl, and Ctrl participants, respectively, at T1 (χ^2[2,597]=1.78, p=.410); 42%, 46%, and 49% at T2 (χ^2[2,597]=2.00, p=.368); and 29%, 41%, and 41% at T3 (χ^2[2,597]=7.61, p=.022). Two contrasts were created: Tx (=0) vs. Ctrl (=1) and Tx (=0) vs. AltCtrl (=1). Logistic regression models were run with the contrasts as the outcomes, with T2 and T3 current back pain scores as predictors, while controlling for T1 current back pain score. Current adjusted back pain status at T2 was not a statistically significant predictor of either contrast. At T3, however, current adjusted back pain status was a significant predictor for both the Tx vs. Ctrl (OR=1.72, 95% CI=1.11-2.68, p=.016) and Tx vs. AltCtrl (OR=1.60, 95% CI=1.03-2.50, p=.035) contrasts. If a study participant reported current back pain at T3, s/he was approximately 1.6 times more likely to be in the AltCtrl group and 1.7 times more likely to be in the Ctrl group than to be in the FitBack Tx group; both odds ratios are associated with small to medium effects.

Examination of log files showed the average total number of minutes spent using the FitBack program by Tx participants was 29.1 (SD=41.8, minimum=0, maximum=341.9). The number of visits to the site ranged from 0 to 87 (M=4.9; SD=4.2). The total time on the site was correlated with T2–T1 and T3–T1 change scores for each of the study outcomes. At T2, more time using the site was significantly correlated with four of the twelve change scores: prevention helping behaviors (r=.15, p=.033), knowledge (r=.21, p=.003), PAM (r=.16, p=.028), and SOPA emotion subscale (r=.18, p=.013). At T3, more time spent using the site was significantly correlated with five of the twelve change scores: knowledge (r=.20, p=.005), self-efficacy (r=.14, p=. 043), SOPA control scale (r=.14, p=.044), WLQ productivity (r=.14, p=.043), and the Dartmouth COOP (r=-.15, p=.034). All correlations were associated with small/medium effects sizes and in the hypothesized direction: more time spent using the site resulted in more favorable change in study outcomes. Comparable time of use data for the AltCtrl participants was not obtainable because these participants received a list of on-line back pain resources embedded in their initial and reminder emails. Click rates recorded by the email host indicated that participants clicked to open 75.6% of the initial emails with that percentage decreasing to 55.8 % of the last reminders. The click rate on one of the included links varied from 29.3% and 31.2% in reminder emails 1 and 2 respectively, to 12.6% in reminder 8. The most popular websites were WebMD Back Pain Health Center (104 clicks), Medline Plus Back Pain (100 clicks), Mayo Clinic (98 clicks), and American Academy of Orthopedic Surgeons (98 clicks).

Indices of user acceptance were all positive. Compared to the AltCtrl participants, FitBack program users had higher satisfaction ratings. The mean total score for FitBack participants was statistically greater at T2 (t[380]=4.40, p<.001, d=.54) and T3 (t[382]=3.51, p<.001, d=.37). The SUS score (M=78.6, SD=15.7), when compared to normative data, is associated with "good" to "excellent" ratings and corresponds to a "B–" (Bangor et al., 2009). For comparison, across 3,500 surveys within 273 studies on different platforms (web, cell phones, TV, etc.), the average SUS score was approximately 70. For web applications, the average SUS score was 68.2 (Bangor et al., 2009). The Perception of Employer Survey results suggest that participants believed they would have a positive impression of employers who made the FitBack program available to employees. They felt that the company would care about them (M=4.7; SD=1.1); they would feel more positive about the company (M=4.6; SD=1.1); they would have greater commitment to the company (M=4.1; SD=1.2); they would be more productive (M=4.1; SD=1.2); and they would feel more job satisfaction (M=4.0; SD=1.3). Results from the Understanding and Implementation Survey indicate that 96%–98% of participants thought they understood the program recommendations for use of heat and ice, over-the-counter medications, exercising to deal with back pain, and relaxation techniques. Implementation of recommendations by participants was reported for relaxation activities (67.8%) and exercises for prevention (78.2%) and dealing with pain occurrences (86%).

17a) CONSORT: For each primary and secondary outcome, results for each group, and the estimated effect size and its precision (such as 95% confidence interval)

Results
Participants
Participants were 597 workers recruited from our worksite partner (n=244) and the general work population (n=353). Worker job types and other demographic information is shown in Table 1. About half of the participants (51%) indicated they currently had low back pain. Chi-square statistics and associated p-values show groups did not differ on demographic characteristics.
The pain participants reported is described in Table 2 along with the causes to which they ascribed their pain. Table 2 shows the history of back pain items by each of the three study conditions. Chi-square tests were computed to compare groups on all items; no statistically significant differences were found.
Analyses
Primary Analysis
Table 3 provides means and standard deviations for all outcomes at each time point across all three study conditions, and Table 4 provides the results of the ANCOVA models testing for group differences at the T2 and T3 assessments, including effect size measurements. Seven of the twelve omnibus tests at T2 (prevention/helping behaviors, back pain knowledge, functionality and quality of life, PAM, self-efficacy, intentions, and SOPA control scale) and eight of the twelve omnibus tests at T3 (prevention/helping behaviors, back pain knowledge, functionality and quality of life, PAM, self-efficacy, intentions, SOPA control scale, and Dartmouth COOP) were statistically significant and warranted follow-up comparisons.
All Tx vs. Ctrl follow-up comparisons were statistically significant at T2 and at T3. The T2 comparisons showed five medium effects ($\eta 2$=.07–.10) and two small effects ($\eta 2$=. 02–.05). The T3 comparisons showed two large effects (both $\eta 2$=.15), four medium effects (PAM, $\eta 2$=.09–.12) and one small effect ($\eta 2$=.03).
Tx vs. AltCtrl showed only three of the seven T2 follow-up comparisons to be statistically significant, and all were associated with small effect sizes ($\eta 2$=.01–.02). For the T3 follow-up comparisons, seven of the eight comparisons were statistically significant with small effects sizes ($\eta 2$=.02–.05). Examination of observed means shows that in all comparisons, the Tx group showed more favorable mean outcome scores than the AltCtrl group.
Taken together, results of the main effects analyses show that the Tx group outperformed the Ctrl group on most of the study outcome measures at both follow-up assessments. The average effect sizes were $\eta 2$=.07 at T2 and $\eta 2$=.10 at T3. These results indicate that the intervention effects increased in magnitude through the 4-month follow-up. Results were less robust when compared to the AltCtrl group, but nonetheless, the Tx group did show small positive changes on most of the study outcomes at the 4-month follow-up assessment.
Results of the moderation analysis by gender and age showed no significant condition by sex and age interaction terms at the T2 or T2 assessments. Thus, the FitBack program worked equally well for male and female participants and age was not a factor.
Secondary Analyses
Rates of current back pain were 48%, 54%, and 50% for the Tx, AltCtrl, and Ctrl participants, respectively, at T1 ($\chi 2[2,597]$=1.78, p=.410); 42%, 46%, and 49% at T2 ($\chi 2[2,597]$=2.00, p=.368); and 29%, 41%, and 41% at T3 ($\chi 2[2,597]$=7.61, p=.022). Two contrasts were created: Tx (=0) vs. Ctrl (=1) and Tx (=0) vs. AltCtrl (=1). Logistic regression models were run with the contrasts as the outcomes, with T2 and T3 current back pain scores as predictors, while controlling for T1 current back pain score. Current adjusted back pain status at T2 was not a statistically significant predictor of either contrast. At T3, however, current adjusted back pain status was a significant predictor for both the Tx vs. Ctrl (OR=1.72, 95% CI=1.11-2.68, p=.016) and Tx vs. AltCtrl (OR=1.60, 95% CI=1.03-2.50, p=.035) contrasts. If a study participant reported current back pain at T3, s/he was approximately 1.6 times more likely to be in the AltCtrl group and 1.7 times more likely to be in the Ctrl group than to be in the FitBack Tx group; both odds ratios are associated with small to medium effects.
Examination of log files showed the average total number of minutes spent using the FitBack program by Tx participants was 29.1 (SD=41.8, minimum=0, maximum=341.9). The number of visits to the site ranged from 0 to 87 (M=4.9; SD=4.2). The total time on the site was correlated with T2–T1 and T3–T1 change scores for each of the study outcomes. At T2, more time using the site was significantly correlated with four of the twelve change scores: prevention helping behaviors (r=.15, p=.033), knowledge (r=.21, p=.003), PAM (r=.16, p=.028), and SOPA emotion subscale (r=.18, p=.013). At T3, more time spent using the site was significantly correlated with five of the twelve change scores: knowledge (r=.20, p=.005), self-efficacy (r=.14, p=. 043), SOPA control scale (r=.14, p=.044), WLQ productivity (r=.14, p=.043), and the Dartmouth COOP (r=-.15, p=.034). All correlations were associated with small/medium effects sizes and in the hypothesized direction: more time spent using the site resulted in more favorable change in study outcomes.
Comparable time of use data for the AltCtrl participants was not obtainable because these participants received a list of on-line back pain resources embedded in their initial and reminder emails. Click rates recorded by the email host indicated that participants clicked to open 75.6% of the initial emails with that percentage decreasing to 55.8 % of the last reminders. The click rate on one of the included links varied from 29.3% and 31.2% in reminder emails 1 and 2 respectively, to 12.6% in reminder 8. The most popular websites were WebMD Back Pain Health Center (104 clicks), Medline Plus Back Pain (100 clicks), Mayo Clinic (98 clicks), and American Academy of Orthopedic Surgeons (98 clicks).
Indices of user acceptance were all positive. Compared to the AltCtrl participants, FitBack program users had higher satisfaction ratings. The mean total score for FitBack participants was statistically greater at T2 (t[380]=4.40, p<.001, d=.54) and T3 (t[382]=3.51, p<.001, d=.37). The SUS score (M=78.6, SD=15.7), when compared to normative data, is associated with “good” to “excellent” ratings and corresponds to a “B–” (Bangor et al., 2009). For comparison, across 3,500 surveys within 273 studies on different platforms (web, cell phones, TV, etc.), the average SUS score was approximately 70. For web applications, the average SUS score was 68.2 (Bangor et al., 2009). The Perception of Employer Survey results suggest that participants believed they would have a positive impression of employers who made the FitBack program available to employees. They felt that the company would care about them (M=4.7; SD=1.1); they would feel more positive about the company (M=4.6; SD=1.1); they would have greater commitment to the company (M=4.1; SD=1.2); they would be more productive (M=4.1; SD=1.2); and they would feel more job satisfaction (M=4.0; SD=1.3). Results from the Understanding and Implementation Survey indicate that 96%–98% of participants thought they understood the program recommendations for use of heat and ice, over-the-counter medications, exercising to deal with back pain, and relaxation techniques. Implementation of recommendations by participants was reported for relaxation activities (67.8%) and exercises for prevention (78.2%) and dealing with pain occurrences (86%).
17a-i) Presentation of process outcomes such as metrics of use and intensity of use

Results
Participants
Participants were 597 workers recruited from our worksite partner (n=244) and the general work population (n=353). Worker job types and other demographic information is shown in Table 1. About half of the participants (51%) indicated they currently had low back pain. Chi-square statistics and associated p-values show groups did not differ on demographic characteristics.
The pain participants reported is described in Table 2 along with the causes to which they ascribed their pain. Table 2 shows the history of back pain items by each of the three study conditions. Chi-square tests were computed to compare groups on all items; no statistically significant differences were found.
Analyses
Primary Analysis
Table 3 provides means and standard deviations for all outcomes at each time point across all three study conditions, and Table 4 provides the results of the ANCOVA models testing for group differences at the T2 and T3 assessments, including effect size measurements. Seven of the twelve omnibus tests at T2 (prevention/helping behaviors, back pain knowledge, functionality and quality of life, PAM, self-efficacy, intentions, and SOPA control scale) and eight of the twelve omnibus tests at T3 (prevention/helping behaviors, back pain knowledge, functionality and quality of life, PAM, self-efficacy, intentions, SOPA control scale, and Dartmouth COOP) were statistically significant and warranted follow-up comparisons.
All Tx vs. Ctrl follow-up comparisons were statistically significant at T2 and at T3. The T2 comparisons showed five medium effects (η^2=.07–.10) and two small effects (η^2=. 02–.05). The T3 comparisons showed two large effects (both η^2=.15), four medium effects (PAM, η^2=.09–.12) and one small effect (η^2=.03).
Tx vs. AltCtrl showed only three of the seven T2 follow-up comparisons to be statistically significant, and all were associated with small effect sizes (η^2=.01–.02). For the T3 follow-up comparisons, seven of the eight comparisons were statistically significant with small effects sizes (η^2=.02–.05). Examination of observed means shows that in all comparisons, the Tx group showed more favorable mean outcome scores than the AltCtrl group.
Taken together, results of the main effects analyses show that the Tx group outperformed the Ctrl group on most of the study outcome measures at both follow-up assessments. The average effect sizes were η^2=.07 at T2 and η^2=.10 at T3. These results indicate that the intervention effects increased in magnitude through the 4-month follow-up. Results were less robust when compared to the AltCtrl group, but nonetheless, the Tx group did show small positive changes on most of the study outcomes at the 4-month follow-up assessment.
Results of the moderation analysis by gender and age showed no significant condition by sex and age interaction terms at the T2 or T2 assessments. Thus, the FitBack program worked equally well for male and female participants and age was not a factor.
Secondary Analyses
Rates of current back pain were 48%, 54%, and 50% for the Tx, AltCtrl, and Ctrl participants, respectively, at T1 (χ^2[2,597]=1.78, p=.410); 42%, 46%, and 49% at T2 (χ^2[2,597]=2.00, p=.368); and 29%, 41%, and 41% at T3 (χ^2[2,597]=7.61, p=.022). Two contrasts were created: Tx (=0) vs. Ctrl (=1) and Tx (=0) vs. AltCtrl (=1). Logistic regression models were run with the contrasts as the outcomes, with T2 and T3 current back pain scores as predictors, while controlling for T1 current back pain score. Current adjusted back pain status at T2 was not a statistically significant predictor of either contrast. At T3, however, current adjusted back pain status was a significant predictor for both the Tx vs. Ctrl (OR=1.72, 95% CI=1.11-2.68, p=.016) and Tx vs. AltCtrl (OR=1.60, 95% CI=1.03-2.50, p=.035) contrasts. If a study participant reported current back pain at T3, s/he was approximately 1.6 times more likely to be in the AltCtrl group and 1.7 times more likely to be in the Ctrl group than to be in the FitBack Tx group; both odds ratios are associated with small to medium effects.
Examination of log files showed the average total number of minutes spent using the FitBack program by Tx participants was 29.1 (SD=41.8, minimum=0, maximum=341.9). The number of visits to the site ranged from 0 to 87 (M=4.9; SD=4.2). The total time on the site was correlated with T2–T1 and T3–T1 change scores for each of the study outcomes. At T2, more time using the site was significantly correlated with four of the twelve change scores: prevention helping behaviors (r=.15, p=.033), knowledge (r=.21, p=.003), PAM (r=.16, p=.028), and SOPA emotion subscale (r=.18, p=.013). At T3, more time spent using the site was significantly correlated with five of the twelve change scores: knowledge (r=.20, p=.005), self-efficacy (r=.14, p=. 043), SOPA control scale (r=.14, p=.044), WLQ productivity (r=.14, p=.043), and the Dartmouth COOP (r=-.15, p=.034). All correlations were associated with small/medium effects sizes and in the hypothesized direction: more time spent using the site resulted in more favorable change in study outcomes.
Comparable time of use data for the AltCtrl participants was not obtainable because these participants received a list of on-line back pain resources embedded in their initial and reminder emails. Click rates recorded by the email host indicated that participants clicked to open 75.6% of the initial emails with that percentage decreasing to 55.8 % of the last reminders. The click rate on one of the included links varied from 29.3% and 31.2% in reminder emails 1 and 2 respectively, to 12.6% in reminder 8. The most popular websites were WebMD Back Pain Health Center (104 clicks), Medline Plus Back Pain (100 clicks), Mayo Clinic (98 clicks), and American Academy of Orthopedic Surgeons (98 clicks).
Indices of user acceptance were all positive. Compared to the AltCtrl participants, FitBack program users had higher satisfaction ratings. The mean total score for FitBack participants was statistically greater at T2 (t[380]=4.40, p<.001, d=.54) and T3 (t[382]=3.51, p<.001, d=.37). The SUS score (M=78.6, SD=15.7), when compared to normative data, is associated with "good" to "excellent" ratings and corresponds to a "B–" (Bangor et al., 2009). For comparison, across 3,500 surveys within 273 studies on different platforms (web, cell phones, TV, etc.), the average SUS score was approximately 70. For web applications, the average SUS score was 68.2 (Bangor et al., 2009). The Perception of Employer Survey results suggest that participants believed they would have a positive impression of employers who made the FitBack program available to employees. They felt that the company would care about them (M=4.7; SD=1.1); they would feel more positive about the company (M=4.6; SD=1.1); they would have greater commitment to the company (M=4.1; SD=1.2); they would be more productive (M=4.1; SD=1.2); and they would feel more job satisfaction (M=4.0; SD=1.3). Results from the Understanding and Implementation Survey indicate that 96%–98% of participants thought they understood the program recommendations for use of heat and ice, over-the-counter medications, exercising to deal with back pain, and relaxation techniques. Implementation of recommendations by participants was reported for relaxation activities (67.8%) and exercises for prevention (78.2%) and dealing with pain occurrences (86%).
17b) CONSORT: For binary outcomes, presentation of both absolute and relative effect sizes is recommended

Results
Participants
Participants were 597 workers recruited from our worksite partner (n=244) and the general work population (n=353). Worker job types and other demographic information is shown in Table 1. About half of the participants (51%) indicated they currently had low back pain. Chi-square statistics and associated p-values show groups did not differ on demographic characteristics.
The pain participants reported is described in Table 2 along with the causes to which they ascribed their pain. Table 2 shows the history of back pain items by each of the three study conditions. Chi-square tests were computed to compare groups on all items; no statistically significant differences were found.
Analyses
Primary Analysis
Table 3 provides means and standard deviations for all outcomes at each time point across all three study conditions, and Table 4 provides the results of the ANCOVA models testing for group differences at the T2 and T3 assessments, including effect size measurements. Seven of the twelve omnibus tests at T2 (prevention/helping behaviors, back pain knowledge, functionality and quality of life, PAM, self-efficacy, intentions, and SOPA control scale) and eight of the twelve omnibus tests at T3 (prevention/helping behaviors, back pain knowledge, functionality and quality of life, PAM, self-efficacy, intentions, SOPA control scale, and Dartmouth COOP) were statistically significant and warranted follow-up comparisons.
All Tx vs. Ctrl follow-up comparisons were statistically significant at T2 and at T3. The T2 comparisons showed five medium effects (η^2=.07–.10) and two small effects (η^2=. 02–.05). The T3 comparisons showed two large effects (both η^2=.15), four medium effects (PAM, η^2=.09–.12) and one small effect (η^2=.03).
Tx vs. AltCtrl showed only three of the seven T2 follow-up comparisons to be statistically significant, and all were associated with small effect sizes (η^2=.01–.02). For the T3 follow-up comparisons, seven of the eight comparisons were statistically significant with small effects sizes (η^2=.02–.05). Examination of observed means shows that in all comparisons, the Tx group showed more favorable mean outcome scores than the AltCtrl group.
Taken together, results of the main effects analyses show that the Tx group outperformed the Ctrl group on most of the study outcome measures at both follow-up assessments. The average effect sizes were η^2=.07 at T2 and η^2=.10 at T3. These results indicate that the intervention effects increased in magnitude through the 4-month follow-up. Results were less robust when compared to the AltCtrl group, but nonetheless, the Tx group did show small positive changes on most of the study outcomes at the 4-month follow-up assessment.
Results of the moderation analysis by gender and age showed no significant condition by sex and age interaction terms at the T2 or T2 assessments. Thus, the FitBack program worked equally well for male and female participants and age was not a factor.
Secondary Analyses
Rates of current back pain were 48%, 54%, and 50% for the Tx, AltCtrl, and Ctrl participants, respectively, at T1 (χ^2[2,597]=1.78, p=.410); 42%, 46%, and 49% at T2 (χ^2[2,597]=2.00, p=.368); and 29%, 41%, and 41% at T3 (χ^2[2,597]=7.61, p=.022). Two contrasts were created: Tx (=0) vs. Ctrl (=1) and Tx (=0) vs. AltCtrl (=1). Logistic regression models were run with the contrasts as the outcomes, with T2 and T3 current back pain scores as predictors, while controlling for T1 current back pain score. Current adjusted back pain status at T2 was not a statistically significant predictor of either contrast. At T3, however, current adjusted back pain status was a significant predictor for both the Tx vs. Ctrl (OR=1.72, 95% CI=1.11-2.68, p=.016) and Tx vs. AltCtrl (OR=1.60, 95% CI=1.03-2.50, p=.035) contrasts. If a study participant reported current back pain at T3, s/he was approximately 1.6 times more likely to be in the AltCtrl group and 1.7 times more likely to be in the Ctrl group than to be in the FitBack Tx group; both odds ratios are associated with small to medium effects.
Examination of log files showed the average total number of minutes spent using the FitBack program by Tx participants was 29.1 (SD=41.8, minimum=0, maximum=341.9). The number of visits to the site ranged from 0 to 87 (M=4.9; SD=4.2). The total time on the site was correlated with T2–T1 and T3–T1 change scores for each of the study outcomes. At T2, more time using the site was significantly correlated with four of the twelve change scores: prevention helping behaviors (r=.15, p=.033), knowledge (r=.21, p=.003), PAM (r=.16, p=.028), and SOPA emotion subscale (r=.18, p=.013). At T3, more time spent using the site was significantly correlated with five of the twelve change scores: knowledge (r=.20, p=.005), self-efficacy (r=.14, p=. 043), SOPA control scale (r=.14, p=.044), WLQ productivity (r=.14, p=.043), and the Dartmouth COOP (r=-.15, p=.034). All correlations were associated with small/medium effects sizes and in the hypothesized direction: more time spent using the site resulted in more favorable change in study outcomes. Comparable time of use data for the AltCtrl participants was not obtainable because these participants received a list of on-line back pain resources embedded in their initial and reminder emails. Click rates recorded by the email host indicated that participants clicked to open 75.6% of the initial emails with that percentage decreasing to 55.8 % of the last reminders. The click rate on one of the included links varied from 29.3% and 31.2% in reminder emails 1 and 2 respectively, to 12.6% in reminder 8. The most popular websites were WebMD Back Pain Health Center (104 clicks), Medline Plus Back Pain (100 clicks), Mayo Clinic (98 clicks), and American Academy of Orthopedic Surgeons (98 clicks).
Indices of user acceptance were all positive. Compared to the AltCtrl participants, FitBack program users had higher satisfaction ratings. The mean total score for FitBack participants was statistically greater at T2 (t[380]=4.40, p<.001, d=.54) and T3 (t[382]=3.51, p<.001, d=.37). The SUS score (M=78.6, SD=15.7), when compared to normative data, is associated with “good” to “excellent” ratings and corresponds to a “B–” (Bangor et al., 2009). For comparison, across 3,500 surveys within 273 studies on different platforms (web, cell phones, TV, etc.), the average SUS score was approximately 70. For web applications, the average SUS score was 68.2 (Bangor et al., 2009). The Perception of Employer Survey results suggest that participants believed they would have a positive impression of employers who made the FitBack program available to employees. They felt that the company would care about them (M=4.7; SD=1.1); they would feel more positive about the company (M=4.6; SD=1.1); they would have greater commitment to the company (M=4.1; SD=1.2); they would be more productive (M=4.1; SD=1.2); and they would feel more job satisfaction (M=4.0; SD=1.3). Results from the Understanding and Implementation Survey indicate that 96%–98% of participants thought they understood the program recommendations for use of heat and ice, over-the-counter medications, exercising to deal with back pain, and relaxation techniques. Implementation of recommendations by participants was reported for relaxation activities (67.8%) and exercises for prevention (78.2%) and dealing with pain occurrences (86%).
18) CONSORT: Results of any other analyses performed, including subgroup analyses and adjusted analyses, distinguishing pre-specified from exploratory

Results

Participants

Participants were 597 workers recruited from our worksite partner (n=244) and the general work population (n=353). Worker job types and other demographic information is shown in Table 1. About half of the participants (51%) indicated they currently had low back pain. Chi-square statistics and associated p-values show groups did not differ on demographic characteristics.

The pain participants reported is described in Table 2 along with the causes to which they ascribed their pain. Table 2 shows the history of back pain items by each of the three study conditions. Chi-square tests were computed to compare groups on all items; no statistically significant differences were found.

Analyses

Primary Analysis

Table 3 provides means and standard deviations for all outcomes at each time point across all three study conditions, and Table 4 provides the results of the ANCOVA models testing for group differences at the T2 and T3 assessments, including effect size measurements. Seven of the twelve omnibus tests at T2 (prevention/helping behaviors, back pain knowledge, functionality and quality of life, PAM, self-efficacy, intentions, and SOPA control scale) and eight of the twelve omnibus tests at T3 (prevention/helping behaviors, back pain knowledge, functionality and quality of life, PAM, self-efficacy, intentions, SOPA control scale, and Dartmouth COOP) were statistically significant and warranted follow-up comparisons.

All Tx vs. Ctrl follow-up comparisons were statistically significant at T2 and at T3. The T2 comparisons showed five medium effects (η^2=.07–.10) and two small effects (η^2=. 02–.05). The T3 comparisons showed two large effects (both η^2=.15), four medium effects (PAM, η^2=.09–.12) and one small effect (η^2=.03).

Tx vs. AltCtrl showed only three of the seven T2 follow-up comparisons to be statistically significant, and all were associated with small effect sizes (η^2=.01–.02). For the T3 follow-up comparisons, seven of the eight comparisons were statistically significant with small effects sizes (η^2=.02–.05). Examination of observed means shows that in all comparisons, the Tx group showed more favorable mean outcome scores than the AltCtrl group.

Taken together, results of the main effects analyses show that the Tx group outperformed the Ctrl group on most of the study outcome measures at both follow-up assessments. The average effect sizes were η^2=.07 at T2 and η^2=.10 at T3. These results indicate that the intervention effects increased in magnitude through the 4-month follow-up. Results were less robust when compared to the AltCtrl group, but nonetheless, the Tx group did show small positive changes on most of the study outcomes at the 4-month follow-up assessment.

Results of the moderation analysis by gender and age showed no significant condition by sex and age interaction terms at the T2 or T2 assessments. Thus, the FitBack program worked equally well for male and female participants and age was not a factor.

Secondary Analyses

Rates of current back pain were 48%, 54%, and 50% for the Tx, AltCtrl, and Ctrl participants, respectively, at T1 (χ^2[2,597]=1.78, p=.410); 42%, 46%, and 49% at T2 (χ^2[2,597]=2.00, p=.368); and 29%, 41%, and 41% at T3 (χ^2[2,597]=7.61, p=.022). Two contrasts were created: Tx (=0) vs. Ctrl (=1) and Tx (=0) vs. AltCtrl (=1). Logistic regression models were run with the contrasts as the outcomes, with T2 and T3 current back pain scores as predictors, while controlling for T1 current back pain score. Current adjusted back pain status at T2 was not a statistically significant predictor of either contrast. At T3, however, current adjusted back pain status was a significant predictor for both the Tx vs. Ctrl (OR=1.72, 95% CI=1.11-2.68, p=.016) and Tx vs. AltCtrl (OR=1.60, 95% CI=1.03-2.50, p=.035) contrasts. If a study participant reported current back pain at T3, s/he was approximately 1.6 times more likely to be in the AltCtrl group and 1.7 times more likely to be in the Ctrl group than to be in the FitBack Tx group; both odds ratios are associated with small to medium effects.

Examination of log files showed the average total number of minutes spent using the FitBack program by Tx participants was 29.1 (SD=41.8, minimum=0, maximum=341.9). The number of visits to the site ranged from 0 to 87 (M=4.9; SD=4.2). The total time on the site was correlated with T2–T1 and T3–T1 change scores for each of the study outcomes. At T2, more time using the site was significantly correlated with four of the twelve change scores: prevention helping behaviors (r=.15, p=.033), knowledge (r=.21, p=.003), PAM (r=.16, p=.028), and SOPA emotion subscale (r=.18, p=.013). At T3, more time spent using the site was significantly correlated with five of the twelve change scores: knowledge (r=.20, p=.005), self-efficacy (r=.14, p=. 043), SOPA control scale (r=.14, p=.044), WLQ productivity (r=.14, p=.043), and the Dartmouth COOP (r=-.15, p=.034). All correlations were associated with small/medium effects sizes and in the hypothesized direction: more time spent using the site resulted in more favorable change in study outcomes. Comparable time of use data for the AltCtrl participants was not obtainable because these participants received a list of on-line back pain resources embedded in their initial and reminder emails. Click rates recorded by the email host indicated that participants clicked to open 75.6% of the initial emails with that percentage decreasing to 55.8 % of the last reminders. The click rate on one of the included links varied from 29.3% and 31.2% in reminder emails 1 and 2 respectively, to 12.6% in reminder 8. The most popular websites were WebMD Back Pain Health Center (104 clicks), Medline Plus Back Pain (100 clicks), Mayo Clinic (98 clicks), and American Academy of Orthopedic Surgeons (98 clicks).

Indices of user acceptance were all positive. Compared to the AltCtrl participants, FitBack program users had higher satisfaction ratings. The mean total score for FitBack participants was statistically greater at T2 (t[380]=4.40, p<.001, d=.54) and T3 (t[382]=3.51, p<.001, d=.37). The SUS score (M=78.6, SD=15.7), when compared to normative data, is associated with “good” to “excellent” ratings and corresponds to a “B–” (Bangor et al., 2009). For comparison, across 3,500 surveys within 273 studies on different platforms (web, cell phones, TV, etc.), the average SUS score was approximately 70. For web applications, the average SUS score was 68.2 (Bangor et al., 2009). The Perception of Employer Survey results suggest that participants believed they would have a positive impression of employers who made the FitBack program available to employees. They felt that the company would care about them (M=4.7; SD=1.1); they would feel more positive about the company (M=4.6; SD=1.1); they would have greater commitment to the company (M=4.1; SD=1.2); they would be more productive (M=4.1; SD=1.2); and they would feel more job satisfaction (M=4.0; SD=1.3). Results from the Understanding and Implementation Survey indicate that 96%–98% of participants thought they understood the program recommendations for use of heat and ice, over-the-counter medications, exercising to deal with back pain, and relaxation techniques. Implementation of recommendations by participants was reported for relaxation activities (67.8%) and exercises for prevention (78.2%) and dealing with pain occurrences (86%).

18-i) Subgroup analysis of comparing only users

Results
Participants
Participants were 597 workers recruited from our worksite partner (n=244) and the general work population (n=353). Worker job types and other demographic information is shown in Table 1. About half of the participants (51%) indicated they currently had low back pain. Chi-square statistics and associated p-values show groups did not differ on demographic characteristics.
The pain participants reported is described in Table 2 along with the causes to which they ascribed their pain. Table 2 shows the history of back pain items by each of the three study conditions. Chi-square tests were computed to compare groups on all items; no statistically significant differences were found.
Analyses
Primary Analysis
Table 3 provides means and standard deviations for all outcomes at each time point across all three study conditions, and Table 4 provides the results of the ANCOVA models testing for group differences at the T2 and T3 assessments, including effect size measurements. Seven of the twelve omnibus tests at T2 (prevention/helping behaviors, back pain knowledge, functionality and quality of life, PAM, self-efficacy, intentions, and SOPA control scale) and eight of the twelve omnibus tests at T3 (prevention/helping behaviors, back pain knowledge, functionality and quality of life, PAM, self-efficacy, intentions, SOPA control scale, and Dartmouth COOP) were statistically significant and warranted follow-up comparisons.
All Tx vs. Ctrl follow-up comparisons were statistically significant at T2 and at T3. The T2 comparisons showed five medium effects ($\eta2$=.07–.10) and two small effects ($\eta2$=. 02–.05). The T3 comparisons showed two large effects (both $\eta2$=.15), four medium effects (PAM, $\eta2$=.09–.12) and one small effect ($\eta2$=.03).
Tx vs. AltCtrl showed only three of the seven T2 follow-up comparisons to be statistically significant, and all were associated with small effect sizes ($\eta2$=.01–.02). For the T3 follow-up comparisons, seven of the eight comparisons were statistically significant with small effects sizes ($\eta2$=.02–.05). Examination of observed means shows that in all comparisons, the Tx group showed more favorable mean outcome scores than the AltCtrl group.
Taken together, results of the main effects analyses show that the Tx group outperformed the Ctrl group on most of the study outcome measures at both follow-up assessments. The average effect sizes were $\eta2$=.07 at T2 and $\eta2$=.10 at T3. These results indicate that the intervention effects increased in magnitude through the 4-month follow-up. Results were less robust when compared to the AltCtrl group, but nonetheless, the Tx group did show small positive changes on most of the study outcomes at the 4-month follow-up assessment.
Results of the moderation analysis by gender and age showed no significant condition by sex and age interaction terms at the T2 or T2 assessments. Thus, the FitBack program worked equally well for male and female participants and age was not a factor.
Secondary Analyses
Rates of current back pain were 48%, 54%, and 50% for the Tx, AltCtrl, and Ctrl participants, respectively, at T1 ($\chi2$[2,597]=1.78, p=.410); 42%, 46%, and 49% at T2 ($\chi2$[2,597]=2.00, p=.368); and 29%, 41%, and 41% at T3 ($\chi2$[2,597]=7.61, p=.022). Two contrasts were created: Tx (=0) vs. Ctrl (=1) and Tx (=0) vs. AltCtrl (=1). Logistic regression models were run with the contrasts as the outcomes, with T2 and T3 current back pain scores as predictors, while controlling for T1 current back pain score. Current adjusted back pain status at T2 was not a statistically significant predictor of either contrast. At T3, however, current adjusted back pain status was a significant predictor for both the Tx vs. Ctrl (OR=1.72, 95% CI=1.11-2.68, p=.016) and Tx vs. AltCtrl (OR=1.60, 95% CI=1.03-2.50, p=.035) contrasts. If a study participant reported current back pain at T3, s/he was approximately 1.6 times more likely to be in the AltCtrl group and 1.7 times more likely to be in the Ctrl group than to be in the FitBack Tx group; both odds ratios are associated with small to medium effects.
Examination of log files showed the average total number of minutes spent using the FitBack program by Tx participants was 29.1 (SD=41.8, minimum=0, maximum=341.9). The number of visits to the site ranged from 0 to 87 (M=4.9; SD=4.2). The total time on the site was correlated with T2–T1 and T3–T1 change scores for each of the study outcomes. At T2, more time using the site was significantly correlated with four of the twelve change scores: prevention helping behaviors (r=.15, p=.033), knowledge (r=.21, p=.003), PAM (r=.16, p=.028), and SOPA emotion subscale (r=.18, p=.013). At T3, more time spent using the site was significantly correlated with five of the twelve change scores: knowledge (r=.20, p=.005), self-efficacy (r=.14, p=. 043), SOPA control scale (r=.14, p=.044), WLQ productivity (r=.14, p=.043), and the Dartmouth COOP (r=-.15, p=.034). All correlations were associated with small/medium effects sizes and in the hypothesized direction: more time spent using the site resulted in more favorable change in study outcomes.
Comparable time of use data for the AltCtrl participants was not obtainable because these participants received a list of on-line back pain resources embedded in their initial and reminder emails. Click rates recorded by the email host indicated that participants clicked to open 75.6% of the initial emails with that percentage decreasing to 55.8 % of the last reminders. The click rate on one of the included links varied from 29.3% and 31.2% in reminder emails 1 and 2 respectively, to 12.6% in reminder 8. The most popular websites were WebMD Back Pain Health Center (104 clicks), Medline Plus Back Pain (100 clicks), Mayo Clinic (98 clicks), and American Academy of Orthopedic Surgeons (98 clicks).
Indices of user acceptance were all positive. Compared to the AltCtrl participants, FitBack program users had higher satisfaction ratings. The mean total score for FitBack participants was statistically greater at T2 (t[380]=4.40, p<.001, d=.54) and T3 (t[382]=3.51, p<.001, d=.37). The SUS score (M=78.6, SD=15.7), when compared to normative data, is associated with “good” to “excellent” ratings and corresponds to a “B–” (Bangor et al., 2009). For comparison, across 3,500 surveys within 273 studies on different platforms (web, cell phones, TV, etc.), the average SUS score was approximately 70. For web applications, the average SUS score was 68.2 (Bangor et al., 2009). The Perception of Employer Survey results suggest that participants believed they would have a positive impression of employers who made the FitBack program available to employees. They felt that the company would care about them (M=4.7; SD=1.1); they would feel more positive about the company (M=4.6; SD=1.1); they would have greater commitment to the company (M=4.1; SD=1.2); they would be more productive (M=4.1; SD=1.2); and they would feel more job satisfaction (M=4.0; SD=1.3). Results from the Understanding and Implementation Survey indicate that 96%–98% of participants thought they understood the program recommendations for use of heat and ice, over-the-counter medications, exercising to deal with back pain, and relaxation techniques. Implementation of recommendations by participants was reported for relaxation activities (67.8%) and exercises for prevention (78.2%) and dealing with pain occurrences (86%).

19) CONSORT: All important harms or unintended effects in each group

NA

19-i) Include privacy breaches, technical problems

NA

19-ii) Include qualitative feedback from participants or observations from staff/researchers

NA

DISCUSSION

20) CONSORT: Trial limitations, addressing sources of potential bias, imprecision, multiplicity of analyses

20-i) Typical limitations in ehealth trials

Discussion
This randomized effectiveness trial program indicates that FitBack positively affected self treatment and prevention behaviors compared to the AltCtrl and Ctrl groups, and it was well received. The results detailed above demonstrate that use of Internet resources to deal with NLBP (AltCtrl) has some value over usual care (Ctrl), but a tailored behaviorally focused intervention (Tx) has greater value. It is unclear whether the average use of 29 minutes of FitBack over five visits will have lasting benefits, but the self-tailored assets might facilitate return visits if users perceived that they provided pain relief or were useful to prevent future pain. Measures of worker productivity and presenteeism are inconclusive because while the results showed significant differences at T2, the effects did not hold at T3. Given the loss of productivity by workers due to back pain [6,7,10, 11], employers potentially might be attracted to an application such as FitBack.
As indicated by the dose-response analysis, greater program use resulted in greater change in outcomes by the Tx group. Understanding a dose response relationship between improved outcomes and program use is, however, complicated. Participant attrition might affect measurements reported in the literature [125], and Doklin and colleagues (2013) raise questions about assumptions of a linear relationship between usage and outcomes. The influence of time of program use on engagement and intervention effectiveness is unclear (Doklin et al (2013), and research is needed to help tease out techniques to improve outcomes [125].
The research reported here adds to the literature on recruitment success of on-line research studies. A total of 3570 views of the informational website led to 2463 responses to the first screening question (Figure 2), and 1932 respondents submitted the on-line screening questionnaire (78.4%), which is substantially more than the 17.3% who submitted the on-line screening for an exercise study with sedentary older adults (Irvine et al., 2013). Also of potential interest to other researchers is the incidence of fraud reported here. Of the 675 individuals who screened, 12 (1.8%) were dropped because of fraudulent information, which is much less than the 9% dropped for fraud by Irvine and colleagues 2013. Across all our on-line studies, we have identified roughly 4% of those who initially qualify to be fraudulent because they supply inaccurate personal information to be accepted as a research subject. We believe that the potential for fraudulent participation in Internet research studies is an important issue, but few researchers report on it.
Limitations
The results reported here must be viewed cautiously because we believe this to be the first attempt to influence NLBP with an on-line intervention. We cannot gauge the importance of the email reminders, which potentially could influence the response rate, on the results (Schneider et al., 2013), and we only prompted the Tx group if they did not open the first message which might have biased the response rate. Additionally, we cannot verify that participants provided accurate information on the surveys, and the 4-month follow-up period was somewhat limited. One to two year follow-up studies, perhaps combined with medical verification, would provide greater confidence in the intervention effects. Also, we cannot determine whether social desirability bias might have influenced responses to assessment items, as has been reported elsewhere [Fisher, 1993].
Research is needed to determine whether the results presented here generalize to other demographic categories. Participants tended to be employed, educated, with at least a middle-class income. Less educated, lower income, and rural populations might be less likely to have Internet in their homes [Zickuhr K, Smith A. 2012.], and FitBack would obviously be inapplicable for those who do not use computers or smart phones.
Conclusions
Despite all these potential shortcomings, however, Tx participants showed significant improvement compared to both the AltCtrl and the Ctrl groups, which is a promising outcome. This research demonstrates that a theoretically based stand-alone responsive mobile-web intervention that tailors content to users' preferences and interests can improve self treatment and motivate behaviors to help prevent future NLBP. This type of intervention is a potentially cost-effective self help tool that can reach large numbers of people. The results are impressive considering that the study was not conducted as part of a larger health promotion campaign, which might have provided additional support and encouragement for the participants. Still, more research is needed on how this type of intervention will be used over time and to understand factors associated with continuing user engagement.

21) CONSORT: Generalisability (external validity, applicability) of the trial findings

21-i) Generalizability to other populations

Discussion
This randomized effectiveness trial program indicates that FitBack positively affected self treatment and prevention behaviors compared to the AltCtrl and Ctrl groups, and it was well received. The results detailed above demonstrate that use of Internet resources to deal with NLBP (AltCtrl) has some value over usual care (Ctrl), but a tailored behaviorally focused intervention (Tx) has greater value. It is unclear whether the average use of 29 minutes of FitBack over five visits will have lasting benefits, but the self-tailored assets might facilitate return visits if users perceived that they provided pain relief or were useful to prevent future pain. Measures of worker productivity and presenteeism are inconclusive because while the results showed significant differences at T2, the effects did not hold at T3. Given the loss of productivity by workers due to back pain [6,7,10, 11], employers potentially might be attracted to an application such as FitBack.
As indicated by the dose-response analysis, greater program use resulted in greater change in outcomes by the Tx group. Understanding a dose response relationship between improved outcomes and program use is, however, complicated. Participant attrition might affect measurements reported in the literature [125], and Doklin and colleagues (2013) raise questions about assumptions of a linear relationship between usage and outcomes. The influence of time of program use on engagement and intervention effectiveness is unclear (Doklin et al (2013), and research is needed to help tease out techniques to improve outcomes [125].
The research reported here adds to the literature on recruitment success of on-line research studies. A total of 3570 views of the informational website led to 2463 responses to the first screening question (Figure 2), and 1932 respondents submitted the on-line screening questionnaire (78.4%), which is substantially more than the 17.3% who submitted the on-line screening for an exercise study with sedentary older adults (Irvine et al., 2013). Also of potential interest to other researchers is the incidence of fraud reported here. Of the 675 individuals who screened, 12 (1.8%) were dropped because of fraudulent information, which is much less than the 9% dropped for fraud by Irvine and colleagues 2013. Across all our on-line studies, we have identified roughly 4% of those who initially qualify to be fraudulent because they supply inaccurate personal information to be accepted as a research subject. We believe that the potential for fraudulent participation in Internet research studies is an important issue, but few researchers report on it.
Limitations
The results reported here must be viewed cautiously because we believe this to be the first attempt to influence NLBP with an on-line intervention. We cannot gauge the importance of the email reminders, which potentially could influence the response rate, on the results (Schneider et al., 2013), and we only prompted the Tx group if they did not open the first message which might have biased the response rate. Additionally, we cannot verify that participants provided accurate information on the surveys, and the 4-month follow-up period was somewhat limited. One to two year follow-up studies, perhaps combined with medical verification, would provide greater confidence in the intervention effects. Also, we cannot determine whether social desirability bias might have influenced responses to assessment items, as has been reported elsewhere [Fisher, 1993].
Research is needed to determine whether the results presented here generalize to other demographic categories. Participants tended to be employed, educated, with at least a middle-class income. Less educated, lower income, and rural populations might be less likely to have Internet in their homes [Zickuhr K, Smith A. 2012.], and FitBack would obviously be inapplicable for those who do not use computers or smart phones.
Conclusions
Despite all these potential shortcomings, however, Tx participants showed significant improvement compared to both the AltCtrl and the Ctrl groups, which is a promising outcome. This research demonstrates that a theoretically based stand-alone responsive mobile-web intervention that tailors content to users' preferences and interests can improve self treatment and motivate behaviors to help prevent future NLBP. This type of intervention is a potentially cost-effective self help tool that can reach large numbers of people. The results are impressive considering that the study was not conducted as part of a larger health promotion campaign, which might have provided additional support and encouragement for the participants. Still, more research is needed on how this type of intervention will be used over time and to understand factors associated with continuing user engagement.

21-ii) Discuss if there were elements in the RCT that would be different in a routine application setting

Discussion
This randomized effectiveness trial program indicates that FitBack positively affected self treatment and prevention behaviors compared to the AltCtrl and Ctrl groups, and it was well received. The results detailed above demonstrate that use of Internet resources to deal with NLBP (AltCtrl) has some value over usual care (Ctrl), but a tailored behaviorally focused intervention (Tx) has greater value. It is unclear whether the average use of 29 minutes of FitBack over five visits will have lasting benefits, but the self-tailored assets might facilitate return visits if users perceived that they provided pain relief or were useful to prevent future pain. Measures of worker productivity and presenteeism are inconclusive because while the results showed significant differences at T2, the effects did not hold at T3. Given the loss of productivity by workers due to back pain [6,7,10, 11], employers potentially might be attracted to an application such as FitBack.
As indicated by the dose-response analysis, greater program use resulted in greater change in outcomes by the Tx group. Understanding a dose response relationship between improved outcomes and program use is, however, complicated. Participant attrition might affect measurements reported in the literature [125], and Doklin and colleagues (2013) raise questions about assumptions of a linear relationship between usage and outcomes. The influence of time of program use on engagement and intervention effectiveness is unclear (Doklin et al (2013), and research is needed to help tease out techniques to improve outcomes [125].
The research reported here adds to the literature on recruitment success of on-line research studies. A total of 3570 views of the informational website led to 2463 responses to the first screening question (Figure 2), and 1932 respondents submitted the on-line screening questionnaire (78.4%), which is substantially more than the 17.3% who submitted the on-line screening for an exercise study with sedentary older adults (Irvine et al., 2013). Also of potential interest to other researchers is the incidence of fraud reported here. Of the 675 individuals who screened, 12 (1.8%) were dropped because of fraudulent information, which is much less than the 9% dropped for fraud by Irvine and colleagues 2013. Across all our on-line studies, we have identified roughly 4% of those who initially qualify to be fraudulent because they supply inaccurate personal information to be accepted as a research subject. We believe that the potential for fraudulent participation in Internet research studies is an important issue, but few researchers report on it.
Limitations
The results reported here must be viewed cautiously because we believe this to be the first attempt to influence NLBP with an on-line intervention. We cannot gauge the importance of the email reminders, which potentially could influence the response rate, on the results (Schneider et al., 2013), and we only prompted the Tx group if they did not open the first message which might have biased the response rate. Additionally, we cannot verify that participants provided accurate information on the surveys, and the 4-month follow-up period was somewhat limited. One to two year follow-up studies, perhaps combined with medical verification, would provide greater confidence in the intervention effects. Also, we cannot determine whether social desirability bias might have influenced responses to assessment items, as has been reported elsewhere [Fisher, 1993].
Research is needed to determine whether the results presented here generalize to other demographic categories. Participants tended to be employed, educated, with at least a middle-class income. Less educated, lower income, and rural populations might be less likely to have Internet in their homes [Zickuhr K, Smith A. 2012.], and FitBack would obviously be inapplicable for those who do not use computers or smart phones.
Conclusions
Despite all these potential shortcomings, however, Tx participants showed significant improvement compared to both the AltCtrl and the Ctrl groups, which is a promising outcome. This research demonstrates that a theoretically based stand-alone responsive mobile-web intervention that tailors content to users' preferences and interests can improve self treatment and motivate behaviors to help prevent future NLBP. This type of intervention is a potentially cost-effective self help tool that can reach large numbers of people. The results are impressive considering that the study was not conducted as part of a larger health promotion campaign, which might have provided additional support and encouragement for the participants. Still, more research is needed on how this type of intervention will be used over time and to understand factors associated with continuing user engagement.

22) CONSORT: Interpretation consistent with results, balancing benefits and harms, and considering other relevant evidence

22-i) Restate study questions and summarize the answers suggested by the data, starting with primary outcomes and process outcomes (use)

Discussion
This randomized effectiveness trial program indicates that FitBack positively affected self treatment and prevention behaviors compared to the AltCtrl and Ctrl groups, and it was well received. The results detailed above demonstrate that use of Internet resources to deal with NLBP (AltCtrl) has some value over usual care (Ctrl), but a tailored behaviorally focused intervention (Tx) has greater value. It is unclear whether the average use of 29 minutes of FitBack over five visits will have lasting benefits, but the self-tailored assets might facilitate return visits if users perceived that they provided pain relief or were useful to prevent future pain. Measures of worker productivity and presenteeism are inconclusive because while the results showed significant differences at T2, the effects did not hold at T3. Given the loss of productivity by workers due to back pain [6,7,10, 11], employers potentially might be attracted to an application such as FitBack.
As indicated by the dose-response analysis, greater program use resulted in greater change in outcomes by the Tx group. Understanding a dose response relationship between improved outcomes and program use is, however, complicated. Participant attrition might affect measurements reported in the literature [125], and Doklin and colleagues (2013) raise questions about assumptions of a linear relationship between usage and outcomes. The influence of time of program use on engagement and intervention effectiveness is unclear (Doklin et al (2013), and research is needed to help tease out techniques to improve outcomes [125].
The research reported here adds to the literature on recruitment success of on-line research studies. A total of 3570 views of the informational website led to 2463 responses to the first screening question (Figure 2), and 1932 respondents submitted the on-line screening questionnaire (78.4%), which is substantially more than the 17.3% who submitted the on-line screening for an exercise study with sedentary older adults (Irvine et al., 2013). Also of potential interest to other researchers is the incidence of fraud reported here. Of the 675 individuals who screened, 12 (1.8%) were dropped because of fraudulent information, which is much less than the 9% dropped for fraud by Irvine and colleagues 2013. Across all our on-line studies, we have identified roughly 4% of those who initially qualify to be fraudulent because they supply inaccurate personal information to be accepted as a research subject. We believe that the potential for fraudulent participation in Internet research studies is an important issue, but few researchers report on it.
Limitations
The results reported here must be viewed cautiously because we believe this to be the first attempt to influence NLBP with an on-line intervention. We cannot gauge the importance of the email reminders, which potentially could influence the response rate, on the results (Schneider et al., 2013), and we only prompted the Tx group if they did not open the first message which might have biased the response rate. Additionally, we cannot verify that participants provided accurate information on the surveys, and the 4-month follow-up period was somewhat limited. One to two year follow-up studies, perhaps combined with medical verification, would provide greater confidence in the intervention effects. Also, we cannot determine whether social desirability bias might have influenced responses to assessment items, as has been reported elsewhere [Fisher, 1993].
Research is needed to determine whether the results presented here generalize to other demographic categories. Participants tended to be employed, educated, with at least a middle-class income. Less educated, lower income, and rural populations might be less likely to have Internet in their homes [Zickuhr K, Smith A. 2012.], and FitBack would obviously be inapplicable for those who do not use computers or smart phones.
Conclusions
Despite all these potential shortcomings, however, Tx participants showed significant improvement compared to both the AltCtrl and the Ctrl groups, which is a promising outcome. This research demonstrates that a theoretically based stand-alone responsive mobile-web intervention that tailors content to users' preferences and interests can improve self treatment and motivate behaviors to help prevent future NLBP. This type of intervention is a potentially cost-effective self help tool that can reach large numbers of people. The results are impressive considering that the study was not conducted as part of a larger health promotion campaign, which might have provided additional support and encouragement for the participants. Still, more research is needed on how this type of intervention will be used over time and to understand factors associated with continuing user engagement.

22-ii) Highlight unanswered new questions, suggest future research

Discussion

This randomized effectiveness trial program indicates that FitBack positively affected self treatment and prevention behaviors compared to the AltCtrl and Ctrl groups, and it was well received. The results detailed above demonstrate that use of Internet resources to deal with NLBP (AltCtrl) has some value over usual care (Ctrl), but a tailored behaviorally focused intervention (Tx) has greater value. It is unclear whether the average use of 29 minutes of FitBack over five visits will have lasting benefits, but the self-tailored assets might facilitate return visits if users perceived that they provided pain relief or were useful to prevent future pain. Measures of worker productivity and presenteeism are inconclusive because while the results showed significant differences at T2, the effects did not hold at T3. Given the loss of productivity by workers due to back pain [6,7,10, 11], employers potentially might be attracted to an application such as FitBack.

As indicated by the dose-response analysis, greater program use resulted in greater change in outcomes by the Tx group. Understanding a dose response relationship between improved outcomes and program use is, however, complicated. Participant attrition might affect measurements reported in the literature [125], and Doklin and colleagues (2013) raise questions about assumptions of a linear relationship between usage and outcomes. The influence of time of program use on engagement and intervention effectiveness is unclear (Doklin et al (2013), and research is needed to help tease out techniques to improve outcomes [125].

The research reported here adds to the literature on recruitment success of on-line research studies. A total of 3570 views of the informational website led to 2463 responses to the first screening question (Figure 2), and 1932 respondents submitted the on-line screening questionnaire (78.4%), which is substantially more than the 17.3% who submitted the on-line screening for an exercise study with sedentary older adults (Irvine et al., 2013). Also of potential interest to other researchers is the incidence of fraud reported here. Of the 675 individuals who screened, 12 (1.8%) were dropped because of fraudulent information, which is much less than the 9% dropped for fraud by Irvine and colleagues 2013. Across all our on-line studies, we have identified roughly 4% of those who initially qualify to be fraudulent because they supply inaccurate personal information to be accepted as a research subject. We believe that the potential for fraudulent participation in Internet research studies is an important issue, but few researchers report on it.

Limitations

The results reported here must be viewed cautiously because we believe this to be the first attempt to influence NLBP with an on-line intervention. We cannot gauge the importance of the email reminders, which potentially could influence the response rate, on the results (Schneider et al., 2013), and we only prompted the Tx group if they did not open the first message which might have biased the response rate. Additionally, we cannot verify that participants provided accurate information on the surveys, and the 4-month follow-up period was somewhat limited. One to two year follow-up studies, perhaps combined with medical verification, would provide greater confidence in the intervention effects. Also, we cannot determine whether social desirability bias might have influenced responses to assessment items, as has been reported elsewhere [Fisher, 1993].

Research is needed to determine whether the results presented here generalize to other demographic categories. Participants tended to be employed, educated, with at least a middle-class income. Less educated, lower income, and rural populations might be less likely to have Internet in their homes [Zickuhr K, Smith A. 2012.], and FitBack would obviously be inapplicable for those who do not use computers or smart phones.

Conclusions

Despite all these potential shortcomings, however, Tx participants showed significant improvement compared to both the AltCtrl and the Ctrl groups, which is a promising outcome. This research demonstrates that a theoretically based stand-alone responsive mobile-web intervention that tailors content to users' preferences and interests can improve self treatment and motivate behaviors to help prevent future NLBP. This type of intervention is a potentially cost-effective self help tool that can reach large numbers of people. The results are impressive considering that the study was not conducted as part of a larger health promotion campaign, which might have provided additional support and encouragement for the participants. Still, more research is needed on how this type of intervention will be used over time and to understand factors associated with continuing user engagement.

Other information

23) CONSORT: Registration number and name of trial registry

Clinicaltrials.gov NCT01950091

24) CONSORT: Where the full trial protocol can be accessed, if available

na

25) CONSORT: Sources of funding and other support (such as supply of drugs), role of funders

This research was funded by a grant to the first author from the US National Institutes of Health, National Institute of Arthritis and Musculoskeletal and Skin Diseases (R44AR054652). The project

X26-i) Comment on ethics committee approval

After approval by an Institutional Review Board for protection of human subjects (IRB), the study was conducted entirely on the Internet, with recruitment and assessments hosted by surveyconsole.com, a provider of on-line survey tools. The study was conducted in partnership with a large health insurer who promoted the project to client companies.

x26-ii) Outline informed consent procedures

After approval by an Institutional Review Board for protection of human subjects (IRB), the study was conducted entirely on the Internet, with recruitment and assessments hosted by surveyconsole.com, a provider of on-line survey tools. The study was conducted in partnership with a large health insurer who promoted the project to client companies.

X26-iii) Safety and security procedures

After approval by an Institutional Review Board for protection of human subjects (IRB), the study was conducted entirely on the Internet, with recruitment and assessments hosted by surveyconsole.com, a provider of on-line survey tools. The study was conducted in partnership with a large health insurer who promoted the project to client companies.

X27-i) State the relation of the study team towards the system being evaluated

was the grant PI. He was employed as a Research Scientist at ORCAS, a healthcare technology company that creates self-management programs to improve physical and emotional well-being. Software development was funded with an SBIR grant, which was designed to stimulate research and product development. Thus, improved versions of FitBack will be marketed. Dr. Irvine retired in November 2013, and he and the other authors derived no financial benefit from sales or from publication of this research.
